# Lysine Acetylation Controls Local Protein Conformation by Influencing Proline Isomerization

**DOI:** 10.1016/j.molcel.2014.07.004

**Published:** 2014-09-04

**Authors:** Françoise S. Howe, Ivan Boubriak, Matthew J. Sale, Anitha Nair, David Clynes, Anne Grijzenhout, Struan C. Murray, Ronja Woloszczuk, Jane Mellor

**Affiliations:** 1 Department of Biochemistry, University of Oxford, South Parks Road, Oxford OX1 3QU, UK

## Abstract

Gene transcription responds to stress and metabolic signals to optimize growth and survival. Histone H3 (H3) lysine 4 trimethylation (K4me3) facilitates state changes, but how levels are coordinated with the environment is unclear. Here, we show that isomerization of H3 at the alanine 15-proline 16 (A15-P16) peptide bond is influenced by lysine 14 (K14) and controls gene-specific K4me3 by balancing the actions of Jhd2, the K4me3 demethylase, and Spp1, a subunit of the Set1 K4 methyltransferase complex. Acetylation at K14 favors the A15-P16*trans* conformation and reduces K4me3. Environmental stress-induced genes are most sensitive to the changes at K14 influencing H3 tail conformation and K4me3. By contrast, ribosomal protein genes maintain K4me3, required for their repression during stress, independently of Spp1, K14, and P16. Thus, the plasticity in control of K4me3, via signaling to K14 and isomerization at P16, informs distinct gene regulatory mechanisms and processes involving K4me3.

## Highlights

• H3K14 acetylation influences *cis-trans* isomerization at the H3A15-P16 peptide bond
• H3A15-P16*trans* is associated with H3K14ac and reduced global H3K4me3
• A15-P16*cis-trans* isomerization balances K4me3 (Set1/Spp1) and demethylation (Jhd2)
• K4me3 on RPGs is largely Spp1- and K14/P16-insensitive while ESR genes are dependent

## Graphical Abstract

**Figure:**
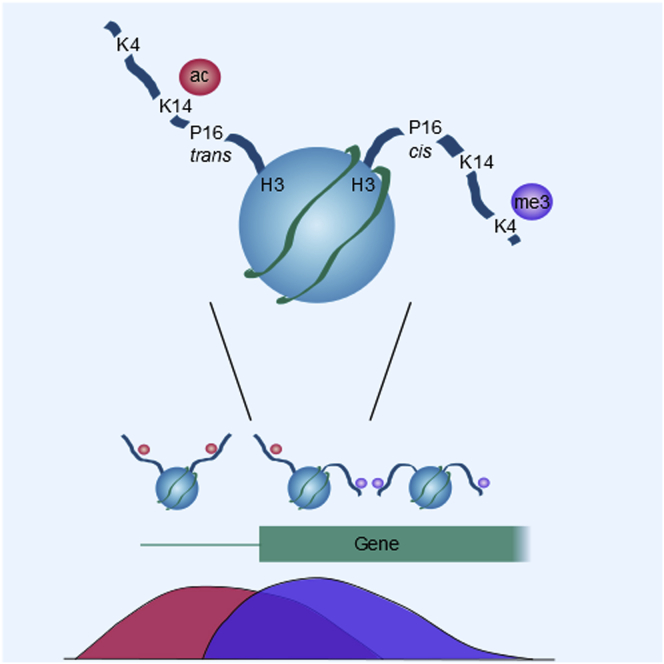

## Teaser

The histone H3 tail is intrinsically disordered yet extensively modified. Howe et al. show that lysine 14 acetylation influences *cis-trans* isomerization at the alanine 15-proline 16 peptide bond to control gene-specific lysine 4 trimethylation. Lysine acetylation in proximity to proline residues may seed alternative conformations in disordered regions, allowing interactions with different effector proteins.

## Introduction

The packaging of eukaryotic genomes into chromatin has fundamental effects on gene expression but how this is brought about still remains poorly understood. Histone proteins are highly conserved and are subject to many different posttranslational modifications (PTMs) including acetylation and methylation (Rando and Winston, 2012). These modifications can influence nucleosome occupancy and position as well as the recruitment of a wide range of effector proteins implicated in a variety of cellular processes. Histone methylation can be influenced by the conformation of the N-terminal region of histone H3, particularly *cis-trans* isomerization of bonds around proline residues (Lu et al., 2007, Nelson et al., 2006, Youdell et al., 2008). The peptidyl-prolyl isomerase (PPIase) Fpr4 increases the rate of *cis-trans* isomerization at all three prolines (16, 30, and 38) on H3 in vitro (Monneau et al., 2013) and H3K37-P38 isomerization influences Set2-mediated H3K36me3 (Nelson et al., 2006).

Genome-wide mapping studies show that modification patterns are correlated with both gene structure and gene activity, often showing characteristic distributions on active or repressed genes (Liu et al., 2005, Pokholok et al., 2005). One such modification is Set1-dependent methylation of lysine 4 on histone H3 (K4), present in most eukaryotes at active or potentially active genes (Santos-Rosa et al., 2002). This has led some to assume that K4 methylation by Set1 is an activating modification yet, in yeast, most evidence points to K4 methylation having a repressive or adaptive role, silencing some rDNA repeats (Briggs et al., 2001, Bryk et al., 2002), repressing genes in midsporulation and during exponential growth (Carvin and Kladde, 2004, Fingerman et al., 2005, Guillemette et al., 2011, Lenstra et al., 2011, Wang et al., 2011), and facilitating the transcriptional response to diamide stress (Weiner et al., 2012). Paradoxically, but consistent with a modulatory role, K4 methylation facilitates the recruitment of both lysine acetyl transferases (KATs) and histone deacetylases (HDACs) (Vermeulen and Timmers, 2010). Set1 is found in a complex (Set1C) with seven other subunits (Swd1, Swd3, Bre2, Sdc1, Spp1, Swd2, and Shg1) (Briggs et al., 2001, Nagy et al., 2002, Roguev et al., 2003) that contribute to complex integrity and/or degree of methylation (K4me1, K4me2 or K4me3). Spp1 is required for K4me3 (Morillon et al., 2005) and contains a PHD motif that interacts with K4me2 and K4me3 (Murton et al., 2010, Shi et al., 2007) thus maintaining high local levels of K4me3. Loss of K4me3 from chromatin involves either histone dilution during DNA replication (Radman-Livaja et al., 2010) or active demethylation by Jhd2 (Ingvarsdottir et al., 2007, Liang et al., 2007, Seward et al., 2007, Tu et al., 2007). Jhd2 is particularly important in sporulation to keep certain genes expressed (Xu et al., 2012). Methylated K4 is influenced by the modification state at distant residues on nucleosomal histones including H2Bub1 and K14ac promoting, and H3R2me and K4ac antagonizing K4me2 and K4me3 to varying extents (Briggs et al., 2002, Guillemette et al., 2011, Kirmizis et al., 2007, Maltby et al., 2012, Nakanishi et al., 2008).

Cells alternate between phases of growth and quiescence, known as the yeast metabolic cycle (YMC) (Tu et al., 2005). Global levels of K4me3 remain fairly constant but levels of H3 acetylation vary, for example on K14 (Cai et al., 2011, Tu et al., 2005). This may reflect fluctuations in the levels of acetyl CoA, a cofactor for KATs, which are lower in quiescent cells. Quiescent cells are more resistant to stress (Lu et al., 2009, Slavov et al., 2012) and express genes that negatively correlate with growth rate (Brauer et al., 2008) and genes whose expression increases during the common environmental stress response (ESR) (Gasch et al., 2000, O’Duibhir et al., 2014). Feeding quiescent cells metabolic intermediates, such as acetate, induces cycling and growth (Cai et al., 2011, Shi and Tu, 2013). Moreover, the lysine acetyltransferase, Gcn5 (KAT2) a component of SAGA (Baker and Grant, 2007), is required for metabolic cycling (Cai et al., 2011). This suggests that acetylation on histone H3 will reflect the metabolic state of the cell and environmental growth-related signals.

As K14 acetylation is reported to be required for K4me3 (Maltby et al., 2012, Nakanishi et al., 2008) and K14 is subject to Gcn5/SAGA-dependent acetylation (Jiang et al., 2007, Zhang et al., 1998), we wanted to explore in more detail the relationship between K14 and K4me3. Here, we show that K14 modulates the conformation of the H3 tail at the alanine 15-proline 16 peptide bond (*cis-trans* isomerization) to control K4me3. Substitutions at K14 (A, R, and Q) and P16 (V and A) to mimic neutral, positively charged, acetylated or *trans* conformation of the H3 tail respectively reveal the different contributions of these residues in promoting Spp1 association with chromatin and antagonizing Jhd2-mediated demethylation of K4me3. K14ac is associated with A15-P16*trans* and reduced K4me3. Strains lacking Spp1 show a similar reduction (≈5-fold decrease) in global K4me3 levels to the K14A strain. This residual level of K4me3 is due to a Spp1-, K14-, and P16-independent mechanism for deposition of K4me3, most evident at the ribosomal protein genes (RPGs). By contrast, environmental stress response (ESR)-induced genes are most dependent on Spp1, K14, and P16 for K4me3. We propose that changes in growth conditions and the availability of metabolic intermediates and regulators allow variable control over levels of K4me3 at different genic loci.

## Results

**Figure 1:**
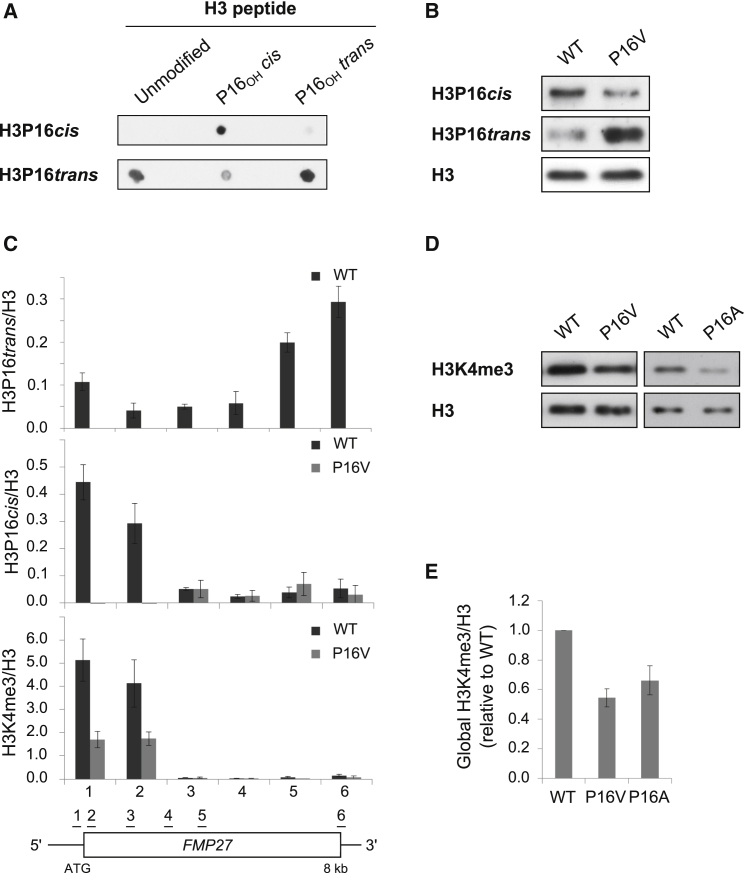
Proline 16 Adopts Two Conformations to Influence K4me3 (A) Unmodified, *cis*-hydroxyproline and *trans*-hydroxyproline peptides (0.1 μg) were dotted onto nitrocellulose and incubated as for western blotting with P16*cis* or P16*trans* antibody purified for 48 hr before incubation. (B) Western blot analysis of purified P16*cis* and P16*trans* antibody binding to WT and P16V whole cell protein extracts. (C) Chromatin immunoprecipitation (ChIP)-qPCR showing the levels of P16*cis*, K4me3, and P16*trans* at *FMP27*, normalized to levels of H3 at positions indicated, ± SEM for three repeats. (D) Western blots showing the reduction in global levels of K4me3 in the P16V and P16A strains relative to WT. (E) Averaged quantitation ± SEM for H3-normalized K4me3 (n = 4–9). See also Figure S1.

Proline is unique in its ability to form *cis* or *trans* peptide bonds with the preceding residue. We were interested in whether, as at P38 (Nelson et al., 2006), *cis-trans* isomerization of P16 is important for H3 tail modifications. For evidence that P16 adopts *cis* or *trans* conformations in vivo, we raised antibodies against short H3 peptides in which the A15-P16 peptidyl-prolyl bond was fixed predominantly in the *cis* or *trans* configuration by hydroxylation of the proline ring (Taylor et al., 2005). After extensive selection for specificity using the peptides (Figure 1A), we obtained antibodies that specifically recognized the peptide with hydroxylated P16 in the desired conformation (the unmodified peptide will have the majority of P16 in *trans*). We further characterized these antibodies by western blot using a WT strain (both *cis* and *trans* conformations) and a strain with P16 substituted for valine, which fixes the A15-V16 bond in *trans* (Lu et al., 2007, Nelson et al., 2006) (Figure 1B). As expected, we observed a higher P16*cis* signal in the WT over the P16V strain but a higher P16*trans* signal in the P16V strain relative to the WT. The antibodies were used to perform a chromatin immunoprecipitation (ChIP) experiment to detect A15-P16 in *cis* and in *trans* in the chromatin over the long gene *FMP27* (Figure 1C and Figure S1A available online; *ADH1*). P16*trans* is low at the 5′ region of *FMP27* and increases toward the 3′ end of the gene. Conversely, we observed P16*cis* at the 5′ region of the genes, with a similar distribution to that we had previously observed for K4me3. We asked whether the integrity of P16 is required for K4me3. Both valine and alanine substitutions at P16 significantly reduced global levels of K4me3 (Figures 1D and 1E). We tested other modifications on H3 but none, apart from K4me3, K14ac, and K18ac, were influenced by the P16 substitution (Figure S1B). Dot blot analysis demonstrated that antibodies raised against K14ac and K18ac were unable to bind peptides containing their epitope and a P16V substitution (Figure S1C), but mass spectrometry revealed the level of K14ac in a P16V strain was roughly equivalent to WT (Figure S1D). This suggests that the antibodies specific for K14ac and K18ac require P16 as part of their epitope. Thus P16 adopts distinct conformations in vivo and is required for optimal levels of K4me3.

### A, Q, and R Substitution at K14 Differently Affect K4me3

**Figure 2:**
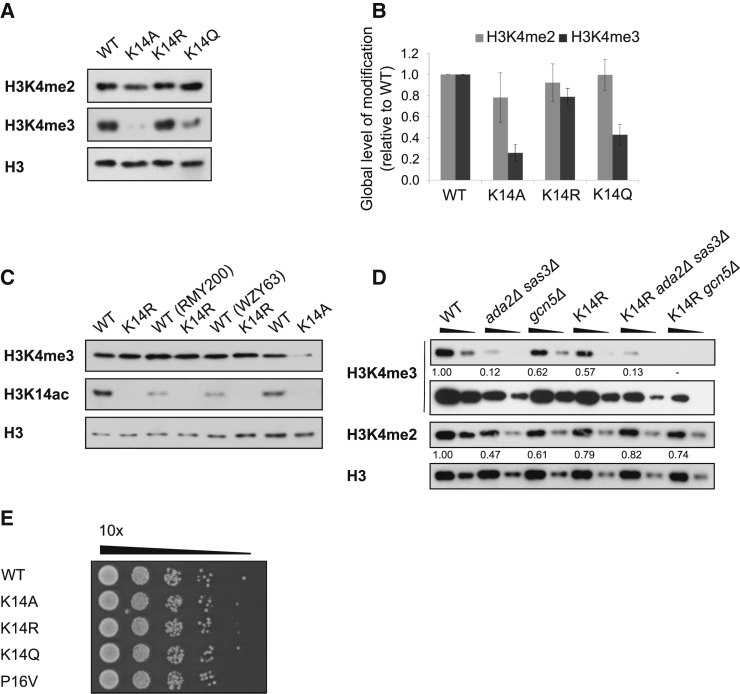
Substitutions at K14 Affect Levels of K4me3 (A) Western blots showing the levels of H3, K4me2, and K4me3 in the WT and K14 substitution strains. (B) Western blot quantitation of H3-normalized K4me2 (n = 4) and K4me3 (n = 9) displayed as mean signals ± SEM. (C) Western blot showing levels of K4me3 in WT and K14R strains from three different yeast backgrounds. K14ac levels control for the K14 mutation. (D) Western blot showing the levels of K4me2 and K4me3 (two exposures) in the *ada2/sas3* and *gcn5* deletion strains. H3-normalized K4 methylation signals are quantified relative to WT. (E) Drop plate growth assay (2 days at 30°C) showing 10-fold serial dilutions on YPD-agar.

It has been reported that substitutions at K14 also result in reduced K4me3 but not K4me1/2 (Nakanishi et al., 2008). Given the effect of P16 substitution on K4me3, we wanted to reexplore the relationship between K14 and K4me3. As described previously, substitution of K14 with alanine (A) or glutamine (Q) causes reductions in the global level of K4me3. In contrast, substitution of K14 with arginine (R) resulted in less severe reductions in global K4me3 (Figures 2A and 2B), confirmed in a range of strain backgrounds (Figure 2C). Lysines for which K to R and K to Q substitutions have opposite effects are known acetylation substrates, such as K14 (Weiner et al., 2012). As there are robust levels of K4me3 in the K14R strain, a lysine at position 14 is not absolutely required for K4me3 and this also rules out a strict dependency on acetylation of K14 for K4me3. Moreover, as the arginine substitution was used to mimic the positively charged, unmodified lysine residue whereas glutamine is a mimic of the neutral, acetylated lysine (Wang and Hayes, 2008), this suggests that unmodified K14 is associated with K4me3.

### Acetylation of K14 Is Not Directly Linked to Trimethylation of Lysine 4

We suggest that K14 in its unmodified state is associated with K4me3, whereas three different groups have concluded, using strains lacking acetyltransferases such as Gcn5 or Sas3, that acetylation is required for K4me3 (Govind et al., 2007, Jiang et al., 2007, Maltby et al., 2012). As these strains have a significantly reduced growth rate (Zhang et al., 1998), it is possible that the reduction in K4me3 is not a direct consequence of reduced K14ac. To test this, *GCN5* or *SAS3* with *ADA2* (Ada2 is required for the KAT activity of Gcn5), were deleted in the K14R strain and a strain with WT histone H3. We note less change in K4me2 levels. Deletion of *GCN5*, or *SAS3* with *ADA2*, reduced the global level of K4me3 in the presence of a WT copy of histone H3, as reported previously. However, deletion of *GCN5* or *SAS3* with *ADA2* in the K14R strain further reduced the level of K4me3 when compared to the control K14R strain (Figure 2D). Because there is already no K14ac in the K14R strain, the additional reduction in K4me3 cannot be due to loss of this modification. Therefore, Gcn5 and Sas3/Ada2 must be able to modulate K4me3 independently of K14ac, possibly via acetylation of other lysines, global changes in transcription or indirectly through the reduced growth rate of the deletion strains. Many gene deletions cause slow growth and result in a transcriptional signature that correlates strongly with the common environmental stress response (ESR) (O’Duibhir et al., 2014). However, there was no obvious difference in growth rate or stress resistance of the K14 and P16 substitution strains compared to WT that might explain reduced K4me3 (Figure 2E). Together, the varying levels of K4me3 in the P16 and the three K14 substitution strains point toward a more modulatory role for these residues rather than a strict requirement for acetylation at K14.

### Distinct Functions for K14 and P16 in Regulating K4me3

**Figure 3:**
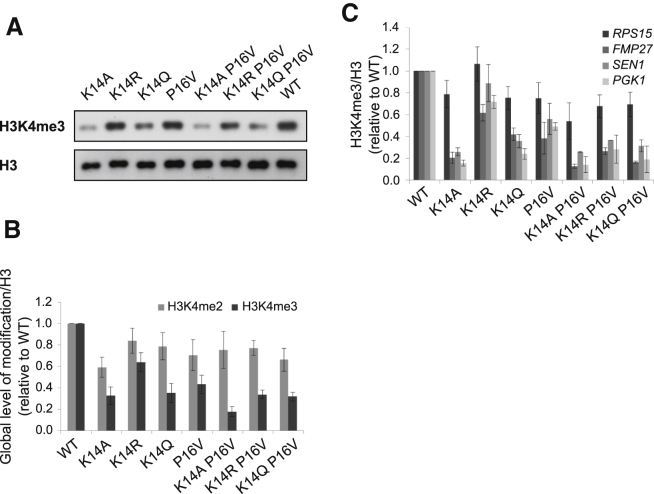
K14 and P16 Have Distinct but Overlapping Effects on K4me3 (A) Western blot showing levels of K4me3 in the WT, K14/P16 single and double substitution strains. (B) H3-normalized K4me2 and K4me3 signals are quantified ± SEM relative to those of the WT strain (n = 4). (C) Averaged ± SEM ChIP-qPCR experiments showing the levels of H3-normalized K4me3 at the 5′ end of four genes (*RPS15*, *SEN1*, *FMP27*, and *PGK1*) in the K14/P16 substitution strains relative to the WT strain (n = 2–8). See also Figure S2.

To investigate if K14 and P16 were influencing K4me3 through a common pathway, K14 P16 double substitution strains were created (no growth defect observed, Figure S2A) and levels of K4me3 were assessed globally or by ChIP at the 5′ ends of *RPS15*, *SEN1*, *FMP27*, and *PGK1* (Figure 3). The global levels (Figures 3A and 3B) and the ChIP signals (Figure 3C) for K4me3 in the K14A P16V and K14Q P16V strains were similar to the K14A and K14Q single substitutions, respectively. By contrast, the signals in the K14R P16V strain were most reduced compared to the single K14R substitution. Together, these findings imply that there may not be a simple linear pathway between K14, P16, and K4me3, perhaps consistent with a structural role for P16 and an additional function at K14. We note that the relative K4me3 levels by ChIP varied at the four genes (Figure 3C), suggesting that genes respond nonuniformly to these substitutions.

### Substitutions at K14 and P16 Differentially Affect the Association of Spp1-HA with Chromatin

**Figure 4:**
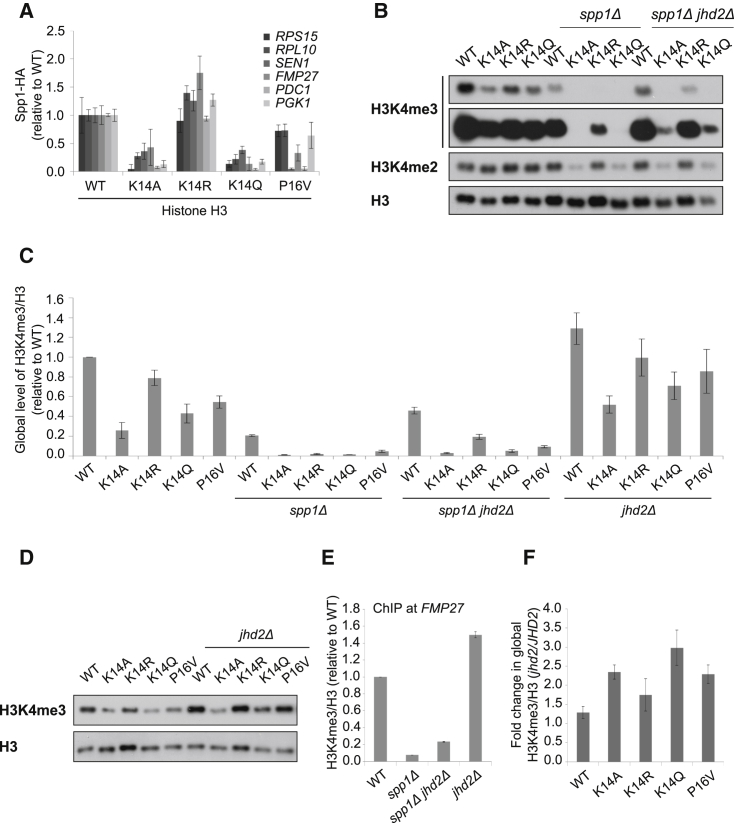
K14 and P16 Control the Balance of Spp1 and Jhd2 Action (A) Levels of Spp1-HA on chromatin at the 5′ ends of *RPS15*, *RPL10*, *SEN1*, *FMP27*, *PGK1*, and *PDC1* in the strains indicated relative to WT. Error bars show SEM of the real-time PCR reaction and are representative of at least three independent experiments. (B) Western blot showing levels of K4me2 and K4me3 (two exposures) in strains indicated. (C) Average quantitation ± SEM (n = 2–4) of H3-normalized K4me3. (D) Western blot showing levels of K4me3 in the K14/P16 substitution strains ± *JHD2*. (E) Average levels ± SEM of H3-normalized K4me3 (n = 2) at the 5′ end of *FMP27* by ChIP-qPCR in strains indicated. (F) Western blot quantitation shows the average ratios of K4me3 in the *jhd2Δ* relative to the *JHD2* strains in the WT histone H3 and K14/P16 substitution strains ± SEM (n = 3). All fold changes in K4me3 are displayed relative to the WT strain. See also Figure S2.

Next, we wanted to uncover more about the mechanism by which the K14 and P16 substitutions modulate levels of K4me3. Because Spp1 is required for K4me3, we asked whether K14 and P16 substitutions influence the association of Spp1 with chromatin. ChIP of Spp1-HA at six genes in the four histone substitution strains revealed levels at or above WT in the K14R strain, reduced levels in the P16V strain and low levels in the K14A and K14Q substitution strains, generally reflecting global levels of K4me3 in these strains (Figure 4A). No change in global Spp1-HA protein levels, or Swd1-HA in the Set1C, is observed in the H3 substitutions (Figure S2B). This supports a role for K14 and P16 in Spp1-dependent K4me3. Once again, as the K14R substitution has near WT levels of both Spp1 chromatin association and global K4me3, acetylation of K14 cannot be required for Spp1 binding and K4me3.

### Substitutions at K14 and P16 Influence the Balance of Spp1 and Jhd2 Activity to Determine Levels of K4me3

We asked whether Spp1 and substitutions at K14/P16 are epistatic by deleting *SPP1* from the K14/P16 substitution strains. Levels of K4me3 were further reduced in the K14/P16-substituted *spp1*Δ strains but remained in proportion to the amount of K4me3 in the individual K14/P16 substitutions when *SPP1* is present (Figures 4B and 4C). This implies that the H3 substitutions must additionally modulate K4me3 via a Spp1-independent pathway. Interestingly, levels of K4me2 are also substantially lower in the K14A *spp1*Δ and K14Q *spp1*Δ strains than in the K14R *spp1*Δ strain, suggesting that these K14 substitutions render the residual K4 methylation in the *spp1*Δ strain more sensitive to demethylation by Jhd2 (Figure 4B). To test this, *JHD2* was deleted in a variety of strains. First, we examined K4me3 in *jhd2*Δ strains with a WT copy of histone H3. An increase in K4me3 was observed upon *JHD2* deletion at both the global level (Figures 4C and 4D) and at the 5′ end of *FMP27* by ChIP (Figure 4E) in the presence or absence of Spp1. This suggests that the sensitivity to Jhd2 is independent of Spp1.

Next, we examined the effects of the *JHD2* deletion in the K14/P16-substituted strains. We note that levels of Jhd2 protein do not change in these strains (Figure S2C). Upon *JHD2* deletion, global levels of K4me3 increased in all histone substitutions (Figures 4C and 4D). To see whether any of the substitutions resulted in a greater increase in K4me3 in the absence of Jhd2 (*jhd2*Δ) compared to the increase in the WT histone H3 strain, we assessed the fold change in K4me3 (Figure 4F). The K14A, K14Q, and P16V strains all showed a larger fold change than observed in the WT or K14R strains, suggesting that these substitutions result in increased action of Jhd2 toward K4me3, accounting in part for the reduced levels compared to wild-type (WT).

Finally, we examined the effects of the double *spp1*Δ*jhd2*Δ deletions in the K14/P16-substituted strains (Figures 4B and 4C). Although levels are low, in all *spp1*Δ*jhd2*Δ strains we observed an increase in K4me3 compared to the *spp1*Δ K14/P16 substitutions. Interestingly, deletion of *JHD2* did not even partially rescue levels of K4me2 in the K14A *spp1*Δ or K14Q *spp1*Δ strains (Figure 4B). Therefore, reduced K4me2 in these strains cannot be explained by increased demethylation, but could instead result from increased histone turnover or from the K14A/Q substitutions compromising the capacity of the Set1C to both di- and trimethylate K4 in the absence of Spp1. Thus the H3 substitutions are altering the balance of K4 methylation by Set1/Spp1 and demethylation by Jhd2 to result in reduced levels of K4me3.

So far we have demonstrated how K14 and P16 are both required for optimal K4me3. These residues influence K4me3 in an overlapping but distinct manner with similar altering of the balance of Spp1 and Jhd2 action. Interestingly, K4me3 is reduced on some genes more than others by these substitutions. Thus, we addressed how changes to the H3 tail around K14 and P16 might influence K4me3 genome-wide.

### K14 and P16 Differentially Regulate the Levels of K4me3 on Functionally Distinct Classes of Genes

To examine the effect of substitutions at K14 and P16 on levels of K4me3 genome-wide, ChIP-sequencing (ChIP-seq) experiments for K4me3 were performed with the WT, K14A, and P16V substitution strains, normalized to H3 levels in each strain. The K4me3 levels in the WT strain correlated well with the previously published data (Kirmizis et al., 2007, Pokholok et al., 2005) (Figure S3A). All nondubious genes (n = 5,771) were ranked according to the ratio (from largest to smallest) of the average K4me3 over the transcription unit in the substitution strains relative to the WT strain (Table S1). Genome-wide, we observed a continuum from least to most affected genes (Figure S3B). There was a positive correlation between the ratio of K4me3 (K14A/WT and P16V/WT) and nascent sense transcription (*r*_S_ = 0.326 and 0.425, respectively), but no strong correlation with gene length (*r*_S_ = −0.063 and −0.003).

**Figure 5:**
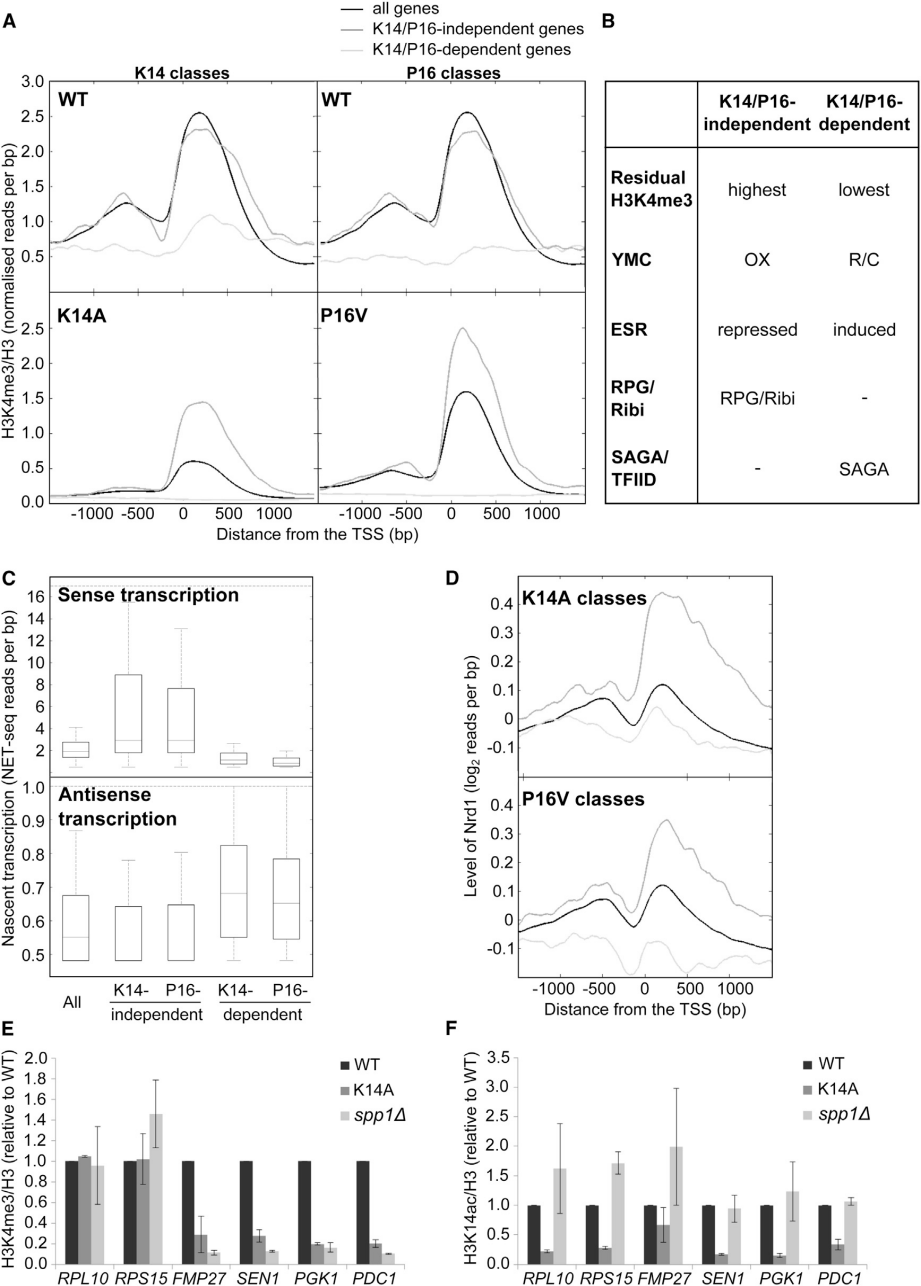
Genome-wide Analysis of K4me3 in the K14A and P16V Strains (A) ChIP-sequencing experiments showing the median distribution of H3-normalized K4me3 around the transcription start site (TSS, 0) in the WT, K14A and P16V strains for all genes (black line) and the 200 most K14/P16-independent (dark gray) and K14/P16-dependent (light gray) genes after ranking according to the ratio of the average level of H3-normalized K4me3 across the transcription unit in the K14A or P16V substitutions relative to the WT strain. (B) Features of genes in the K14/P16-independent and K14/P16-dependent classes (YMC, yeast metabolic cycle; OX, oxidative phase; R/C, reductive charging phase; ESR, environmental stress response; RPGs, ribosomal protein genes; Ribis, ribosome biogenesis genes; SAGA/TFIID-regulated). (C) Boxplots showing the level of WT sense and antisense nascent transcription (Churchman and Weissman, 2011) in the first 300 bp of the transcription unit for the same gene classes as in (A) (outliers excluded). (D) Median WT log2 levels of Nrd1 (Mayer et al., 2012) around the TSS for the same gene classes as in (A). (E and F**)** ChIP-qPCR experiment showing average levels ± SEM of H3-normalized (E) K4me3 and (F) K14ac at the 5′ ends of the indicated genes in rank order (n = 2). See also Tables S1 and S2 and Figure S3.

The 200 genes that maintained the highest K4me3 in the K14A or P16V stains relative to WT were classed as largely K14/P16-independent whereas the 200 genes that lost the most K4me3 in the H3 substitution strains were classed as most K14/P16-dependent (Table S1; Figures 5A and S3B). These classes were used in gene ontology (GO) (Huang et al., 2009a, Huang et al., 2009b) and overlap analyses, which revealed that the K14/P16-independent and K14/P16-dependent genes function in very different biological processes and show different transcriptional dependencies on SAGA (Table S2; Figures 5B and S3C). Genes with the highest ratios of K4me3, in both the K14A and P16V strains relative to WT, are predominantly involved in translation, particularly ribosomal protein genes (RPGs), and are enriched in genes expressed during the oxidative phase of the YMC and repressed during the ESR. In contrast, K14/P16-dependent genes, with K4me3 most reduced by the K14A or P16V substitutions, are predominantly regulated by SAGA, are involved in the response to environmental stresses and metabolic processes, and tend to be induced during the ESR and reductive phase of the YMC. Interestingly, in the P16V substitution strain, the genes with K4me3 most reduced encode proteins involved in sporulation and meiosis, in addition to some of the stress response pathways common to the K14A-affected genes (Table S2). Despite these differences, the Spearman correlation between the ranked genes from the K14A and P16V strains is 0.772. This suggests that although the general trends are similar, K14 and P16 may contribute to different extents to K4me3 levels at individual genes, consistent with both overlapping and distinct functions.

Further analysis of the 200 K14/P16-independent and K14/P16-dependent genes revealed distinct differences (Figures 5C and 5D). In the WT strain, the K14/P16-independent genes are particularly enriched in nascent sense transcription (Churchman and Weissman, 2011) and Nrd1 (Mayer et al., 2012), one of the few genome-wide transcription termination factors shown to be dependent on K4me3 (Schulz et al., 2013, Terzi et al., 2011), in a pattern distinct from the majority of genes. By contrast, the K14/P16-dependent genes are depleted for Nrd1 and nascent sense transcription and enriched for antisense transcription (as expected due to low Nrd1 levels), suggesting distinct regulation of these genes.

### Spp1 and K14 Are Not Essential for K4me3 at Ribosomal Protein Genes

We used ChIP at six genes to validate our genome-wide data and confirm that at two RPGs (*RPS15* and *RPL10*) a strain with a K14A substitution retains high levels of K4me3 (Figure 5E; see also Figure 3C). At four other genes, occupying various positions in the ranking, levels of K4me3 are reduced compared to WT. Given the link between K14 and Spp1, we asked how loss of Spp1 affects K4me3, using ChIP-qPCR at the same six representative genes (Figure 5E). The profile of K4me3 in the *spp1*Δ strain is very similar to the K14A strain. Spp1 is not required for K4me3 at RPGs but is required at the remaining four genes. We observed no significant change in K14ac at these genes in a *spp1*Δ strain that might explain changes in K4me3 (Figure 5F). This suggests that Spp1, like K14, is not essential for K4me3 and further links Spp1 function to the integrity of K14 and P16. We conclude (1) that the integrity of K14 and P16 play a major role in determining K4me3 levels at Spp1-dependent genes, and (2) there may be Spp1-dependent and Spp1-independent forms of Set1C in vivo. Given the similar effects of K14 and P16 substitution on K4me3 on different genes and the results from the K14 P16 double substitution experiments, we wanted to learn more about the relationship between these two residues, particularly how the K14R substitution might differ from other K14 substitutions.

### The Integrity of P16 Affects the Binding of Proteins to Adjacent Sites on the Histone H3 Tail

**Figure 6:**
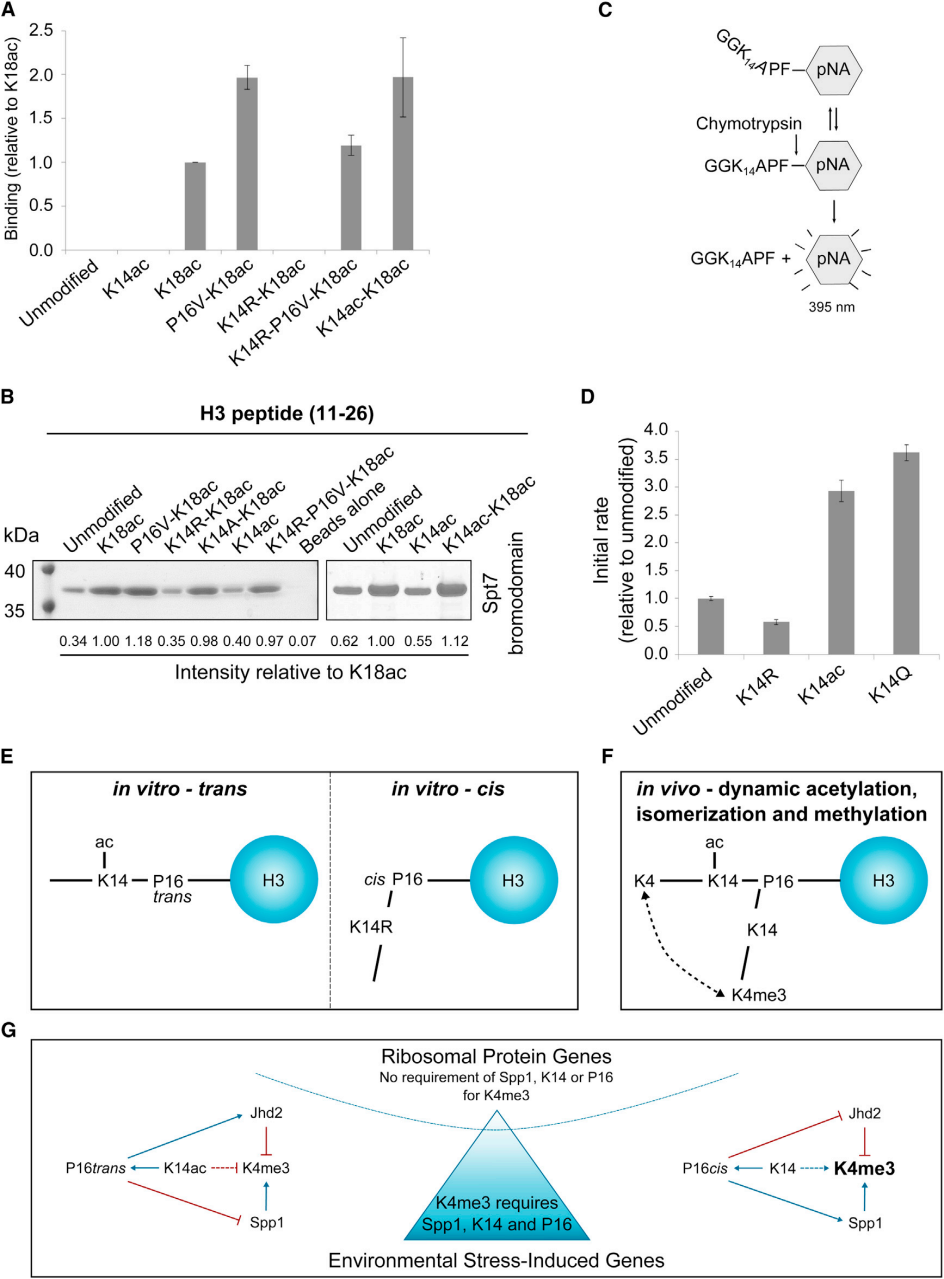
K14ac Promotes P16*trans* (A) Relative binding ± SEM (n = 2–11) assessed by surface plasmon resonance of purified recombinant Spt7 bromodomain (residues 363–619) to the indicated H3 peptides (11–26). (B) Coomassie-stained gel showing a pull-down experiment, quantified relative to the K18ac peptide, with the indicated H3 peptides and the purified recombinant Spt7 bromodomain (n = 3). (C) A chymotrypsin-coupled assay for proline isomers using synthetic hexamers based on GGKAPF-pNA (unmodified). pNA is released by chymotrypsin cleavage only from the *trans*-proline-containing peptide. (D) K14ac and substitutions at K14 influence isomerization at A15-P16 and pNA release, expressed as mean initial rate ± SEM (n = 4–5) over the first 0.6–10.2 s of the reaction. (E) Schematic showing how K14ac or the K14R substitution influence A15-P16 conformation. (F) Schematic showing how dynamic K14ac influences A15-P16 conformation and K4me3 (K4 and K14 denote that these residues are not trimethylated or acetylated respectively). (G) The balance of positive-acting factors (right panel) and negative-acting factors (left panel) controls levels of K4me3 at individual genes. The middle panel shows the continuum in requirement for Spp1, K14, and P16 for K4me3 from no dependency (RPGs) to most dependent (ESR-induced genes). See also Figure S4.

We have already demonstrated that the P16V substitution prevents antibodies raised against K14ac or K18ac from recognizing their epitopes on peptides (see Figure S1C). We developed a second assay that involves monitoring the effect of the P16V substitution on the binding of the Spt7 bromodomain to an H3 peptide modified by acetylation at K14 and/or K18 using surface plasmon resonance (SPR) (Figures 6A, S4A, and S4B) or by pull downs (Figures 6B and S4C). Bromodomains are acetyl-lysine binding domains, found in a number of chromatin-associated proteins (Taverna et al., 2007) including Spt7. The function and binding specificity of the Spt7 bromodomain are currently unknown, but it preferentially interacts with hyperacetylated histones in vitro (Hassan et al., 2007). The Spt7 bromodomain (residues 363–619) specifically recognized acetylated K18, as evidenced by the lack of binding to the unmodified H3 peptide, or a peptide acetylated at K14 (Figures 6A and 6B), or of the bromodomain carrying alanine substitutions in residues known to influence acetyl-lysine binding (Y500, Y520, and N521) (Figure S4C). The binding of the Spt7 bromodomain to the P16V-substituted K18ac peptide improved binding by ∼2-fold. This suggests that binding is influenced by P16 and improved with a *trans* conformation. However, no binding was observed to the K14R-K18ac peptide. We reasoned that this could reflect an effect of the side chain on binding or an influence on the conformation of the A15-P16 bond. To test this, we introduced a valine substitution at P16 in the K14R-K18ac peptide, thereby fixing the A15-V16 peptide bond in the *trans* configuration and restored binding at levels between the P16V-K18ac and K18ac peptides. This suggests that in the K14R-K18ac peptide, the A15-P16 peptidyl-prolyl bond adopts the *cis* configuration, reducing binding of the bromodomain to K18ac, which can be restored by fixing the bond in *trans*. The corollary of this is that acetylation of K14 would correlate with the A15-P16 bond in the *trans* configuration, supporting ideas that the peptidyl-prolyl bond conformation is mainly determined by local effects in proteins (Reimer et al., 1998). We tested this using a peptide acetylated at both K14 and K18 and show binding to the Spt7 bromodomain similar to the P16V-K18ac peptide, consistent with our hypothesis that acetylation of K14 influences the conformation of the A15-P16 peptide bond to favor the *trans* configuration.

### K14ac Influences the Proportion of A15-P16*trans*

To test more directly the influence of substitutions or modifications at K14 on *cis* versus *trans* conformations of the A15-P16 peptide bond, we used an established protease-coupled proline isomerization assay (Fischer et al., 1984) (Figure 6C). We designed four peptides, in which K14 was unmodified or acetylated, or substituted with R or Q and where P16 is followed by a phenylalanine and paranitroaniline (F-pNA) group. Chymotrypsin cleaves these peptides to release pNA only if the A15-P16 peptide bond is in the *trans* conformation and with kinetics that are much faster than the spontaneous rate of *cis-trans* isomerization of the proline. Thus, the initial rate of the chymotrypsin-dependent release of pNA, measured spectrophotometrically at 395 nm, is a function of the amount of peptide with a *trans* proline conformation and for the unmodified peptide is comparable to that reported previously in a similar assay (Nelson et al., 2006). Acetylation of K14 increases the initial rate of cleavage of the peptide 3-fold, indicating that a higher proportion of the A15-P16 peptide bond is in the *trans* conformation (Figure 6D). Our data predict that substitutions at K14 will alter the A15-P16 conformation. The K14Q substitution resulted in an increase in the initial rate of cleavage of the peptide, consistent with an increase in the amount of peptide in the *trans* conformation. Crucially, a K14R substitution reduced the initial rate of cleavage of the peptide below the unmodified peptide consistent with an increase in the *cis* conformation. Taken together these data suggest that (1) the conformation of the A15-P16 peptidyl-prolyl bond influences the binding of protein effectors to histone H3, (2) the conformation of the A15-P16 peptidyl-prolyl bond is likely to be influenced by the nature of the residue at n-2 and that acetylation of K14 promotes the *trans* conformation (Figure 6E), and (3) K4me3 is promoted by unmodified K14 increasing A15-P16*cis* (Figure 6F).

## Discussion

We have shown that acetylation at K14 influences the local conformation of histone H3 at the A15-P16 peptide bond in vitro, promoting a *trans* conformation, and we describe how these changes reduce levels of K4me3 in vivo. We propose that the H3 tail alternates between two dynamic forms in vivo: one with K14ac and P16*trans* and the other with unmodified K14 and P16*cis* (Figure 6F). Moreover, substitutions at K14 and P16 affect the balance of methylation by Set1C/Spp1 and demethylation by Jhd2 to modulate K4me3 levels. We find that the amount of K4me3 lost on genes in strains lacking Spp1 or with substitutions at K14 or P16 is not uniform. This is particularly evident on genes that are differentially regulated during the yeast metabolic cycle and environmental stress response (Figure 6G). We infer at least two distinct mechanisms for deposition of K4me3, one of which is sensitive to loss of Spp1 from Set1C and the conformation of the N-terminal region of histone H3.

Spp1 has been proposed to be critical for stimulating the trimethylase activity of Set1 in Set1C (Takahashi et al., 2009). A remarkable finding from this study is that K4 trimethylation (K4me3) at the RPGs is largely independent of Spp1 and mostly insensitive to the acetylation status of K14 or *cis-trans* isomerization of P16, which may regulate the association of Spp1 with chromatin. This suggests an additional mechanism for stimulating K4me3 that predominates at RPGs but also functions at other genes to varying extents. Set1 contains RRM RNA binding motifs and the binding of RNA by Set1 is proposed to stimulate its K4me3 activity (Schlichter and Cairns, 2005, Trésaugues et al., 2006). High levels of sense transcription, intronic RNA (Weiner et al., 2012), antisense transcripts (Margaritis et al., 2012, Murray et al., 2012), and Nrd1-attenuated noncoding transcripts (Schulz et al., 2013) at RPGs may contribute to Spp1-independent alternative anchoring of Set1 to this gene class.

At the majority of yeast genes, including ESR-induced genes, maintenance of K4me3 depends on the integrity of K14, A15-P16*cis* (bent) and Spp1. These genes have lower levels of sense transcription that may not be sufficient for the deposition of K4me3 by a K14/P16-independent mechanism. Although often thought of as unstructured, ∼50% of the H3 N-terminal tail adopts an α-helical conformation in the nucleosome (Banères et al., 1997). Proline is known to disrupt α helices and may act as hinge points in H3, dramatically affecting the relative trajectories of the flanking helices (Lu et al., 2007) and thus the ability of modifying enzymes to find their substrates. Rather than direct cause and effect, our data support a highly dynamic scenario in which levels of K4me3 are influenced by the action of the Set1C methyltransferase with Spp1 and the Jhd2 demethylase, which in turn are influenced by the modification status of K14 and the conformation of the A15-P16 bond (Figure 6G).

More generally, acetylation in proximity to proline residues may act as a metabolically controlled switch within intrinsically disordered regions of proteins to facilitate the alternative conformations required for interactions with a range of different proteins. Although PPIases promote protein folding by increasing the rate of proline isomerization, in disordered regions the *cis-trans* switch may rely on the natural slower isomerization rate with local PTMs, such as acetylation, influencing one state over the other, perhaps by steric hindrance. In vivo, these states could be captured by binding proteins to induce local folding. In summary, we describe a function for lysine acetylation in determining local protein conformation, in this case histone H3, by directly influencing proline 16 *cis-trans* isomerization. The potential for antibody occlusion in yeast expressing substituted histones or off-target effects resulting from HDAC inhibition preclude further reliable in vivo validation of this relationship, although tools to explore this are being developed.

K4me3 interacts with a variety of protein effectors in yeast (Vermeulen and Timmers, 2010), such as Spp1, the KAT components Yng1 and Sgf29, the chromatin remodeling ATPase Isw1, and the HDAC complex Rpd3L, potentially leading to acetylation or deacetylation of residues, including K14, in the vicinity. The resulting positive and negative feedback loops, coupled to variable concentrations of intracellular NADPH and acetyl CoA during the metabolic cycle and growth, could facilitate dynamic regulation of K14 acetylation/deacetylation, *cis-trans* isomerization at P16, and K4 demethylation/trimethylation at different gene classes, allowing the rapid switching between transcriptional states and adaptation to changing conditions. Differential control of K4me3 could modulate many other processes, including initiation of DNA replication (Kan et al., 2008), initiation of meiotic recombination (Acquaviva et al., 2013, Borde et al., 2009, Sommermeyer et al., 2013), and epigenetic memory (Muramoto et al., 2010). Given the many different enzymes and proteins capable of methylating, demethylating, and interacting with K4me3 in multicellular organisms and the variety of processes associated with K4me3 (Vermeulen and Timmers, 2010), fine tuning mechanisms such as those described here would extend the versatility of this near universal but still poorly understood histone modification.

## Experimental Procedures

*Saccharomyces cerevisiae* strains used in this study are shown in Supplemental Experimental Procedures. Yeast cells were grown at 30°C, shaking at 200 rpm in YPD, to exponential phase (1.25 × 10^7^ cells/ml) for whole cell extracts, subject to western blots, and visualized using chemiluminescence (Pierce) and exposure to X-ray film. Quantitation was performed using ImageJ software. Peptide synthesis and antibody production to raise polyclonal antibodies against P16_OH_*cis* and P16_OH_*trans* peptides were performed by Pacific Immunology and antibody affinity purified with 90 μg P16_OH_*trans* or P16_OH_*cis* peptides, respectively and 90 μg unmodified H3 peptides dotted onto nitrocellulose for 48 hr at 4°C. The P16 proline isomerase assay was performed as described (Shan et al., 1994) using 10 μl of 7.8 mM pNA peptide (Proteogenix) solution per 1 ml assay reaction and pNA release monitored at 395 nm. ChIP-qPCR was performed as described (Morillon et al., 2005). ChIP-seq was performed with 10 ng of immunoprecipitated DNA. DNA was multiplexed during library preparation and subjected to 50 nt paired-end single lane sequencing. Peptides for bromodomain interactions studies were synthesized at GL Biochem and Proteogenix. The peptides used in these experiments had a minimum purity, determined by MALDI-TOF mass spectrometry and HPLC, of 84%. Spt7 bromodomain (amino acids 363–619) was expressed and purified as described (Boubriak et al., 2009). SulfoLink Coupling Resin (Thermo Scientific, Cat. 20401) was used in all peptide pull-downs experiments. Peptides were coupled to the resin according to the manufacturer’s instructions. Resin (200 μl) was incubated with 350 μl of bromodomain (final concentration of 30 μg/ml) for 2 hr at 4°C. After multiple washes with binding buffer, high salt (350 mM NaCl), and TE buffer, bound material was eluted by heating at 80°C with 120 μl LDS (Invitrogen). SPR data for peptides binding to immobilized Spt7 bromodomain was generated on Biacore T100 and T200 instruments at 25°C. Sensorgrams, binding curves, and K_d_ values were analyzed with BIA T100 and T200 evaluation software (GE Healthcare) using a 1:1 binding model. Detailed protocols, including data analysis, can be found in Supplemental Experimental Procedures.

## Accession Numbers

ChIP-seq data are available in the ArrayExpress database (http://www.ebi.ac.uk/arrayexpress) under accession number E-MTAB-2717.

## Supplemental Information

Document S1. Supplemental Experimental Procedures, Figures S1–S4, and Table S2

Table S1. All Nondubious Genes and the 200 Most K14/P16-Independent and K14/P16-Dependent Genes with Ratios of K4me3 K14A or P16V Relative to WT, Related to Figure 5 All nondubious genes were ranked (from largest to smallest) according to the ratio of H3-normalized K4me3 across the transcription unit (transcription start site to transcription termination site) in the K14A or P16V substitution strains relative to WT. The first-ranked gene is therefore defined as the gene that retains the most K4me3 in the H3 substitution strain relative to WT. The 200 most K14/P16-independent and K14/P16-dependent genes used for further analysis are listed under the second tab. To account for global changes in K4me3/H3 in the H3 substitution strains, total read counts from these strains were normalized to the levels of K4me3/H3 as determined by western blotting (Zhang et al., 2011; see Supplemental Information).

Document S2. Article plus Supplemental Information
