# Supplementary material for: Lysine Acetylation Controls Local Protein Conformation by Influencing Proline Isomerization
Source: Mol Cell. 2014 Sep 4;55(5):733–44. doi: 10.1016/j.molcel.2014.07.004 (PMC4157579; doi:10.1016/j.molcel.2014.07.004)
Supplement: Document S1. Supplemental Experimental Procedures, Figures S1–S4, and Table S2 [file mmc1.pdf]

Molecular Cell, Volume *55*

Supplemental Information

Lysine Acetylation Controls Local Protein Conformation by Influencing Proline Isomerization

Françoise S. Howe, Ivan Boubriak, Matthew J. Sale, Anitha Nair, David Clynes, Anne Grijzenhout, Struan C. Murray, Ronja Woloszczuk, and Jane Mellor

Supplemental Figures

Figure S1

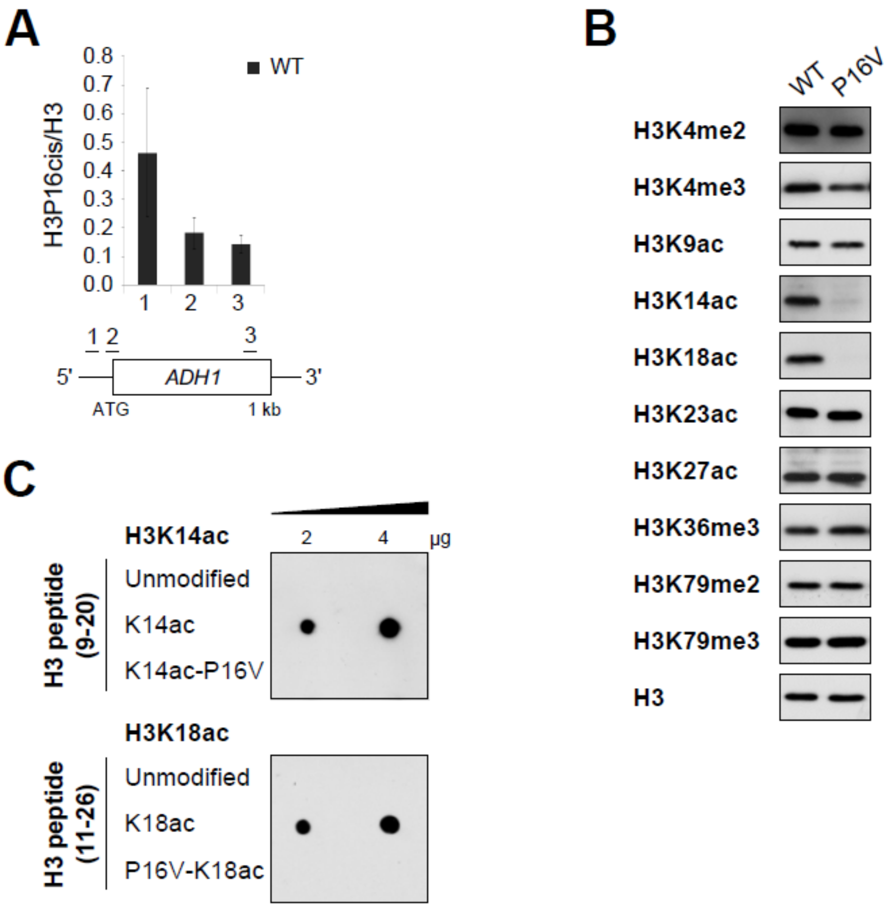

D

	H3 residue number	Observed mass	Mr (experimental)	Mr (calculated)	ppm	Sequence	Modification	Ion score	Peptide intensity
WT	9-17	315.1813	942.5222	942.5247	-2.5	R.KSTGGKAPR.K	ac (K14)	33	7774515
WT	9-17	472.2693	942.5241	942.5247	-0.6	R.KSTGGKAPR.K	ac (K14)	32	31570026
WT	9-17	493.2741	984.5337	984.5352	-1.5	K.KSTGGKAPR.K	2 ac (K9, K14)	51	1040235739
WT	10-17	408.2211	814.4277	814.4297	-2.0	K.STGGKAPR.K	ac (K14)	27	28907756
WT	18-23	358.7187	715.4228	715.4228	0.0	R.KQLASK.A	ac (K18)	37	15473324
WT	18-26	507.8058	1013.597	1013.5981	-1.0	R.KQLASKAAR.K	ac (K23)	52	1045888087
WT	18-26	338.8733	1013.598	1013.5981	-0.1	R.KQLASKAAR.K	ac (K23)	29	918621887
WT	18-26	528.8113	1055.608	1055.6087	-0.7	R.KQLASKAAR.K	2 ac (K18, K23)	56	1268670862
P16V	9-17	473.2771	944.5396	944.5403	-0.7	R.KSTGGKAVR.K	ac (K14)	14	3913804
P16V	9-17	494.2820	986.5494	986.5509	-1.5	R.KSTGGKAVR.K	2 ac (K9, K14)	73	323924046
P16V	10-17	409.2296	816.4446	816.4454	-0.8	K.STGGKAVR.K	ac (K14)	47	1333637
P16V	18-26	507.8060	1013.5974	1013.5981	-0.7	R.KOLASKAAR.K	ac (K23)	9	547378575
P16V	18-26	338.8736	1013.5991	1013.5981	0.9	R.KQLASKAAR.K	ac (K23)	30	471372872
P16V	18-26	528.8119	1055.6093	1055.6087	0.6	R.KQLASKAAR.K	2 ac (K18, K23)	69	24225076

Figure S2

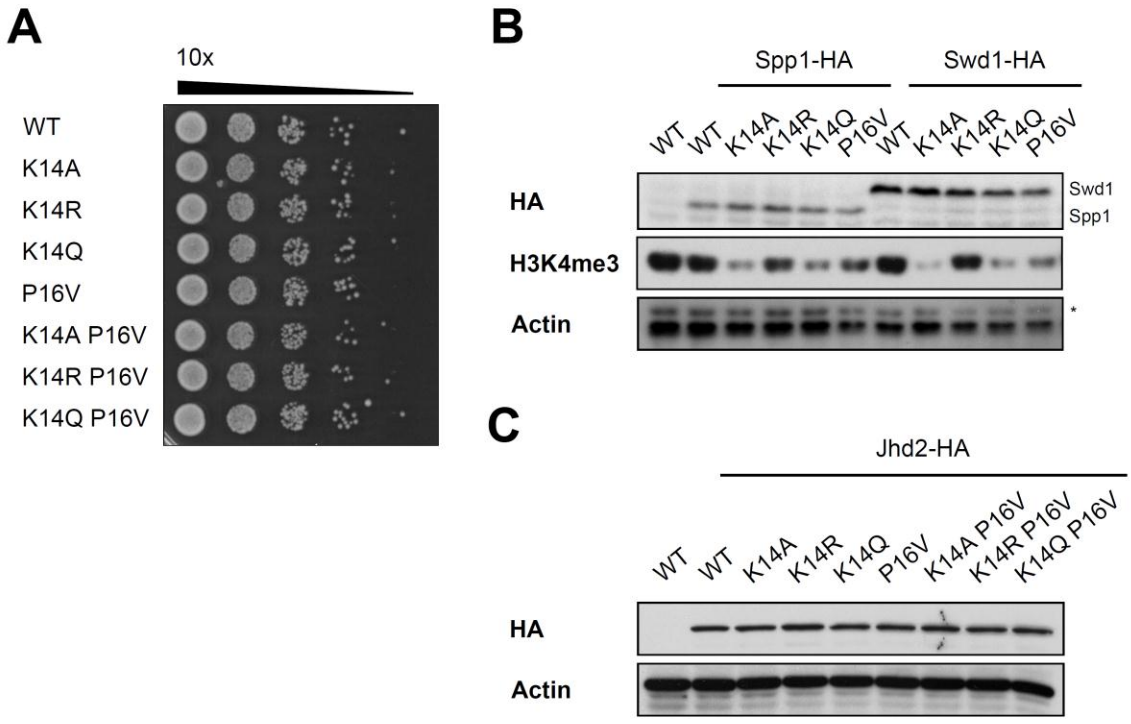
A
10x
WT
K14A
K14R
K14Q
P16V
K14A P16V
K14R P16V
K14Q P16V
B
Spp1-HA
Swd1-HA
WT
WT
K14A
K14R
K14Q
P16V
WT
K14A
K14R
K14Q
P16V
HA
Swd1
Spp1
H3K4me3
Actin
*
C
Jhd2-HA
WT
WT
K14A
K14R
K14Q
P16V
K14A P16V
K14R P16V
K14Q P16V
HA
Actin

Figure S3

A B

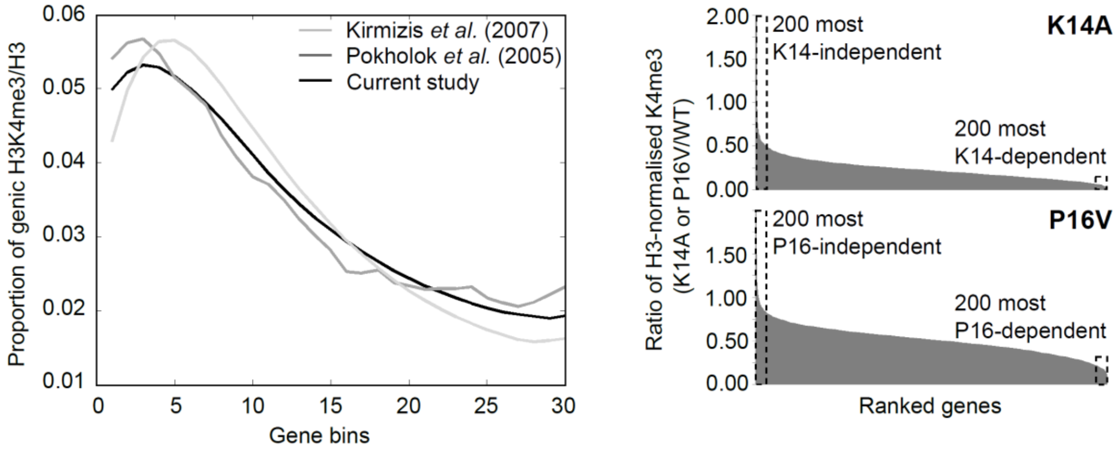

C

% overlap	All genes in study	K14-independent	P16-independent	K14-dependent	P16-dependent
Residual K4me3/H3	-	highest	highest	lowest	lowest
YMC: OX	15.5	**28.5** p = 1 x 10^{-5}	**24.0** p = 3.7 x 10^{-4}	13.5 p = 0.192	8.0 p = 3.5 x 10^{-4}
YMC: R/B	14.8	8.0 p = 1.2 x 10^{-3}	12.0 p = 0.103	12.0 p = 0.102	11.0 p = 4.6 x 10^{-2}
YMC: R/C	23.0	10.0 p < 1.0 x 10^{-5}	8.0 p < 1 x 10^{-5}	**29.5** p = 1.1 x 10^{-2}	**30.0** p = 7.3 x 10^{-3}
ESR: induced	4.5	0.5 p = 1.5 x 10^{-4}	0.5 p = 1.5 x 10^{-4}	**11.5** p < 1.0 x 10^{-5}	**10.5** p = 5.0 x 10^{-5}
ESR: repressed	9.8	**41.0** p < 1.0 x 10^{-5}	**32.5** p < 1.0 x 10^{-5}	0.0 p < 1.0 x 10^{-5}	0.0 p < 1.0 x 10^{-5}
RPG	2.1	**27.5** p < 1.0 x 10^{-5}	**22.5** p < 1.0 x 10^{-5}	0.0 p < 1.0 x 10^{-5}	0.0 p < 1.0 x 10^{-5}
Ribi	2.4	**7.5** p = 3.0 x 10^{-5}	**5.5** p = 3.3 x 10^{-3}	0.0 p < 1.0 x 10^{-5}	0.0 p < 1.0 x 10^{-5}
SAGA-dominant	9.3	1.5 p < 1.0 x 10^{-5}	2.5 p = 5.0 x 10^{-5}	**15.0** p = 9.7 x 10^{-4}	**14.5** p = 2.3 x 10^{-3}
TFIID-dominant	82.4	78.0 p = 0.456	79.5 p = 0.275	53 p < 1.0 x 10^{-5}	48.5 p < 1.0 x 10^{-5}
SAGA/TFIID-dominant	2.7	2.5 p = 0.434	2.0 p = 0.254	**10.0** p < 1.0 x 10^{-5}	**14.0** p < 1.0 x 10^{-5}

Figure S4

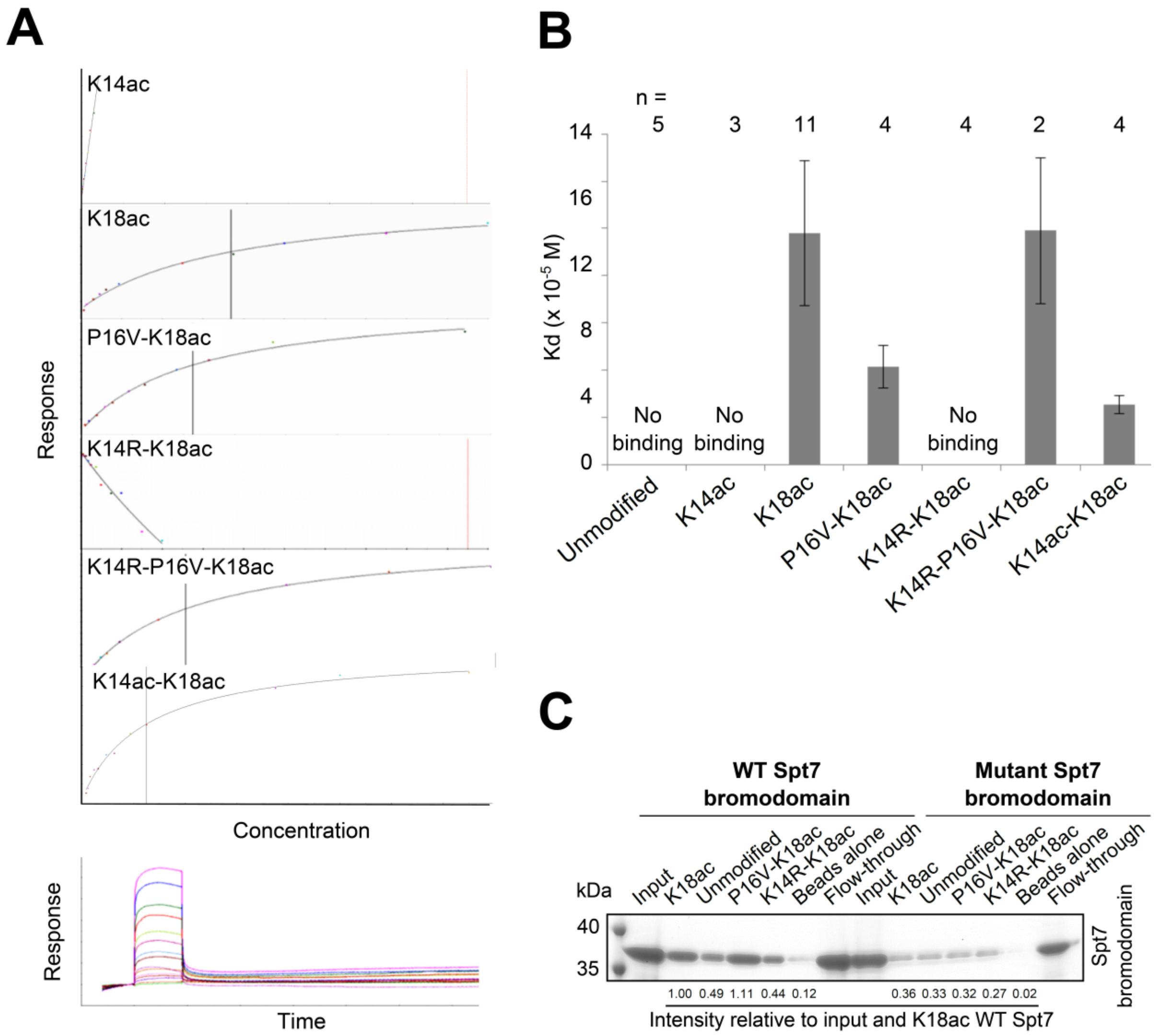
A
K14ac
K18ac
P16V-K18ac
K14R-K18ac
K14R-P16V-K18ac
K14ac-K18ac
Response
Concentration
Response
Time
B
n =
5
3
11
4
4
2
4
14
16
12
8
4
0
Kd (x 10-5 M)
No binding
No binding
No binding
Unmodified
K14ac
K18ac
P16V-K18ac
K14R-K18ac
K14R-P16V-K18ac
K14ac-K18ac
C
WT Spt7 bromodomain
Mutant Spt7 bromodomain
kDa
Input
K18ac
Unmodified
P16V-K18ac
K14R-K18ac
Beads alone
Flow-through
Input
K18ac
Unmodified
P16V-K18ac
K14R-K18ac
Beads alone
Flow-through
40
35
Spt7 bromodomain
1.00 0.49 1.11 0.44 0.12
0.36 0.33 0.32 0.27 0.02
Intensity relative to input and K18ac WT Spt7

Supplemental Figure Legends

Figure S1. (Related to Figure 1)

(A) ChIP-qPCR for H3-normalised P16*cis* at *ADH1.* **(B)** Western blots showing the levels of a range of histone H3 modifications in the WT and P16V strains. Histone H3 levels are shown as a loading control. **(C)** Dot blots of 2 and 4 µg of P16-substituted acetylated H3 peptides encompassing residues 9-20 or 11-26 incubated with antibodies raised to K14ac and K18ac. **(D)** Mass spectrometry data showing the presence of K14ac and K18ac in the P16V strain. Each row displays a separate H3 tryptic peptide. The corresponding H3 residue number, observed mass, experimental molecular weight (Mr), calculated Mr, parts per million (ppm, difference between experimental and calculated mass), peptide sequence, modification, ion score and peak intensity are shown for each peptide.

Figure S2. (Related to Figures 3 and 4)

(A) Drop plate growth assay (2 days at 30°C) showing 10-fold serial dilutions on YPD-agar. **(B-C)** Western blots demonstrating the equal global levels of **(B)** Spp1-HA, Swd1-HA and **(C)** Jhd2-HA in the WT and K14/P16 substitution strains. Actin levels are shown as a loading control (* indicates non-specific band). K4me3 levels are included to control for any changes that might be caused by the HA tagging of Spp1.

Figure S3. (Related to Figure 5)

(A) The average WT distribution profile of K4me3 normalised to histone H3 levels across all genes in *S. cerevisiae* from the present (black), (Pokholok et al., 2005) (dark grey) and (Kirmizis et al., 2007) (light grey) datasets. Genes were divided into 30 bins from the transcription start site to the transcription termination site and the level of K4me3 in each

bin expressed as a proportion of the total K4me3 across the gene. **(B)** All non-dubious genes (n=5771) with the ratios of H3-normalised K4me3 in the K14A and P16V strains relative to the WT strain. Genes with ratios > 2 (n = 7 and 4 for K14A/WT and P16V/WT respectively) have been removed for clarity. Highlighted are the genes with the 200 highest and lowest ratios selected for further analysis (K14/P16-independent and K14/P16-dependent gene classes respectively). **(C)** Overlap (%) between the four selected gene classes from Figure S3B and the genes temporally expressed during the yeast metabolic cycle (YMC: OX, oxidative; R/B, reductive building; R/C, reductive charging phase), induced/repressed by the environmental stress response (ESR), classed as ribosomal protein genes (RPGs) or ribosome biogenesis genes (Ribis), or regulated predominantly by SAGA or TFIID. Bold text indicates significant ($p < 0.05$) enrichment of genes in that class for a particular feature compared to the rest of the genome. P values were calculated from a simulated probability distribution curve. YMC and ESR expression data were taken from (Tu et al., 2005) and (Gasch et al., 2000) respectively. SAGA/TFIID-regulated genes were obtained from (Huisinga and Pugh, 2004).

Figure S4. (Related to Figure 6)

(A) Surface plasmon resonance (SPR) affinity fits for the indicated H3 peptides encompassing residues 11-26 binding to the Spt7 bromodomain (residues 363-619). Also shown is a typical sensorgram of H3 peptide binding to the Spt7 bromodomain demonstrating the low background shift and plateauing upon binding. **(B)** Raw average Kd values ± SEM for Spt7 binding to variously-modified H3 peptides. **(C)** A peptide pull-down experiment with a range of H3 peptides and the WT or substituted Spt7 bromodomain

(Y500A/Y520A/N521A) confirming the requirement for an intact bromodomain for enriched binding to the K18ac over the unmodified peptides.

Table S2. Gene ontology analysis of the 200 most K14/P16-independent and K14/P16-dependent genes (related to Figure 4)

**GO terms for the 200 most K14-independent genes**			
**Biological process**	**Count**	**%**	**P value**
translation	66	33.8	9.20E-16
ribosome biogenesis	42	21.5	1.30E-11
ribonucleoprotein complex biogenesis	44	22.6	2.70E-11
regulation of cellular protein metabolic process	24	12.3	8.40E-06
regulation of translation	22	11.3	2.00E-05
rRNA processing	25	12.8	3.40E-05
post-transcriptional regulation of gene expression	22	11.3	4.20E-05
rRNA metabolic process	25	12.8	5.10E-05
maturation of SSU-rRNA from tricistronic rRNA transcript (SSU-rRNA, 5.8S rRNA, LSU-rRNA)	14	7.2	1.10E-04
maturation of SSU-rRNA	14	7.2	1.30E-04
ribosome assembly	12	6.2	5.00E-04
ncRNA processing	27	13.8	7.30E-04
rRNA export from nucleus	9	4.6	2.00E-03
rRNA transport	9	4.6	2.00E-03
ribonucleoprotein complex assembly	14	7.2	3.20E-03
ncRNA metabolic process	28	14.4	3.50E-03
RNA processing	33	16.9	4.60E-03
RNA export from nucleus	11	5.6	2.40E-02
ribosomal small subunit biogenesis	8	4.1	4.50E-02
translational termination	6	3.1	4.60E-02
ribosomal subunit assembly	8	4.1	4.50E-02
**GO terms for the 200 most P16-independent genes**			
**Biological process**	**Count**	**%**	**P value**
translation	54	27.3	7.70E-08
ribosome biogenesis	33	16.7	1.50E-05
ribonucleoprotein complex biogenesis	34	17.2	5.50E-05
ribosome assembly	11	5.6	1.10E-02
ribonucleoprotein complex assembly	13	6.6	4.70E-02
**GO terms for the 200 most K14-dependent genes**			
**Biological process**	**Count**	**%**	**P value**

thiamine biosynthetic process	7	3.5	5.40E-03
response to temperature stimulus	19	9.6	3.70E-03
thiamine and derivative biosynthetic process	7	3.5	2.50E-03
sulfur compound biosynthetic process	11	5.6	1.90E-03
thiamine metabolic process	7	3.5	2.00E-03
thiamine and derivative metabolic process	7	3.5	2.20E-03
water-soluble vitamin metabolic process	10	5.1	4.40E-03
sulfur metabolic process	12	6.1	4.20E-03
cellular response to heat	16	8.1	4.80E-03
vitamin metabolic process	10	5.1	4.90E-03
vitamin biosynthetic process	9	4.5	8.70E-03
water-soluble vitamin biosynthetic process	9	4.5	8.70E-03
response to heat	16	8.1	1.30E-02
oxidation reduction	22	11.1	1.50E-02
aromatic compound biosynthetic process	7	3.5	2.50E-02
response to abiotic stimulus	21	10.6	2.40E-02
response to toxin	7	3.5	4.60E-02
**GO terms for the 200 most P16-dependent genes**			
**Biological process**	**Count**	**%**	**P value**
sexual sporulation	16	8.2	4.10E-05
sexual sporulation resulting in formation of a cellular spore	16	8.2	4.10E-05
ascospore formation	16	8.2	4.10E-05
thiamine biosynthetic process	8	4.1	7.60E-05
thiamine and derivative biosynthetic process	8	4.1	7.50E-05
thiamine metabolic process	8	4.1	8.10E-05
reproductive developmental process	17	8.8	7.70E-05
thiamine and derivative metabolic process	8	4.1	7.60E-05
reproductive process in single-celled organism	17	8.8	3.90E-04
response to toxin	9	4.6	8.90E-04
reproductive cellular process	19	9.8	1.30E-03
sporulation resulting in formation of a cellular spore	18	9.3	1.20E-03
sporulation	18	9.3	1.20E-03
water-soluble vitamin metabolic process	10	5.2	1.40E-03
sexual reproduction	20	10.3	1.70E-03
aromatic compound biosynthetic process	8	4.1	1.90E-03
vitamin metabolic process	10	5.2	1.80E-03
vitamin biosynthetic process	9	4.6	3.50E-03
water-soluble vitamin biosynthetic process	9	4.6	3.50E-03
reproduction of a single-celled organism	17	8.8	3.30E-03
sulfur metabolic process	11	5.7	4.90E-03
sulfur compound biosynthetic process	9	4.6	8.70E-03
spore wall biogenesis	7	3.6	1.90E-02
spore wall assembly	7	3.6	1.90E-02
ascospore wall assembly	7	3.6	1.90E-02

ascospore wall biogenesis	7	3.6	1.90E-02
cell wall assembly	7	3.6	2.00E-02
response to temperature stimulus	15	7.7	2.10E-02
cellular response to heat	13	6.7	3.80E-02
pyridoxine metabolic process	4	2.1	3.70E-02
vitamin B6 metabolic process	4	2.1	3.70E-02
pyridoxine biosynthetic process	4	2.1	3.70E-02
vitamin B6 biosynthetic process	4	2.1	3.70E-02
fungal-type cell wall biogenesis	7	3.6	4.00E-02

Shown are the significantly enriched ($p < 0.05$) biological processes, corresponding gene counts and P values (Benjamini-Hochberg correction) for the 200 most K14/P16-independent and K14/P16-dependent genes after ranking according to the ratio of H3-normalised K4me3 (K14A or P16V relative to WT). GO analysis was performed using the DAVID functional annotation tool v. 6.7 (Huang da et al., 2009a, b).

Supplementary Experimental Procedures

Strains

S. cerevisiae strains and their genotypes used during this study are shown below.

**Strain**	**Parent**	**Genotype**	**Origin**
WTH3 (referred to as WT throughout)	-	*MAT**a** his3Δ200 leu2Δ0 lys2Δ0 trp1Δ63 ura3Δ0 met15Δ0 can1::MFA1pr-HIS3 hht1-hhf1::NatMX4 hht2-hhf2::[HHTS-HHFS]-URA3*	Open Biosystems
K14A	WTH3	*hht2-hhf2::[HHTS K14A-HHFS]-URA3*	Open Biosystems
K14R	WTH3	*hht2-hhf2::[HHTS K14R-HHFS]-URA3*	Open Biosystems
K14Q	WTH3	*hht2-hhf2::[HHTS K14Q-HHFS]-URA3*	Open Biosystems
RMY200	-	*MATa ade2-101 his3Δ200 lys2-801 trp1-Δ901 ura3-52 hht1 hhf1::LEU2 hht2 hhf2::HIS3 plus pRM200 [CEN4 ARS1 TRP1 HHT2-HHF2]*	(Mann and Grunstein, 1992)
RMY214 (K14R)	RMY200	*plus pRM214 [CEN4 ARS1 TRP1 hht2 K14R-HHF2]*	(Mann and Grunstein, 1992)
WZY63(a)	-	*MATa, ura3–52, lys2–801, ade2–101, trp163, his3200, leu21 hht1-hhf1::pWZ405-F2F9-LEU2,hht2-hhf2::pWZ403-F4F10-HIS3, plus pWZ414-F13 [HHT2-HHF2]*	(Zhang et al., 1998)
WZY67(a) (K14R)	WZY67(a)	*plus pWZ414-F43 [hht2 K14R-HHF2]*	(Zhang et al., 1998)
WT H3/H4	-	*MATa, leu2Δ1, his3Δ200, ura3-52, trp1Δ63, lys2-128δ, (hht1-hhf1)ΔLEU2 (hht2-hhf2)Δ::HIS3 Ty912Δ35-lacZ::his4, [pDM9-HHT1-HHF1-URA3] plus pWZ414-F12 [HHT2-HHF2]*	(Nakanishi et al., 2008)
P16A	WT H3/H4	*plus pWZ414 [hht2 P16A-HHF2]*	(Nakanishi et al., 2008)
P16V	WTH3	*hhts-hhfs::HHTS P16V-HHFS-KanMX6*	This study
K14A P16V	WTH3	*hhts-hhfs::HHTS K14A P16V-HHFS-KanMX6*	This study
K14R P16V	WTH3	*hhts-hhfs::HHTS K14R P16V-HHFS-KanMX6*	This study
K14Q P16V	WTH3	*hhts-hhfs::HHTS K14Q P16V-HHFS-KanMX6*	This study
WTH3 *jhd2Δ*	WTH3	*jhd2::KanMX6*	This study
K14A *jhd2Δ*	K14A	*jhd2::KanMX6*	This study
K14R *jhd2Δ*	K14R	*jhd2::KanMX6*	This study
K14Q *jhd2Δ*	K14Q	*jhd2::KanMX6*	This study
P16V *jhd2Δ*	P16V	*jhd2::LEU2*	This study
WTH3 Jhd2-HA	WTH3	*JHD2-3HA-KanMX6*	This study

K14A Jhd2-HA	K14A	*JHD2-3HA-KanMX6*	This study
K14R Jhd2-HA	K14R	*JHD2-3HA-KanMX6*	This study
K14Q Jhd2-HA	K14Q	*JHD2-3HA-KanMX6*	This study
P16V Jhd2-HA	P16V	*JHD2-3HA-KanMX6*	This study
K14A P16V Jhd2-HA	K14A P16V	*JHD2-3HA-KanMX6*	This study
K14R P16V Jhd2-HA	K14R P16V	*JHD2-3HA-KanMX6*	This study
K14Q P16V Jhd2-HA	K14Q P16V	*JHD2-3HA-KanMX6*	This study
WTH3 *spp1Δ*	WTH3	*spp1::TRP1*	This study
K14A *spp1Δ*	K14A	*spp1::TRP1*	This study
K14R *spp1Δ*	K14R	*spp1::TRP1*	This study
K14Q *spp1Δ*	K14Q	*spp1::TRP1*	This study
P16V *spp1Δ*	P16V	*spp1::TRP1*	This study
WTH3 *spp1Δ jhd2Δ*	WTH3 *jhd2Δ*	*spp1::TRP1 jhd2::KanMX6*	This study
K14A *spp1Δ jhd2Δ*	K14A *jhd2Δ*	*spp1::TRP1 jhd2::KanMX6*	This study
K14R *spp1Δ jhd2Δ*	K14R *jhd2Δ*	*spp1::TRP1 jhd2::KanMX6*	This study
K14Q *spp1Δ jhd2Δ*	K14Q *jhd2Δ*	*spp1::TRP1 jhd2::KanMX6*	This study
P16V *spp1Δ jhd2Δ*	P16V *jhd2Δ*	*spp1::TRP1 jhd2::KanMX6*	This study
WTH3 Spp1-HA	WTH3	*SPP1-3HA-KanMX6*	This study
K14A Spp1-HA	K14A	*SPP1-3HA-KanMX6*	This study
K14R Spp1-HA	K14R	*SPP1-3HA-KanMX6*	This study
K14Q Spp1-HA	K14Q	*SPP1-3HA-KanMX6*	This study
P16V Spp1-HA	P16V	*SPP1-3HA-TRP1*	This study
WTH3 Swd1-HA	WTH3	*SWD1-3HA-KanMX6*	This study
K14A Swd1-HA	K14A	*SWD1-3HA-KanMX6*	This study
K14R Swd1-HA	K14R	*SWD1-3HA-KanMX6*	This study
K14Q Swd1-HA	K14Q	*SWD1-3HA-KanMX6*	This study
P16V Swd1-HA	P16V	*SWD1-3HA-TRP1*	This study

Creation of the K14/P16 double substitution strains

The *HHTS-HHFS* locus was amplified from the Open Biosystems histone substitution strain (Dai et al., 2008). These fragments were then digested with *Eco*RI and *Spe*I at the endogenous restriction sites flanking the locus. The digested fragments were ligated into *Eco*RI/*Spe*I-digested pFA6 (KanMX6) (Longtine et al., 1998). Histone H3 double substitution strains were created using the singly-substituted H3 as templates for Quikchange site-directed mutagenesis (Stratagene). The resulting plasmids were confirmed by sequencing before use as PCR templates to replace the genomic *HHTS-HHFS* with the newly mutated locus. Transformed strains were sequenced once more before use in experiments.

Yeast growth conditions

Yeast cells were grown at 30°C, shaking at 200 rpm to exponential phase in YPD medium (1 % yeast extract (Difco), 1 % bactopeptone supplemented with 2 % glucose). Cells were grown overnight in 5 ml cultures before dilution to 0.2 OD_{600} (0.5 x 10^7 cells/ml) in the appropriate volume of fresh media. Cells were harvested during exponential growth at 0.5 OD_{600} (1.25 x 10^7 cells/ml) by centrifugation (3000 rpm, 5 min). The assessment of yeast growth by drop plate assays was performed by first normalising the cell number in overnight yeast cultures before pipetting 1.5 µl of five serial ten-fold dilutions onto YPD-agar plates. Plates were incubated for 2 days at 30°C.

Preparation of whole cell extracts

Cells were grown at 30°C in 25 ml of the appropriate media to 1.25 x 10^7 cells/ml. Whole cell extracts were prepared by vortexing the cells with glass beads in 300 µl 8 M urea, 240 µl loading buffer (100 mM Tris-Cl pH 6.8, 20 % glycerol, 4 % SDS, 0.1 % bromophenol blue) and 60 µl 1 M DTT for 3 min followed by boiling for 5 min.

Western blotting and dot blots

For Western blotting, proteins were separated on 10-15 % SDS-polyacrylamide gels and transferred to nitrocellulose membranes using a semi-dry transfer method. For dot blots, appropriate amounts of peptide were pipetted onto nitrocellulose membrane and allowed to dry. Membranes were blocked in 5 % bovine serum albumin (BSA) in TBST (20 mM Tris-Cl pH 7.5, 150 mM NaCl, 0.1 % TWEEN-20). Antibodies were added in 2.5 % BSA/TBST. The primary antibodies used are detailed in the table below. The HRP-conjugated rabbit, mouse

or rat secondary antibodies (Sigma) were used at 1:4000 dilutions. Interactions were visualised using chemiluminescence (Pierce) and exposure to X-ray film. Quantitation was performed using ImageJ software and signals were normalised to histone H3 levels and expressed relative to WT.

**Antibody**	**Company/ Catalogue number**	**Species reactivity**	**Dilution for Western blot**	**Dilution for ChIP**
H3	Millipore/07-690	Rabbit	1:2000	1:40
H3K4me2	Millipore/07-030	Rabbit	1:2000	1:40
H3K4me3	Millipore/05-745R	Rabbit	1:2000	1:40
H3K9ac	Millipore/07-352	Rabbit	1:2000	-
H3S10ph	Millipore/05-817	Rabbit	1:2000	-
H3K14ac	Millipore/07-353	Rabbit	1:2000	-
H3P16*cis*	-	Rabbit	1:7500	1:10
H3P16*trans*	-	Rabbit	1:5000	1:6.67
H3K18ac	Millipore/07-354	Rabbit	1:4000	1:40
H3K23ac	Millipore/07-355	Rabbit	1:2000	-
H3K27ac	Millipore/07-360	Rabbit	1:2000	-
H3K36me2	Millipore/07-369	Rabbit	1:2000	-
H3K36me3	Abcam/ab9050	Rabbit	1:2500	1:40
H3K79me2	Abcam/ab3594	Rabbit	1:2000	-
H3K79me3	Abcam/ab2621	Rabbit	1:2000	-
HA (for Western)	Roche/3F10	Rat	1:500	-
HA (for ChIP)	ab9110	Rabbit	-	1:40
Actin	Millipore/MAB1501	Mouse	1:1000	-

Raising polyclonal antibodies against $P16_{OH}$*cis* and $P16_{OH}$*trans* peptides.

Peptide synthesis and antibody production were performed by Pacific Immunology. Peptides were synthesised and the proline hydroxylated to allow the peptides to be enriched with the peptidyl-prolyl bond in either a *cis* or a *trans* conformation: CGKA-(*cis*-hydroxyproline)-RKQLA and CGKA-(*trans*-hydroxyproline)-RKQLA. Each peptide was conjugated to Keyhole Limpet Hemocyanin (KLH) carrier protein via the N-terminal cysteine and used to immunize rabbits. Final bleeds were taken after 105 days and the specificity was increased by purifying the sera against unmodified peptide. Antibodies required extensive

further purification. For the P16*cis* antibody, batches of antibody were purified by incubating 266.66 µl of the Pacific Immunology affinity-purified antibody in 1733.33 µl FA-150 buffer (0.1% SDS, 1% Triton-X-100, 10 mM HEPES, 0.1% sodium deoxycholate, 150 mM NaCl, 1 mM AEBSF, EDTA-free protease inhibitor cocktail (Roche)) with 90 µg $P16_{OH}$*trans* and 90 µg unmodified H3 peptides dotted onto nitrocellulose. Purifications were carried out for 48 h at 4°C, with fresh peptide/nitrocellulose added after 24 h. For P16*trans*, 400 µl antibody (in 1600 µl FA buffer) was incubated with 90 µg $P16_{OH}$*cis* and 90 µg unmodified H3, and purification performed exactly as for the P16*cis* antibody.

P16 isomerase assay

The proline isomerase assay was performed as described (Fischer et al., 1984). 10 µl of 7.8 mM paranitroaniline (pNA) peptide solution dissolved in water were used per 1 ml assay reaction. The unmodified, acetylated and substituted peptides, corresponding to residues 12-16 of histone H3 (GGKAP) followed by phenylalanine and the pNA group, were synthesised by Proteogenix, France and NeoBiolab, USA. The progress of the assay was monitored in real time every 0.6 s for 20 s in a spectrophotometer at 395 nm. The average initial rate of release of the pNA chromogenic substrate from the peptide after addition of chymotrypsin was calculated.

Chromatin immunoprecipitation

ChIP was performed as described in (Morillon et al., 2005). Briefly, cells grown to OD_{600} 0.5 in 50 ml of appropriate media were fixed with 1 % formaldehyde in 45 ml PBS for 30 min at

22°C followed by addition of glycine to 125 mM for 5 min. Cell pellets were collected by centrifugation (3000 rpm, 5 min) before washing twice with 10 ml cold PBS. Cells were resuspended in 500 µl cold FA-150 buffer (10 mM HEPES pH 7.9, 150 mM NaCl, 0.1 % SDS, 0.1 % sodium deoxycholate, 1 % Triton X-100) and broken using 1 ml glass beads on a MagnaLyser (Roche) at 4°C. Sample volume was increased to 2 ml with FA-150 buffer before shearing of the fixed chromatin by sonication using a biorupter (Diagenode, 30 min, 1 min on, 20s off, medium setting). Chromatin was cleared by centrifugation (10 000 rpm, 15 min, 4°C) and incubated with antibody (amounts stated in the Table above) in 1.5 ml siliconised Eppendorf tubes for 15-20 h rotating at 4°C. Bound chromatin was immunoprecipitated for 90 min at 22°C with 50 µl protein A-Sepharose pre-blocked with sonicated salmon sperm DNA. Beads and attached chromatin were pelleted by centrifugation (2600 rpm, 1 min) and washed with TSE-150 buffer (20 mM Tris-Cl pH 8.0, 150 mM NaCl, 2 mM EDTA, 0.1 % SDS, 1 % Triton X-100) for 3 min, TSE-500 buffer (20 mM Tris-Cl pH 8.0, 500 mM NaCl, 2 mM EDTA, 0.1 % SDS, 1 % Triton X-100) for 3 min, LiCl buffer (0.25 M LiCl, 10 mM Tris-Cl pH 8.0, 1 mM EDTA, 1 % dioxycholate, 1 % NP-40) for 15 min and twice with TE, all at 22°C. After washing, chromatin was eluted from the beads for 30 min at 65°C with elution buffer (0.1 M NaHCO3, 1 % SDS). Addition of 350 mM NaCl and incubation for 3 h at 65°C reversed the cross-links before treatment of samples with RNase A for 1 h at 37°C and proteinase K overnight at 65°C. DNA was purified using a PCR-purification kit (Qiagen) and eluted in 400 µl 1 mM Tris-Cl pH 8.0. Input DNA was diluted accordingly. Real-time quantitative PCR (qPCR) was carried out using a Corbett Rotorgene and Sybr green mix (Bioline). Data ([IP - no antibody control]/input) were expressed as a percentage of the input (relative to WT) and normalised to levels of H3 where appropriate. The primers used are listed in the table below (unless otherwise stated, the *FMP27* 5' primer used was *FMP27* primer 2).

Primer	Forward sequence	Reverse sequence
ADH1 1	CGGTATACGGCCTTCCTTCC	GGGAACGAGAACAATGACGA
ADH1 2	GCTATACCAAGCATACAATC	GGCTTTGGAACTGGAATATC
ADH1 3	GCCACTGACGGTGGTGCTCA	GGCACCAGCTGGCATACCGA
FMP27 1	TCTTGGTGGTGTTGCAGTTG	GCTGTTTGCTTTTACTGTCC
FMP27 2	CCGTCAGGCTAAAATCCGTT	CCTCTCTTGGTAATTCTTTA
FMP27 3	GGGTAACAATCTTATGGAAG	CCTTAATATTGTATGCTCGT
FMP27 4	GCCAAATAATCGCACTCCCA	GCGTTGGAGATTCTACCATC
FMP27 5	GGGATTTTATGATAACAGAG	GGGCACGAACAACGAGTAAT
FMP27 6	CGGAGCCTGAAGAACTTCGT	GGTCTGAAACATGGTAACA
PDC1	GTTTGCCAGGTGACTTCAAC	ACCGAAGGTGGTGATGATAC
PGK1	GCGTGTCTTCATCAGAGTTG	AGTGAGAAGCCAAGACAACG
RPL10	GAGCTGTTCCAGACTCCAAG	GTTGGCACAGATACGAGCAG
RPS15	ACGACCGATCATGTCTCAAG	TCTTCTAACTCTAGCTGGGG
SEN1	CCCAATAGCGATGTTCAGCT	CCCCTAATAATTTTGCCTCT

ChIP-sequencing and data analysis

Chromatin was immunoprecipitated essentially as described above except, where necessary, multiple immunoprecipitation reactions were performed and then pooled to obtain sufficient material for sequencing (10 ng). Immunoprecipitated DNA was quantified using Qubit Fluorometric Quantitation (Invitrogen). Sequencing was performed by the Wellcome Trust Centre for Human Genetics, Oxford. DNA was multiplexed during library preparation and subjected to 50 nt paired end single lane sequencing. Reads were aligned to the *S. cerevisiae* genome using Bowtie, allowing for up to two mismatches per read. Further analysis was performed using MATLAB (MathWorks). K4me3 was normalised to levels of histone H3 in each strain. To account for global changes in K4me3, the total read counts for K4me3/H3 in each strain were then normalised to the ratio of K4me3/H3 in the H3 substitution strain relative to the WT strain, as determined by immunoblotting (Zhang et al. 2011). Genome-wide H3-normalised K4me3 WT data was compared to other published datasets (Pokholok et al. (2005), ArrayExpress # E-WMIT-3, Kirmizis et al. (2007), GEO #

GSE8626) by dividing genes into 30 bins from the transcription start site (TSS) to the transcription termination site (TTS) (gene coordinates obtained from (Nagalakshmi et al., 2008), n= 6575) and calculating the average proportion of K4me3 as a fraction of the total K4me3 over the gene body. The most K14/P16-independent and K14/P16-dependent genes (n=200 for each of the four classes) were obtained by computing the average ratio of H3-normalised K4me3 in the H3 substitution strains over WT strain across the body of each non-dubious gene (TSS-TTS, n=5771). Subsequently, genes were ranked by the resultant ratio, whereby genes with the largest ratios were ranked at the top (rank =1). The K14/P16-independent genes are those with the highest 200 ratios of K4me3 (H3 substitution/WT) whereas the K14/P16-dependent genes are those with the lowest 200 ratios of K4me3. To produce the TSS-centred distribution plots, the ratios of H3-normalised K4me3 and log2 levels of Nrd1 (ChIP-seq data from (Mayer et al., 2012), ArrayExpress # E-MTAB-1060) were calculated for each gene and strain from 1500 bp upstream to 1500 bp downstream of the TSS and median ratios of K4me3/H3 determined for all genes and the 200 most K14/P16-independent and most K14/P16-dependent genes in both the K14A and P16V strains. Levels of nascent sense and antisense transcription (NET-seq data from (Churchman and Weissman, 2011), GEO # GSE25107) per base pair were calculated for each gene in a 300 bp window downstream of the TSS and plotted as a boxplot for all genes and the four described gene classes. Gene ontology analysis was performed using the DAVID functional annotation tool v 6.7 (Huang da et al., 2009a,b). P-values for the overlap analysis between the 200 most K14/P16-independent and K14/P16-dependent genes and genes regulated during the yeast metabolic cycle, environmental stress response or by SAGA/TFIID, or classed as RPG/Ribi were calculated from a simulated probability distribution curve.

Purification of histones from *S. cerevisiae* for mass spectrometry

Histones were prepared essentially as (Edmondson et al., 1996). Yeast cells were grown in 300 ml YPD to OD_{600} 0.5-0.8. Cells were harvested (5000 rpm JA-10 rotor, 5 min, 4°C) and washed in sterile water. Cells were resuspended in 0.1 mM Tris-Cl pH 9.4, 10 mM DTT and incubated for 15 min at 30°C with gentle shaking. Following a wash with and then resuspension in 1.2 M sorbitol, 20 mM HEPES pH 7.4, cells were spheroplasted with 2.75 g zymolyase/g yeast cells for 60 min at 30°C with gentle shaking. A series of washes was then performed in: ice-cold 1.2 M sorbitol, 20 mM PIPES pH 6.8, 1 mM $MgCl_2$; ice-cold nuclear isolation buffer (0.25 M sucrose, 60 mM KCl, 15 mM NaCl, 5 mM $MgCl_2$, $CaCl_2$, 15 mM MES pH 6.6, 0.8 % Triton X-100, protease inhibitors) twice with incubations on ice for 20 min; buffer A (10 mM Tris-Cl pH 8.0, 0.5 % NP-40, 75 mM NaCl, 30 mM sodium butyrate, protease inhibitors) twice with 15 min incubations on ice; and buffer B (10 mM Tris-Cl pH 8.0, 0.4 M NaCl, 30 mM sodium butyrate, protease inhibitors) twice with 10 min incubation on ice for the first wash. The resulting nuclei were resuspended in cold 0.4 N H_2SO_4 and incubated on ice for 30 min. After centrifugation (13 000 rpm, 10 min, 4°C), the supernatant (containing histones) was added to 5 volumes of acetone. Proteins were precipitated overnight at -20°C. Precipitated protein was collected by centrifugation (13 000 rpm, 10 min, 4°C) and air-dried before resuspension in 10 mM Tris-Cl pH 8.0. Samples were separated using 18 % SDS-polyacrylamide gel electrophoresis before excision of an appropriate molecular weight gel band and submission for mass spectrometry (Central Proteomics Facility, Dunn School of Pathology, Oxford).

Identification of histone H3 post-translational modifications by MS/MS

Histones were prepared for mass spectrometry essentially as described (Edmondson et al., 1996) and excised after 18 % SDS-polyacrylamide gel electrophoresis. The excised gel band was digested with trypsin overnight at 37°C. The resulting peptides were then analysed on an Ultimate 3000 nano HPLC system (Dionex, Camberley, UK) run in direct injection mode coupled to a LTQ XL Orbitrap mass spectrometer (Thermo Electron, Hemel Hempstead, UK; Central Proteomics Facility, Dunn School of Pathology, Oxford. Samples were resolved on a 15 cm x 75 µm inner diameter PicoTip analytical column (New Objective, Woburn, MA, USA), which was packed in-house with Reprosil-Pur C18-AQ phase, 3 µm bead (Dr. Maisch, Germany). A 120 min gradient was used to separate the peptides. The mass spectrometer was operated in a “Top 5” data-dependent acquisition mode. Precursor scans were performed in the Orbitrap at a resolving power of 60,000, from which five precursor ions were selected and fragmented in the linear ion trap. Charge state +1 ions were rejected. Data analysis was performed using Mascot v. 2.2 against a custom database containing the NCBI *S. cerevisiae* protein database and the substituted (P16V) histone H3 protein sequence. The search was performed with fixed carbamidomethyl modification and variable oxidation, acetyl(K), methyl(K), dimethyl(K) and trimethyl(K) modifications. The peptide mass tolerance (± 20 ppm), the fragment mass tolerance (± 0.5 Da) and the maximum number of missed cleavages (2) were defined in the Mascot search parameters. Peak intensities for the peptides containing K14ac in the WT and P16V samples were then calculated and compared. Peak intensities for the peptides containing K9ac and K23ac, which remain unchanged between the WT and P16V strains (as assessed by Western blotting), were used to control for loading and differences in ionisation caused by the P16V substitution.

Peptides for bromodomain interaction studies

Peptides were synthesized at GL Biochem (Shanghai) LTD, China and Proteogenix, France. Purity was determined with MALDI-TOF mass spectrometry and HPLC and the peptides used in these experiments had a minimum purity of 84 %. The peptide sequences are listed in the table below.

**Peptide**	**Sequence**	**MW (Da)**
Unmodified	TGGKAPRKQLASKAARC	1743.07
K18ac	TGGKAPR(Kac)QLASKAARC	1785.11
P16V-K18ac	TGGKAVR(Kac)QLASKAARC	1787.12
K14R-K18ac	TGGRAPR(Kac)QLASKAARC	1812.12
K14A-K18ac	TGGAAPR(Kac)QLASKAARC	1728.01
K14R-P16V-18ac	TGGRAVR(Kac)QLASKAARC	1815.14
K14ac	TGG(Kac)APRKQLASKAARC	1785.11
K14ac-K18ac	TGG(Kac)APR(Kac)QLASKAARC	1827.14

Spt7 bromodomain expression and purification

Spt7 bromodomain (amino acids 363-619) was cloned by PCR from genomic yeast DNA and then sub-cloned into expression vector pIVEX2.3d. The predicted bromodomain binding site residues Y500, Y520 and N521 were mutated to alanine by site-directed mutagenesis (QuickChange II) according to manufacturer's instructions (Agilent Technologies, UK). Proteins were expressed in *E. coli* BL21 T7 I^q LysY cells (NEB, UK) and purified on 1 ml HisTrap columns (GE Healthcare, UK) using a step-wise gradient of imidazole (40-500 mM) according to conditions described previously (Boubriak et al., 2009). Proteins were further purified by gel filtration on Superdex 75 column in 10 mM Hepes, 150 mM NaCl, 2 mM EDTA and 1 mM TCEP buffer and appropriate fractions concentrated on Vivaspin 6 devices.

Identity and purity of the protein was confirmed by N-terminal sequencing (AltaBiosciences, UK) and TOF MS ES+ mass spectrometry, and the molecular weight for the intact bromodomain protein was 31.1 kDa, as predicted.

Peptide pulldowns

SulfoLink Coupling Resin from Thermo Scientific, UK (Cat 20401) was used in all experiments. Peptides were coupled to the resin according to the manufacturer's instructions with some modification. Briefly, 2 ml of resin slurry per sample was prepared in 15 ml Falcon tubes. 2 ml of 0.5 mg/ml peptide in coupling buffer (50 mM Tris-Cl, 5 mM EDTA, pH 8.5) with 25 mM TCEP was immobilised to resin during 15 min rotation, followed by 30 min stand. After washing twice with coupling buffer, uncoupled sites where blocked by addition of 2 ml of freshly prepared 50 mM L-Cysteine-HCL solution. Resin pellet was further re-suspended and washed in washing buffer (1 M NaCl) and stored in PBS. Before use coupled resin was resuspended in binding buffer (40 mM HEPES, 175 mM NaCl, 0.1 % Tween 20, 10 % glycerol) and blocked with BSA (5 mg/ml). In each experiment 200 µl of resin was incubated with 350 µl of bromodomain (diluted with binding buffer to final concentration of 30 µg/ml) for 2 h at 4°C. Unbound protein was collected from the beads and after multiple washes with binding buffer, high salt (350 mM NaCl) and TE buffer, bound material was eluted by heating at 80°C with 120 µl LDS (Invitrogen)

SPR interaction studies

SPR data for peptides binding to immobilized Spt7 bromodomain was generated on Biacore T100 and T200 instruments at 25°C. In all cases S NTA chips were used to immobilise C-terminal His-tagged bromodomain via Ni^{2+} capture of the affinity tag. Typically bromodomain in 10 mM HEPES, 150 mM NaCl buffer at pH 7.4 was injected at 60 µg/ml resulting in 1.0 K RU – 4K RU of protein immobilised. After priming of the flow cells of the NTA chip, the binding of peptides dissolved in HBS-P+ running buffer (GE Healthcare, UK) in the concentration range from 1-200 µM was measured. For regeneration of the chip surface in addition to the standard 350 µM EDTA solution, 1 M imidazole solution was used. Sensorgrams, binding curves and K_d values were analysed with BIA T100 and T200 evaluation software (GE Healthcare, UK) using a 1:1 binding model.

Supplemental references

Boubriak, I., Mason, P.A., Clancy, D.J., Dockray, J., Saunders, R.D., and Cox, L.S. (2009). DmWRNexo is a 3'-5' exonuclease: phenotypic and biochemical characterization of mutants of the Drosophila orthologue of human WRN exonuclease. Biogerontology *10*, 267-277.

Churchman, L.S., and Weissman, J.S. (2011). Nascent transcript sequencing visualizes transcription at nucleotide resolution. Nature *469*, 368-373.

Dai, J., Hyland, E.M., Yuan, D.S., Huang, H., Bader, J.S., and Boeke, J.D. (2008). Probing nucleosome function: a highly versatile library of synthetic histone H3 and H4 mutants. Cell *134*, 1066-1078.

Edmondson, D.G., Smith, M.M., and Roth, S.Y. (1996). Repression domain of the yeast global repressor Tup1 interacts directly with histones H3 and H4. Genes Dev *10*, 1247-1259.

Gasch, A.P., Spellman, P.T., Kao, C.M., Carmel-Harel, O., Eisen, M.B., Storz, G., and Botstein, D. (2000). Genomic expression programs in the response of yeast cells to environmental changes. Mol Biol Cell *11*, 4241-4257.

Huang da, W., Sherman, B.T., and Lempicki, R.A. (2009a). Bioinformatics enrichment tools: paths toward the comprehensive functional analysis of large gene lists. Nucleic Acids Res *37*, 1-13.

Huang da, W., Sherman, B.T., and Lempicki, R.A. (2009b). Systematic and integrative analysis of large gene lists using DAVID bioinformatics resources. Nat Protoc *4*, 44-57.

Huisinga, K.L., and Pugh, B.F. (2004). A genome-wide housekeeping role for TFIID and a highly regulated stress-related role for SAGA in Saccharomyces cerevisiae. Mol Cell *13*, 573-585.

Kirmizis, A., Santos-Rosa, H., Penkett, C.J., Singer, M.A., Vermeulen, M., Mann, M., Bahler, J., Green, R.D., and Kouzarides, T. (2007). Arginine methylation at histone H3R2 controls deposition of H3K4 trimethylation. Nature *449*, 928-932.
Longtine, M.S., McKenzie, A., Demarini, D.J., Shah, N.G., Wach, A., Brachat, A., Philippsen, P., and Pringle, J.R. (1998). Additional modules for versatile and economical PCR-based gene deletion and modification in *Saccharomyces cerevisiae*. Yeast *14*, 953-961.
Mann, R.K., and Grunstein, M. (1992). Histone H3 N-terminal mutations allow hyperactivation of the yeast GAL1 gene in vivo. Embo J *11*, 3297-3306.
Mayer, A., Heidemann, M., Lidschreiber, M., Schreieck, A., Sun, M., Hintermair, C., Kremmer, E., Eick, D., and Cramer, P. (2012). CTD tyrosine phosphorylation impairs termination factor recruitment to RNA polymerase II. Science *336*, 1723-1725.
Morillon, A., Karabetsou, N., Nair, A., and Mellor, J. (2005). Dynamic lysine methylation on histone H3 defines the regulatory phase of gene transcription. Mol Cell *18*, 723-734.
Nagalakshmi, U., Wang, Z., Waern, K., Shou, C., Raha, D., Gerstein, M., and Snyder, M. (2008). The transcriptional landscape of the yeast genome defined by RNA sequencing. Science *320*, 1344-1349.
Nakanishi, S., Sanderson, B.W., Delventhal, K.M., Bradford, W.D., Staehling-Hampton, K., and Shilatifard, A. (2008). A comprehensive library of histone mutants identifies nucleosomal residues required for H3K4 methylation. Nat Struct Mol Biol *15*, 881-888.
Pokholok, D.K., Harbison, C.T., Levine, S., Cole, M., Hannett, N.M., Lee, T.I., Bell, G.W., Walker, K., Rolfe, P.A., Herbolsheimer, E*., et al.* (2005). Genome-wide map of nucleosome acetylation and methylation in yeast. Cell *122*, 517-527.
Tu, B.P., Kudlicki, A., Rowicka, M., and McKnight, S.L. (2005). Logic of the yeast metabolic cycle: temporal compartmentalization of cellular processes. Science *310*, 1152-1158.
Zhang, L., Ma, H., and Pugh, B.F. (2011). Stable and dynamic nucleosome states during a meiotic developmental process. Genome Res *21*, 875-884.
Zhang, W., Bone, J.R., Edmondson, D.G., Turner, B.M., and Roth, S.Y. (1998). Essential and redundant functions of histone acetylation revealed by mutation of target lysines and loss of the Gcn5p acetyltransferase. Embo J *17*, 3155-3167.
