# Supplementary material for: Lysine Acetylation Controls Local Protein Conformation by Influencing Proline Isomerization
Source: Mol Cell. 2014 Sep 4;55(5):733–44. doi: 10.1016/j.molcel.2014.07.004 (PMC4157579; doi:10.1016/j.molcel.2014.07.004)
Supplement: Document S2. Article plus Supplemental Information [file mmc3.pdf]

Molecular Cell
Article

CellPress

Lysine Acetylation Controls Local Protein Conformation by Influencing Proline Isomerization

Françoise S. Howe,[1] Ivan Boubriak,[1] Matthew J. Sale,[1,2] Anitha Nair,[1] David Clynes,[1,3] Anne Grijzenhout,[1] Struan C. Murray,[1] Ronja Woloszczuk,[1] and Jane Mellor[1,*]
[1]Department of Biochemistry, University of Oxford, South Parks Road, Oxford OX1 3QU, UK
[2]Present address: Signalling Laboratory, The Babraham Institute, Babraham Research Campus, Cambridge CB22 3AT, UK
[3]Present address: The Weatherall Institute of Molecular Medicine, John Radcliffe Hospital, Headington, Oxford OX3 9DS, UK
*Correspondence: jane.mellor@bioch.ox.ac.uk
http://dx.doi.org/10.1016/j.molcel.2014.07.004
This is an open access article under the CC BY license (http://creativecommons.org/licenses/by/3.0/).

SUMMARY

Gene transcription responds to stress and metabolic signals to optimize growth and survival. Histone H3 (H3) lysine 4 trimethylation (K4me3) facilitates state changes, but how levels are coordinated with the environment is unclear. Here, we show that isomerization of H3 at the alanine 15-proline 16 (A15-P16) peptide bond is influenced by lysine 14 (K14) and controls gene-specific K4me3 by balancing the actions of Jhd2, the K4me3 demethylase, and Spp1, a subunit of the Set1 K4 methyltransferase complex. Acetylation at K14 favors the A15-P16*trans* conformation and reduces K4me3. Environmental stress-induced genes are most sensitive to the changes at K14 influencing H3 tail conformation and K4me3. By contrast, ribosomal protein genes maintain K4me3, required for their repression during stress, independently of Spp1, K14, and P16. Thus, the plasticity in control of K4me3, via signaling to K14 and isomerization at P16, informs distinct gene regulatory mechanisms and processes involving K4me3.

INTRODUCTION

The packaging of eukaryotic genomes into chromatin has fundamental effects on gene expression but how this is brought about still remains poorly understood. Histone proteins are highly conserved and are subject to many different posttranslational modifications (PTMs) including acetylation and methylation (Rando and Winston, 2012). These modifications can influence nucleosome occupancy and position as well as the recruitment of a wide range of effector proteins implicated in a variety of cellular processes. Histone methylation can be influenced by the conformation of the N-terminal region of histone H3, particularly *cis-trans* isomerization of bonds around proline residues (Lu et al., 2007; Nelson et al., 2006; Youdell et al., 2008). The peptidyl-prolyl isomerase (PPIase) Fpr4 increases the rate of *cis-trans* isomerization at all three prolines (16, 30, and 38) on H3 in vitro (Monneau et al., 2013) and H3K37-P38 isomerization influences Set2-mediated H3K36me3 (Nelson et al., 2006).

Genome-wide mapping studies show that modification patterns are correlated with both gene structure and gene activity, often showing characteristic distributions on active or repressed genes (Liu et al., 2005; Pokholok et al., 2005). One such modification is Set1-dependent methylation of lysine 4 on histone H3 (K4), present in most eukaryotes at active or potentially active genes (Santos-Rosa et al., 2002). This has led some to assume that K4 methylation by Set1 is an activating modification yet, in yeast, most evidence points to K4 methylation having a repressive or adaptive role, silencing some rDNA repeats (Briggs et al., 2001; Bryk et al., 2002), repressing genes in mid-sporulation and during exponential growth (Carvin and Kladde, 2004; Fingerman et al., 2005; Guillemette et al., 2011; Lenstra et al., 2011; Wang et al., 2011), and facilitating the transcriptional response to diamide stress (Weiner et al., 2012). Paradoxically, but consistent with a modulatory role, K4 methylation facilitates the recruitment of both lysine acetyl transferases (KATs) and histone deacetylases (HDACs) (Vermeulen and Timmers, 2010). Set1 is found in a complex (Set1C) with seven other subunits (Swd1, Swd3, Bre2, Sdc1, Spp1, Swd2, and Shg1) (Briggs et al., 2001; Nagy et al., 2002; Roguev et al., 2003) that contribute to complex integrity and/or degree of methylation (K4me1, K4me2 or K4me3). Spp1 is required for K4me3 (Morillon et al., 2005) and contains a PHD motif that interacts with K4me2 and K4me3 (Murton et al., 2010; Shi et al., 2007) thus maintaining high local levels of K4me3. Loss of K4me3 from chromatin involves either histone dilution during DNA replication (Radman-Livaja et al., 2010) or active demethylation by Jhd2 (Ingvarsdottir et al., 2007; Liang et al., 2007; Seward et al., 2007; Tu et al., 2007). Jhd2 is particularly important in sporulation to keep certain genes expressed (Xu et al., 2012). Methylated K4 is influenced by the modification state at distant residues on nucleosomal histones including H2Bub1 and K14ac promoting, and H3R2me and K4ac antagonizing K4me2 and K4me3 to varying extents (Briggs et al., 2002; Guillemette et al., 2011; Kirmizis et al., 2007; Maltby et al., 2012; Nakanishi et al., 2008).

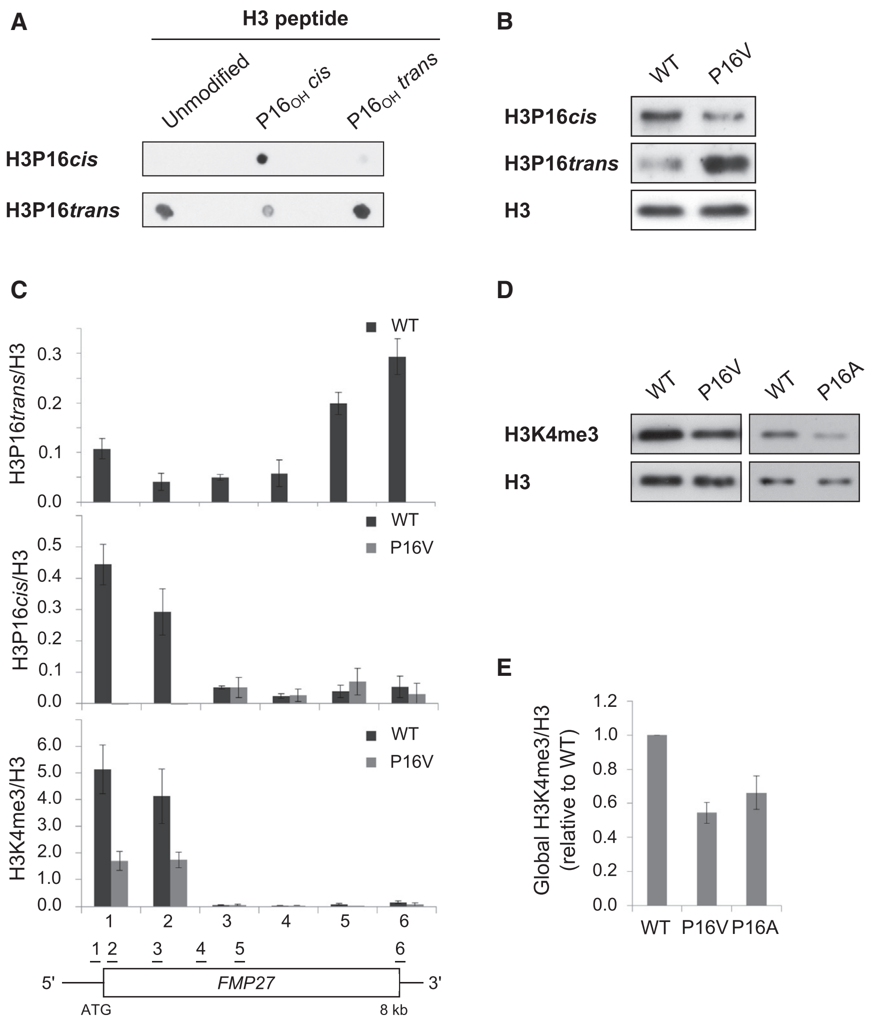

Figure 1. Proline 16 Adopts Two Conformations to Influence K4me3

(A) Unmodified, *cis*-hydroxyproline and *trans*-hydroxyproline peptides (0.1 µg) were dotted onto nitrocellulose and incubated as for western blotting with P16*cis* or P16*trans* antibody purified for 48 hr before incubation.

(B) Western blot analysis of purified P16*cis* and P16*trans* antibody binding to WT and P16V whole cell protein extracts.

(C) Chromatin immunoprecipitation (ChIP)-qPCR showing the levels of P16*cis*, K4me3, and P16*trans* at *FMP27*, normalized to levels of H3 at positions indicated, ± SEM for three repeats.

(D) Western blots showing the reduction in global levels of K4me3 in the P16V and P16A strains relative to WT.

(E) Averaged quantitation ± SEM for H3-normalized K4me3 (n = 4–9).

See also Figure S1.

Cells alternate between phases of growth and quiescence, known as the yeast metabolic cycle (YMC) (Tu et al., 2005). Global levels of K4me3 remain fairly constant but levels of H3 acetylation vary, for example on K14 (Cai et al., 2011; Tu et al., 2005). This may reflect fluctuations in the levels of acetyl CoA, a cofactor for KATs, which are lower in quiescent cells. Quiescent cells are more resistant to stress (Lu et al., 2009; Slavov et al., 2012) and express genes that negatively correlate with growth rate (Brauer et al., 2008) and genes whose expression increases during the common environmental stress response (ESR) (Gasch et al., 2000; O'Duibhir et al., 2014). Feeding quiescent cells metabolic intermediates, such as acetate, induces cycling and growth (Cai et al., 2011; Shi and Tu, 2013). Moreover, the lysine acetyltransferase, Gcn5 (KAT2) a component of SAGA (Baker and Grant, 2007), is required for metabolic cycling (Cai et al., 2011). This suggests that acetylation on histone H3 will reflect the metabolic state of the cell and environmental growth-related signals.

As K14 acetylation is reported to be required for K4me3 (Maltby et al., 2012; Nakanishi et al., 2008) and K14 is subject to Gcn5/SAGA-dependent acetylation (Jiang et al., 2007; Zhang et al., 1998), we wanted to explore in more detail the relationship between K14 and K4me3. Here, we show that K14 modulates the conformation of the H3 tail at the alanine 15-proline 16 peptide bond (*cis-trans* isomerization) to control K4me3. Substitutions at K14 (A, R, and Q) and P16 (V and A) to mimic neutral, positively charged, acetylated or *trans* conformation of the H3 tail respectively reveal the different contributions of these residues in promoting Spp1 association with chromatin and antagonizing Jhd2-mediated demethylation of K4me3. K14ac is associated with A15-P16*trans* and reduced K4me3. Strains lacking Spp1 show a similar reduction (≈5-fold decrease) in global K4me3 levels to the K14A strain. This residual level of K4me3 is due to a Spp1-, K14-, and P16-independent mechanism for deposition of K4me3, most evident at the ribosomal protein genes (RPGs). By contrast, environmental stress response (ESR)-induced genes are most dependent on Spp1, K14, and P16 for K4me3. We propose that changes in growth conditions and the availability of metabolic intermediates and regulators allow variable control over levels of K4me3 at different genic loci.

RESULTS

Proline is unique in its ability to form *cis* or *trans* peptide bonds with the preceding residue. We were interested in whether, as at P38 (Nelson et al., 2006), *cis-trans* isomerization of P16 is important for H3 tail modifications. For evidence that P16 adopts *cis* or *trans* conformations in vivo, we raised antibodies against short H3 peptides in which the A15-P16 peptidyl-prolyl bond was fixed predominantly in the *cis* or *trans* configuration by hydroxylation of the proline ring (Taylor et al., 2005). After extensive selection for specificity using the peptides (Figure 1A), we obtained antibodies that specifically recognized the peptide with

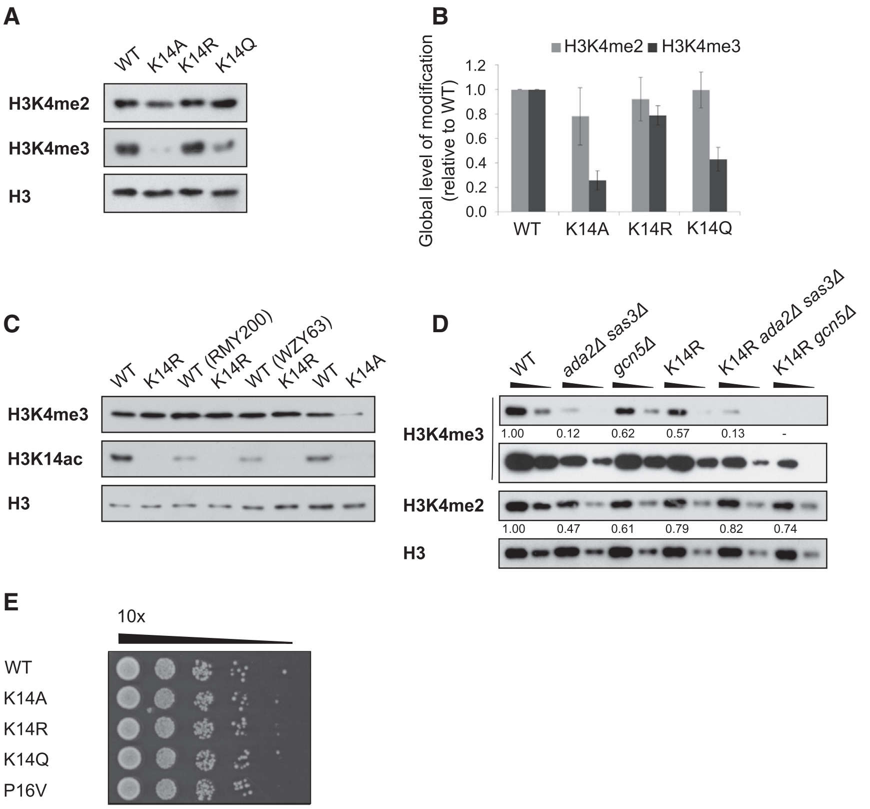

Figure 2. Substitutions at K14 Affect Levels of K4me3

(A) Western blots showing the levels of H3, K4me2, and K4me3 in the WT and K14 substitution strains.

(B) Western blot quantitation of H3-normalized K4me2 (n = 4) and K4me3 (n = 9) displayed as mean signals ± SEM.

(C) Western blot showing levels of K4me3 in WT and K14R strains from three different yeast backgrounds. K14ac levels control for the K14 mutation.

(D) Western blot showing the levels of K4me2 and K4me3 (two exposures) in the *ada2/sas3* and *gcn5* deletion strains. H3-normalized K4 methylation signals are quantified relative to WT.

(E) Drop plate growth assay (2 days at 30°C) showing 10-fold serial dilutions on YPD-agar.

hydroxylated P16 in the desired conformation (the unmodified peptide will have the majority of P16 in *trans*). We further characterized these antibodies by western blot using a WT strain (both *cis* and *trans* conformations) and a strain with P16 substituted for valine, which fixes the A15-V16 bond in *trans* (Lu et al., 2007; Nelson et al., 2006) (Figure 1B). As expected, we observed a higher P16*cis* signal in the WT over the P16V strain but a higher P16*trans* signal in the P16V strain relative to the WT. The antibodies were used to perform a chromatin immunoprecipitation (ChIP) experiment to detect A15-P16 in *cis* and in *trans* in the chromatin over the long gene *FMP27* (Figure 1C and Figure S1A available online; *ADH1*). P16*trans* is low at the 5′ region of *FMP27* and increases toward the 3′ end of the gene. Conversely, we observed P16*cis* at the 5′ region of the genes, with a similar distribution to that we had previously observed for K4me3. We asked whether the integrity of P16 is required for K4me3. Both valine and alanine substitutions at P16 significantly reduced global levels of K4me3 (Figures 1D and 1E). We tested other modifications on H3 but none, apart from K4me3, K14ac, and K18ac, were influenced by the P16 substitution (Figure S1B). Dot blot analysis demonstrated that antibodies raised against K14ac and K18ac were unable to bind peptides containing their epitope and a P16V substitution (Figure S1C), but mass spectrometry revealed the level of K14ac in a P16V strain was roughly equivalent to WT (Figure S1D). This suggests that the antibodies specific for K14ac and K18ac require P16 as part of their epitope. Thus P16 adopts distinct conformations in vivo and is required for optimal levels of K4me3.

A, Q, and R Substitution at K14 Differently Affect K4me3

It has been reported that substitutions at K14 also result in reduced K4me3 but not K4me1/2 (Nakanishi et al., 2008). Given the effect of P16 substitution on K4me3, we wanted to reexplore the relationship between K14 and K4me3. As described previously, substitution of K14 with alanine (A) or glutamine (Q) causes reductions in the global level of K4me3. In contrast, substitution of K14 with arginine (R) resulted in less severe reductions in global K4me3 (Figures 2A and 2B), confirmed in a range of strain backgrounds (Figure 2C). Lysines for which K to R and K to Q substitutions have opposite effects are known acetylation substrates, such as K14 (Weiner et al., 2012). As there are robust levels of K4me3 in the K14R strain, a lysine at position 14 is not absolutely required for K4me3 and this also rules out a strict dependency on acetylation of K14 for K4me3. Moreover, as the arginine substitution was used to mimic the positively charged, unmodified lysine residue whereas glutamine is a mimic of the neutral, acetylated lysine (Wang and Hayes, 2008), this suggests that unmodified K14 is associated with K4me3.

Acetylation of K14 Is Not Directly Linked to Trimethylation of Lysine 4

We suggest that K14 in its unmodified state is associated with K4me3, whereas three different groups have concluded, using strains lacking acetyltransferases such as Gcn5 or Sas3, that acetylation is required for K4me3 (Govind et al., 2007; Jiang et al., 2007; Maltby et al., 2012). As these strains have a significantly reduced growth rate (Zhang et al., 1998), it is possible that the reduction in K4me3 is not a direct consequence of reduced K14ac. To test this, *GCN5* or *SAS3* with *ADA2* (Ada2 is required for the KAT activity of Gcn5), were deleted in the K14R strain and a strain with WT histone H3. We note less change in K4me2 levels. Deletion of *GCN5*, or *SAS3* with *ADA2*, reduced the global level of K4me3 in the presence of a WT copy of histone H3, as reported previously. However, deletion of *GCN5* or *SAS3* with *ADA2* in the K14R strain further reduced the level of K4me3

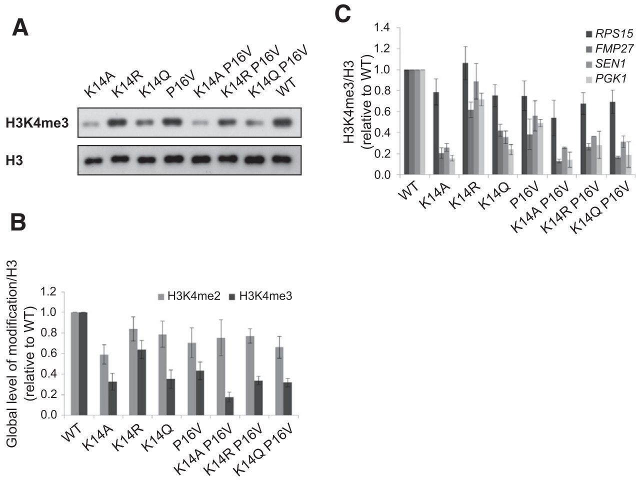

Figure 3. K14 and P16 Have Distinct but Overlapping Effects on K4me3

(A) Western blot showing levels of K4me3 in the WT, K14/P16 single and double substitution strains.

(B) H3-normalized K4me2 and K4me3 signals are quantified ± SEM relative to those of the WT strain (n = 4).

(C) Averaged ± SEM ChIP-qPCR experiments showing the levels of H3-normalized K4me3 at the 5′ end of four genes (*RPS15*, *SEN1*, *FMP27*, and *PGK1*) in the K14/P16 substitution strains relative to the WT strain (n = 2–8).

See also Figure S2.

when compared to the control K14R strain (Figure 2D). Because there is already no K14ac in the K14R strain, the additional reduction in K4me3 cannot be due to loss of this modification. Therefore, Gcn5 and Sas3/Ada2 must be able to modulate K4me3 independently of K14ac, possibly via acetylation of other lysines, global changes in transcription or indirectly through the reduced growth rate of the deletion strains. Many gene deletions cause slow growth and result in a transcriptional signature that correlates strongly with the common environmental stress response (ESR) (O'Duibhir et al., 2014). However, there was no obvious difference in growth rate or stress resistance of the K14 and P16 substitution strains compared to WT that might explain reduced K4me3 (Figure 2E). Together, the varying levels of K4me3 in the P16 and the three K14 substitution strains point toward a more modulatory role for these residues rather than a strict requirement for acetylation at K14.

Distinct Functions for K14 and P16 in Regulating K4me3

To investigate if K14 and P16 were influencing K4me3 through a common pathway, K14 P16 double substitution strains were created (no growth defect observed, Figure S2A) and levels of K4me3 were assessed globally or by ChIP at the 5′ ends of *RPS15*, *SEN1*, *FMP27*, and *PGK1* (Figure 3). The global levels (Figures 3A and 3B) and the ChIP signals (Figure 3C) for K4me3 in the K14A P16V and K14Q P16V strains were similar to the K14A and K14Q single substitutions, respectively. By contrast, the signals in the K14R P16V strain were most reduced compared to the single K14R substitution. Together, these findings imply that there may not be a simple linear pathway between K14, P16, and K4me3, perhaps consistent with a structural role for P16 and an additional function at K14. We note that the relative K4me3 levels by ChIP varied at the four genes (Figure 3C), suggesting that genes respond nonuniformly to these substitutions.

Substitutions at K14 and P16 Differentially Affect the Association of Spp1-HA with Chromatin

Next, we wanted to uncover more about the mechanism by which the K14 and P16 substitutions modulate levels of K4me3. Because Spp1 is required for K4me3, we asked whether K14 and P16 substitutions influence the association of Spp1 with chromatin. ChIP of Spp1-HA at six genes in the four histone substitution strains revealed levels at or above WT in the K14R strain, reduced levels in the P16V strain and low levels in the K14A and K14Q substitution strains, generally reflecting global levels of K4me3 in these strains (Figure 4A). No change in global Spp1-HA protein levels, or Swd1-HA in the Set1C, is observed in the H3 substitutions (Figure S2B). This supports a role for K14 and P16 in Spp1-dependent K4me3. Once again, as the K14R substitution has near WT levels of both Spp1 chromatin association and global K4me3, acetylation of K14 cannot be required for Spp1 binding and K4me3.

Substitutions at K14 and P16 Influence the Balance of Spp1 and Jhd2 Activity to Determine Levels of K4me3

We asked whether Spp1 and substitutions at K14/P16 are epistatic by deleting *SPP1* from the K14/P16 substitution strains. Levels of K4me3 were further reduced in the K14/P16-substituted *spp1*Δ strains but remained in proportion to the amount of K4me3 in the individual K14/P16 substitutions when *SPP1* is present (Figures 4B and 4C). This implies that the H3 substitutions must additionally modulate K4me3 via a Spp1-independent pathway. Interestingly, levels of K4me2 are also substantially lower in the K14A *spp1*Δ and K14Q *spp1*Δ strains than in the K14R *spp1*Δ strain, suggesting that these K14 substitutions render the residual K4 methylation in the *spp1*Δ strain more sensitive to demethylation by Jhd2 (Figure 4B). To test this, *JHD2* was deleted in a variety of strains. First, we examined K4me3 in *jhd2*Δ strains with a WT copy of histone H3. An increase in K4me3 was observed upon *JHD2* deletion at both the global level (Figures 4C and 4D) and at the 5′ end of *FMP27* by ChIP (Figure 4E) in the presence or absence of Spp1. This suggests that the sensitivity to Jhd2 is independent of Spp1.

Next, we examined the effects of the *JHD2* deletion in the K14/P16-substituted strains. We note that levels of Jhd2 protein do not change in these strains (Figure S2C). Upon *JHD2* deletion, global levels of K4me3 increased in all histone substitutions (Figures 4C and 4D). To see whether any of the substitutions resulted in a greater increase in K4me3 in the absence of Jhd2 (*jhd2*Δ) compared to the increase in the WT histone H3 strain, we assessed the fold change in K4me3 (Figure 4F). The K14A, K14Q, and P16V strains all showed a larger fold change than observed in the WT or K14R strains, suggesting that these substitutions result in increased action of Jhd2 toward K4me3, accounting in part for the reduced levels compared to wild-type (WT).

Finally, we examined the effects of the double *spp1*Δ*jhd2*Δ deletions in the K14/P16-substituted strains (Figures 4B and 4C).

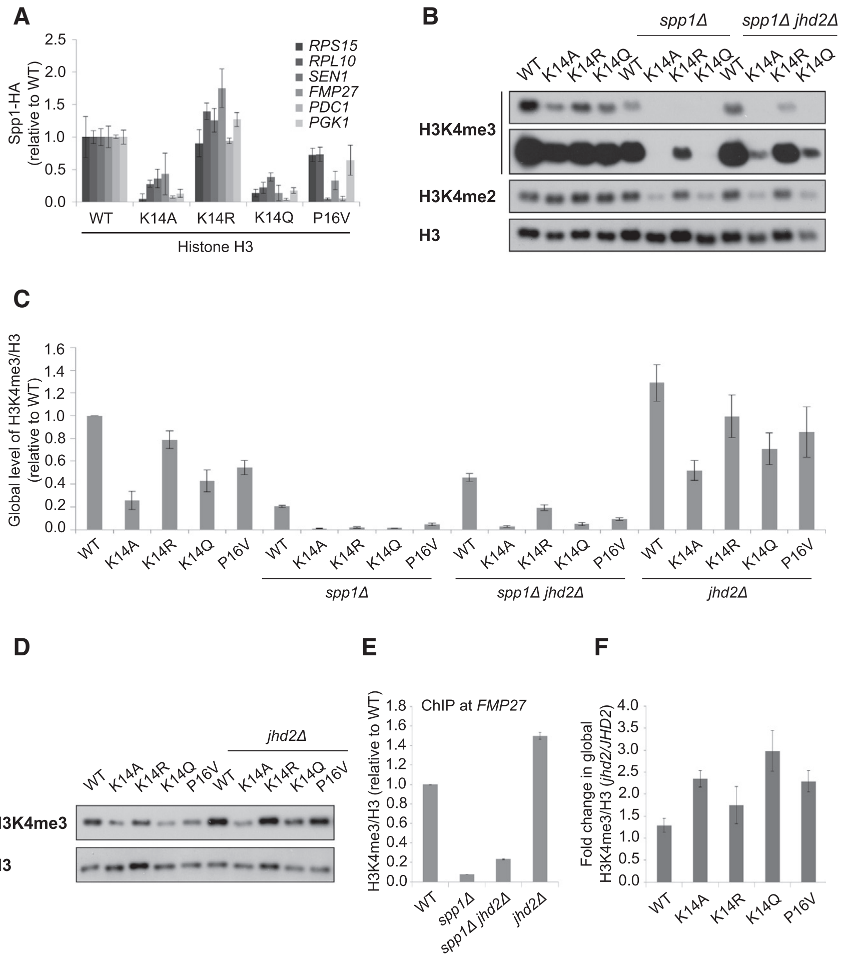

Figure 4. K14 and P16 Control the Balance of Spp1 and Jhd2 Action

(A) Levels of Spp1-HA on chromatin at the 5′ ends of *RPS15*, *RPL10*, *SEN1*, *FMP27*, *PGK1*, and *PDC1* in the strains indicated relative to WT. Error bars show SEM of the real-time PCR reaction and are representative of at least three independent experiments.

(B) Western blot showing levels of K4me2 and K4me3 (two exposures) in strains indicated.

(C) Average quantitation ± SEM (n = 2–4) of H3-normalized K4me3.

(D) Western blot showing levels of K4me3 in the K14/P16 substitution strains ± *JHD2*.

(E) Average levels ± SEM of H3-normalized K4me3 (n = 2) at the 5′ end of *FMP27* by ChIP-qPCR in strains indicated.

(F) Western blot quantitation shows the average ratios of K4me3 in the *jhd2Δ* relative to the *JHD2* strains in the WT histone H3 and K14/P16 substitution strains ± SEM (n = 3). All fold changes in K4me3 are displayed relative to the WT strain.

See also Figure S2.

Although levels are low, in all *spp1*Δ*jhd2*Δ strains we observed an increase in K4me3 compared to the *spp1*Δ K14/P16 substitutions. Interestingly, deletion of *JHD2* did not even partially rescue levels of K4me2 in the K14A *spp1*Δ or K14Q *spp1*Δ strains (Figure 4B). Therefore, reduced K4me2 in these strains cannot be explained by increased demethylation, but could instead result from increased histone turnover or from the K14A/Q substitutions compromising the capacity of the Set1C to both di- and trimethylate K4 in the absence of Spp1. Thus the H3 substitutions are altering the balance of K4 methylation by Set1/Spp1 and demethylation by Jhd2 to result in reduced levels of K4me3.

So far we have demonstrated how K14 and P16 are both required for optimal K4me3. These residues influence K4me3 in an overlapping but distinct manner with similar altering of the balance of Spp1 and Jhd2 action. Interestingly, K4me3 is reduced on some genes more than others by these substitutions. Thus, we addressed how changes to the H3 tail around K14 and P16 might influence K4me3 genome-wide.

K14 and P16 Differentially Regulate the Levels of K4me3 on Functionally Distinct Classes of Genes

To examine the effect of substitutions at K14 and P16 on levels of K4me3 genome-wide, ChIP-sequencing (ChIP-seq) experiments for K4me3 were performed with the WT, K14A, and P16V substitution strains, normalized to H3 levels in each strain. The K4me3 levels in the WT strain correlated well with the previously published data (Kirmizis et al., 2007; Pokholok et al., 2005) (Figure S3A). All nondubious genes (n = 5,771) were ranked according to the ratio (from largest to smallest) of the average K4me3 over the transcription unit in the substitution strains relative to the WT strain (Table S1). Genome-wide, we observed a continuum from least to most affected genes (Figure S3B). There was a positive correlation between the ratio of K4me3 (K14A/WT and P16V/WT) and nascent sense transcription (r_S = 0.326 and 0.425, respectively), but no strong correlation with gene length (r_S = −0.063 and −0.003).

The 200 genes that maintained the highest K4me3 in the K14A or P16V stains relative to WT were classed as largely K14/P16-independent whereas the 200 genes that lost the most K4me3 in the H3 substitution strains were classed as most K14/P16-dependent (Table S1; Figures 5A and S3B). These classes were used in gene ontology (GO) (Huang et al., 2009a, 2009b) and overlap analyses, which revealed that the K14/P16-independent and K14/P16-dependent genes function in very different biological processes and show different transcriptional dependencies on SAGA (Table S2; Figures 5B and S3C). Genes with the highest ratios of K4me3, in both the K14A and P16V strains relative to WT, are predominantly involved in translation, particularly ribosomal protein genes (RPGs), and are enriched in genes expressed during the oxidative phase of the YMC and repressed during the ESR. In contrast, K14/P16-dependent genes, with K4me3 most reduced by the K14A or P16V substitutions, are predominantly regulated by SAGA, are involved in the response to environmental stresses and metabolic processes, and tend to

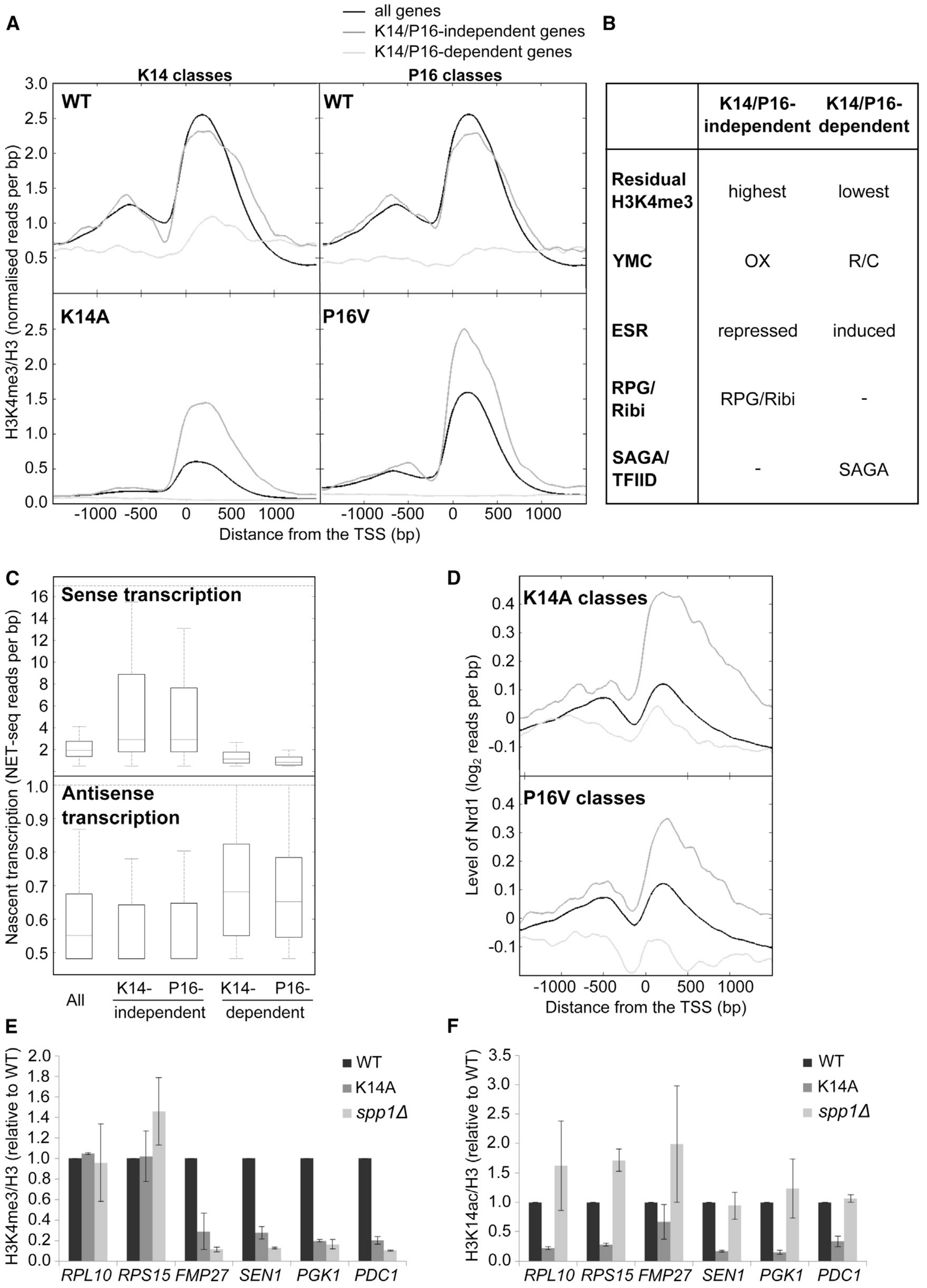

Figure 5. Genome-wide Analysis of K4me3 in the K14A and P16V Strains

(A) ChIP-sequencing experiments showing the median distribution of H3-normalized K4me3 around the transcription start site (TSS, 0) in the WT, K14A and P16V strains for all genes (black line) and the 200 most K14/P16-independent (dark gray) and K14/P16-dependent (light gray) genes after ranking according to the ratio of the average level of H3-normalized K4me3 across the transcription unit in the K14A or P16V substitutions relative to the WT strain.

(legend continued on next page)

be induced during the ESR and reductive phase of the YMC. Interestingly, in the P16V substitution strain, the genes with K4me3 most reduced encode proteins involved in sporulation and meiosis, in addition to some of the stress response pathways common to the K14A-affected genes (Table S2). Despite these differences, the Spearman correlation between the ranked genes from the K14A and P16V strains is 0.772. This suggests that although the general trends are similar, K14 and P16 may contribute to different extents to K4me3 levels at individual genes, consistent with both overlapping and distinct functions.

Further analysis of the 200 K14/P16-independent and K14/P16-dependent genes revealed distinct differences (Figures 5C and 5D). In the WT strain, the K14/P16-independent genes are particularly enriched in nascent sense transcription (Churchman and Weissman, 2011) and Nrd1 (Mayer et al., 2012), one of the few genome-wide transcription termination factors shown to be dependent on K4me3 (Schulz et al., 2013; Terzi et al., 2011), in a pattern distinct from the majority of genes. By contrast, the K14/P16-dependent genes are depleted for Nrd1 and nascent sense transcription and enriched for antisense transcription (as expected due to low Nrd1 levels), suggesting distinct regulation of these genes.

Spp1 and K14 Are Not Essential for K4me3 at Ribosomal Protein Genes

We used ChIP at six genes to validate our genome-wide data and confirm that at two RPGs (*RPS15* and *RPL10*) a strain with a K14A substitution retains high levels of K4me3 (Figure 5E; see also Figure 3C). At four other genes, occupying various positions in the ranking, levels of K4me3 are reduced compared to WT. Given the link between K14 and Spp1, we asked how loss of Spp1 affects K4me3, using ChIP-qPCR at the same six representative genes (Figure 5E). The profile of K4me3 in the *spp1Δ* strain is very similar to the K14A strain. Spp1 is not required for K4me3 at RPGs but is required at the remaining four genes. We observed no significant change in K14ac at these genes in a *spp1Δ* strain that might explain changes in K4me3 (Figure 5F). This suggests that Spp1, like K14, is not essential for K4me3 and further links Spp1 function to the integrity of K14 and P16. We conclude (1) that the integrity of K14 and P16 play a major role in determining K4me3 levels at Spp1-dependent genes, and (2) there may be Spp1-dependent and Spp1-independent forms of Set1C in vivo. Given the similar effects of K14 and P16 substitution on K4me3 on different genes and the results from the K14 P16 double substitution experiments, we wanted to learn more about the relationship between these two residues, particularly how the K14R substitution might differ from other K14 substitutions.

The Integrity of P16 Affects the Binding of Proteins to Adjacent Sites on the Histone H3 Tail

We have already demonstrated that the P16V substitution prevents antibodies raised against K14ac or K18ac from recognizing their epitopes on peptides (see Figure S1C). We developed a second assay that involves monitoring the effect of the P16V substitution on the binding of the Spt7 bromodomain to an H3 peptide modified by acetylation at K14 and/or K18 using surface plasmon resonance (SPR) (Figures 6A, S4A, and S4B) or by pull downs (Figures 6B and S4C). Bromodomains are acetyl-lysine binding domains, found in a number of chromatin-associated proteins (Taverna et al., 2007) including Spt7. The function and binding specificity of the Spt7 bromodomain are currently unknown, but it preferentially interacts with hyperacetylated histones in vitro (Hassan et al., 2007). The Spt7 bromodomain (residues 363–619) specifically recognized acetylated K18, as evidenced by the lack of binding to the unmodified H3 peptide, or a peptide acetylated at K14 (Figures 6A and 6B), or of the bromodomain carrying alanine substitutions in residues known to influence acetyl-lysine binding (Y500, Y520, and N521) (Figure S4C). The binding of the Spt7 bromodomain to the P16V-substituted K18ac peptide improved binding by ~2-fold. This suggests that binding is influenced by P16 and improved with a *trans* conformation. However, no binding was observed to the K14R-K18ac peptide. We reasoned that this could reflect an effect of the side chain on binding or an influence on the conformation of the A15-P16 bond. To test this, we introduced a valine substitution at P16 in the K14R-K18ac peptide, thereby fixing the A15-V16 peptide bond in the *trans* configuration and restored binding at levels between the P16V-K18ac and K18ac peptides. This suggests that in the K14R-K18ac peptide, the A15-P16 peptidyl-prolyl bond adopts the *cis* configuration, reducing binding of the bromodomain to K18ac, which can be restored by fixing the bond in *trans*. The corollary of this is that acetylation of K14 would correlate with the A15-P16 bond in the *trans* configuration, supporting ideas that the peptidyl-prolyl bond conformation is mainly determined by local effects in proteins (Reimer et al., 1998). We tested this using a peptide acetylated at both K14 and K18 and show binding to the Spt7 bromodomain similar to the P16V-K18ac peptide, consistent with our hypothesis that acetylation of K14 influences the conformation of the A15-P16 peptide bond to favor the *trans* configuration.

K14ac Influences the Proportion of A15-P16*trans*

To test more directly the influence of substitutions or modifications at K14 on *cis* versus *trans* conformations of the A15-P16 peptide bond, we used an established protease-coupled proline isomerization assay (Fischer et al., 1984) (Figure 6C). We designed four peptides, in which K14 was unmodified or

(B) Features of genes in the K14/P16-independent and K14/P16-dependent classes (YMC, yeast metabolic cycle; OX, oxidative phase; R/C, reductive charging phase; ESR, environmental stress response; RPGs, ribosomal protein genes; Ribis, ribosome biogenesis genes; SAGA/TFIID-regulated).

(C) Boxplots showing the level of WT sense and antisense nascent transcription (Churchman and Weissman, 2011) in the first 300 bp of the transcription unit for the same gene classes as in (A) (outliers excluded).

(D) Median WT log2 levels of Nrd1 (Mayer et al., 2012) around the TSS for the same gene classes as in (A).

(E and F) ChIP-qPCR experiment showing average levels ± SEM of H3-normalized (E) K4me3 and (F) K14ac at the 5′ ends of the indicated genes in rank order (n = 2).

See also Tables S1 and S2 and Figure S3.

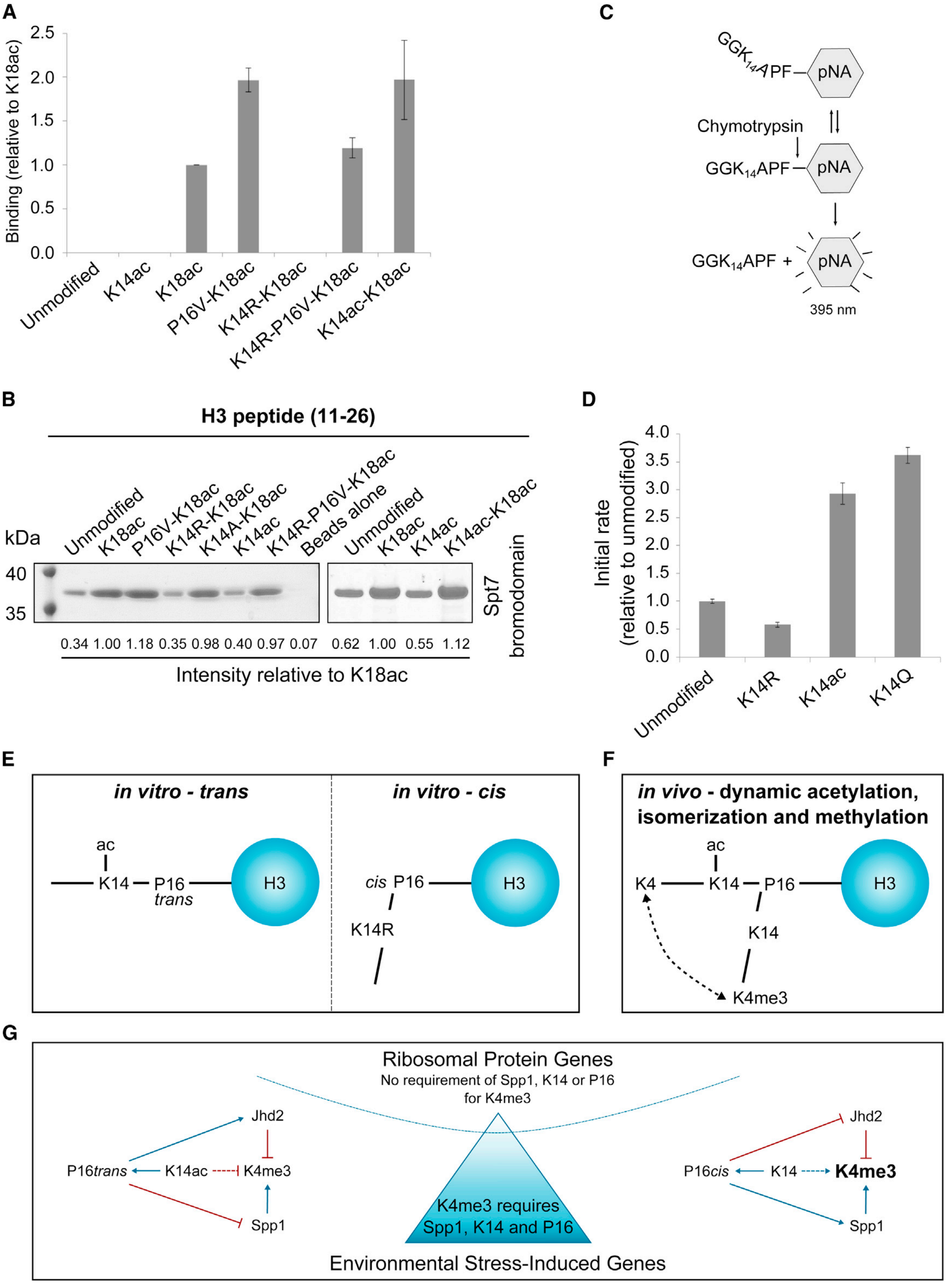

Figure 6. K14ac Promotes P16*trans*

(A) Relative binding ± SEM (n = 2–11) assessed by surface plasmon resonance of purified recombinant Spt7 bromodomain (residues 363–619) to the indicated H3 peptides (11–26).

(B) Coomassie-stained gel showing a pull-down experiment, quantified relative to the K18ac peptide, with the indicated H3 peptides and the purified recombinant Spt7 bromodomain (n = 3).

(legend continued on next page)

acetylated, or substituted with R or Q and where P16 is followed by a phenylalanine and paranitroaniline (F-pNA) group. Chymotrypsin cleaves these peptides to release pNA only if the A15-P16 peptide bond is in the *trans* conformation and with kinetics that are much faster than the spontaneous rate of *cis-trans* isomerization of the proline. Thus, the initial rate of the chymotrypsin-dependent release of pNA, measured spectrophotometrically at 395 nm, is a function of the amount of peptide with a *trans* proline conformation and for the unmodified peptide is comparable to that reported previously in a similar assay (Nelson et al., 2006). Acetylation of K14 increases the initial rate of cleavage of the peptide 3-fold, indicating that a higher proportion of the A15-P16 peptide bond is in the *trans* conformation (Figure 6D). Our data predict that substitutions at K14 will alter the A15-P16 conformation. The K14Q substitution resulted in an increase in the initial rate of cleavage of the peptide, consistent with an increase in the amount of peptide in the *trans* conformation. Crucially, a K14R substitution reduced the initial rate of cleavage of the peptide below the unmodified peptide consistent with an increase in the *cis* conformation. Taken together these data suggest that (1) the conformation of the A15-P16 peptidyl-prolyl bond influences the binding of protein effectors to histone H3, (2) the conformation of the A15-P16 peptidyl-prolyl bond is likely to be influenced by the nature of the residue at n-2 and that acetylation of K14 promotes the *trans* conformation (Figure 6E), and (3) K4me3 is promoted by unmodified K14 increasing A15-P16*cis* (Figure 6F).

DISCUSSION

We have shown that acetylation at K14 influences the local conformation of histone H3 at the A15-P16 peptide bond in vitro, promoting a *trans* conformation, and we describe how these changes reduce levels of K4me3 in vivo. We propose that the H3 tail alternates between two dynamic forms in vivo: one with K14ac and P16*trans* and the other with unmodified K14 and P16*cis* (Figure 6F). Moreover, substitutions at K14 and P16 affect the balance of methylation by Set1C/Spp1 and demethylation by Jhd2 to modulate K4me3 levels. We find that the amount of K4me3 lost on genes in strains lacking Spp1 or with substitutions at K14 or P16 is not uniform. This is particularly evident on genes that are differentially regulated during the yeast metabolic cycle and environmental stress response (Figure 6G). We infer at least two distinct mechanisms for deposition of K4me3, one of which is sensitive to loss of Spp1 from Set1C and the conformation of the N-terminal region of histone H3.

Spp1 has been proposed to be critical for stimulating the trimethylase activity of Set1 in Set1C (Takahashi et al., 2009). A remarkable finding from this study is that K4 trimethylation (K4me3) at the RPGs is largely independent of Spp1 and mostly insensitive to the acetylation status of K14 or *cis-trans* isomerization of P16, which may regulate the association of Spp1 with chromatin. This suggests an additional mechanism for stimulating K4me3 that predominates at RPGs but also functions at other genes to varying extents. Set1 contains RRM RNA binding motifs and the binding of RNA by Set1 is proposed to stimulate its K4me3 activity (Schlichter and Cairns, 2005; Trésaugues et al., 2006). High levels of sense transcription, intronic RNA (Weiner et al., 2012), antisense transcripts (Margaritis et al., 2012; Murray et al., 2012), and Nrd1-attenuated noncoding transcripts (Schulz et al., 2013) at RPGs may contribute to Spp1-independent alternative anchoring of Set1 to this gene class.

At the majority of yeast genes, including ESR-induced genes, maintenance of K4me3 depends on the integrity of K14, A15-P16*cis* (bent) and Spp1. These genes have lower levels of sense transcription that may not be sufficient for the deposition of K4me3 by a K14/P16-independent mechanism. Although often thought of as unstructured, ~50% of the H3 N-terminal tail adopts an α-helical conformation in the nucleosome (Banères et al., 1997). Proline is known to disrupt α helices and may act as hinge points in H3, dramatically affecting the relative trajectories of the flanking helices (Lu et al., 2007) and thus the ability of modifying enzymes to find their substrates. Rather than direct cause and effect, our data support a highly dynamic scenario in which levels of K4me3 are influenced by the action of the Set1C methyltransferase with Spp1 and the Jhd2 demethylase, which in turn are influenced by the modification status of K14 and the conformation of the A15-P16 bond (Figure 6G).

More generally, acetylation in proximity to proline residues may act as a metabolically controlled switch within intrinsically disordered regions of proteins to facilitate the alternative conformations required for interactions with a range of different proteins. Although PPIases promote protein folding by increasing the rate of proline isomerization, in disordered regions the *cis-trans* switch may rely on the natural slower isomerization rate with local PTMs, such as acetylation, influencing one state over the other, perhaps by steric hindrance. In vivo, these states could be captured by binding proteins to induce local folding. In summary, we describe a function for lysine acetylation in determining local protein conformation, in this case histone H3, by directly influencing proline 16 *cis-trans* isomerization. The potential for antibody occlusion in yeast expressing substituted histones or off-target effects resulting from HDAC inhibition preclude further reliable in vivo validation of this relationship, although tools to explore this are being developed.

(C) A chymotrypsin-coupled assay for proline isomers using synthetic hexamers based on GGKAPF-pNA (unmodified). pNA is released by chymotrypsin cleavage only from the *trans*-proline-containing peptide.

(D) K14ac and substitutions at K14 influence isomerization at A15-P16 and pNA release, expressed as mean initial rate ± SEM (n = 4–5) over the first 0.6–10.2 s of the reaction.

(E) Schematic showing how K14ac or the K14R substitution influence A15-P16 conformation.

(F) Schematic showing how dynamic K14ac influences A15-P16 conformation and K4me3 (K4 and K14 denote that these residues are not trimethylated or acetylated respectively).

(G) The balance of positive-acting factors (right panel) and negative-acting factors (left panel) controls levels of K4me3 at individual genes. The middle panel shows the continuum in requirement for Spp1, K14, and P16 for K4me3 from no dependency (RPGs) to most dependent (ESR-induced genes).

See also Figure S4.

K4me3 interacts with a variety of protein effectors in yeast (Vermeulen and Timmers, 2010), such as Spp1, the KAT components Yng1 and Sgf29, the chromatin remodeling ATPase Isw1, and the HDAC complex Rpd3L, potentially leading to acetylation or deacetylation of residues, including K14, in the vicinity. The resulting positive and negative feedback loops, coupled to variable concentrations of intracellular NADPH and acetyl CoA during the metabolic cycle and growth, could facilitate dynamic regulation of K14 acetylation/deacetylation, *cis-trans* isomerization at P16, and K4 demethylation/trimethylation at different gene classes, allowing the rapid switching between transcriptional states and adaptation to changing conditions. Differential control of K4me3 could modulate many other processes, including initiation of DNA replication (Kan et al., 2008), initiation of meiotic recombination (Acquaviva et al., 2013; Borde et al., 2009; Sommermeyer et al., 2013), and epigenetic memory (Muramoto et al., 2010). Given the many different enzymes and proteins capable of methylating, demethylating, and interacting with K4me3 in multicellular organisms and the variety of processes associated with K4me3 (Vermeulen and Timmers, 2010), fine tuning mechanisms such as those described here would extend the versatility of this near universal but still poorly understood histone modification.

EXPERIMENTAL PROCEDURES

Saccharomyces cerevisiae strains used in this study are shown in Supplemental Experimental Procedures. Yeast cells were grown at 30°C, shaking at 200 rpm in YPD, to exponential phase (1.25×10^7 cells/ml) for whole cell extracts, subject to western blots, and visualized using chemiluminescence (Pierce) and exposure to X-ray film. Quantitation was performed using ImageJ software. Peptide synthesis and antibody production to raise polyclonal antibodies against $P16_{OH}$*cis* and $P16_{OH}$*trans* peptides were performed by Pacific Immunology and antibody affinity purified with 90 μg $P16_{OH}$*trans* or $P16_{OH}$*cis* peptides, respectively and 90 μg unmodified H3 peptides dotted onto nitrocellulose for 48 hr at 4°C. The P16 proline isomerase assay was performed as described (Shan et al., 1994) using 10 μl of 7.8 mM pNA peptide (Proteogenix) solution per 1 ml assay reaction and pNA release monitored at 395 nm. ChIP-qPCR was performed as described (Morillon et al., 2005). ChIP-seq was performed with 10 ng of immunoprecipitated DNA. DNA was multiplexed during library preparation and subjected to 50 nt paired-end single lane sequencing. Peptides for bromodomain interactions studies were synthesized at GL Biochem and Proteogenix. The peptides used in these experiments had a minimum purity, determined by MALDI-TOF mass spectrometry and HPLC, of 84%. Spt7 bromodomain (amino acids 363–619) was expressed and purified as described (Boubriak et al., 2009). SulfoLink Coupling Resin (Thermo Scientific, Cat. 20401) was used in all peptide pull-downs experiments. Peptides were coupled to the resin according to the manufacturer's instructions. Resin (200 μl) was incubated with 350 μl of bromodomain (final concentration of 30 μg/ml) for 2 hr at 4°C. After multiple washes with binding buffer, high salt (350 mM NaCl), and TE buffer, bound material was eluted by heating at 80°C with 120 μl LDS (Invitrogen). SPR data for peptides binding to immobilized Spt7 bromodomain was generated on Biacore T100 and T200 instruments at 25°C. Sensorgrams, binding curves, and K_d values were analyzed with BIA T100 and T200 evaluation software (GE Healthcare) using a 1:1 binding model. Detailed protocols, including data analysis, can be found in Supplemental Experimental Procedures.

ACCESSION NUMBERS

ChIP-seq data are available in the ArrayExpress database (http://www.ebi.ac.uk/arrayexpress) under accession number E-MTAB-2717.

SUPPLEMENTAL INFORMATION

Supplemental Information includes Supplemental Experimental Procedures, four figures, and two tables and can be found with this article online at http://dx.doi.org/10.1016/j.molcel.2014.07.004.

ACKNOWLEDGMENTS

The authors would like to thank Ali Shilatifard for helpful discussion, Rob Klose for advice on ChIP-seq experiments, Ben Thomas for mass spectrometry analysis, Sharon Dent and Michael Grunstein for yeast strains, the High-Throughput Genomics Group at the Wellcome Trust Centre for Human Genetics (Wellcome Trust grant 090532/Z/09/Z and Medical Research Council [MRC] Hub grant G0900747 91070), and the following funding agencies: The Engineering and Physical Sciences Research Council (to S.C.M. and R.W.), the Wellcome Trust (to F.S.H. and A.G.), Oxford Biodynamics (ALRNEI1 to J.M.), Epigenesys Network of Excellence (to J.M.), and the Wellcome Trust (WT089156MA to J.M.).

Received: December 23, 2013
Revised: May 15, 2014
Accepted: July 2, 2014
Published: August 7, 2014

REFERENCES

Acquaviva, L., Székvölgyi, L., Dichtl, B., Dichtl, B.S., de La Roche Saint André, C., Nicolas, A., and Géli, V. (2013). The COMPASS subunit Spp1 links histone methylation to initiation of meiotic recombination. Science *339*, 215–218.

Baker, S.P., and Grant, P.A. (2007). The SAGA continues: expanding the cellular role of a transcriptional co-activator complex. Oncogene *26*, 5329–5340.

Banères, J.L., Martin, A., and Parello, J. (1997). The N tails of histones H3 and H4 adopt a highly structured conformation in the nucleosome. J. Mol. Biol. *273*, 503–508.

Borde, V., Robine, N., Lin, W., Bonfils, S., Géli, V., and Nicolas, A. (2009). Histone H3 lysine 4 trimethylation marks meiotic recombination initiation sites. EMBO J. *28*, 99–111.

Boubriak, I., Mason, P.A., Clancy, D.J., Dockray, J., Saunders, R.D., and Cox, L.S. (2009). DmWRNexo is a 3′-5′ exonuclease: phenotypic and biochemical characterization of mutants of the Drosophila orthologue of human WRN exonuclease. Biogerontology *10*, 267–277.

Brauer, M.J., Huttenhower, C., Airoldi, E.M., Rosenstein, R., Matese, J.C., Gresham, D., Boer, V.M., Troyanskaya, O.G., and Botstein, D. (2008). Coordination of growth rate, cell cycle, stress response, and metabolic activity in yeast. Mol. Biol. Cell *19*, 352–367.

Briggs, S.D., Bryk, M., Strahl, B.D., Cheung, W.L., Davie, J.K., Dent, S.Y.R., Winston, F., and Allis, C.D. (2001). Histone H3 lysine 4 methylation is mediated by Set1 and required for cell growth and rDNA silencing in Saccharomyces cerevisiae. Genes Dev. *15*, 3286–3295.

Briggs, S.D., Xiao, T., Sun, Z.W., Caldwell, J.A., Shabanowitz, J., Hunt, D.F., Allis, C.D., and Strahl, B.D. (2002). Gene silencing: trans-histone regulatory pathway in chromatin. Nature *418*, 498.

Bryk, M., Briggs, S.D., Strahl, B.D., Curcio, M.J., Allis, C.D., and Winston, F. (2002). Evidence that Set1, a factor required for methylation of histone H3, regulates rDNA silencing in S. cerevisiae by a Sir2-independent mechanism. Curr. Biol. *12*, 165–170.

Cai, L., Sutter, B.M., Li, B., and Tu, B.P. (2011). Acetyl-CoA induces cell growth and proliferation by promoting the acetylation of histones at growth genes. Mol. Cell *42*, 426–437.

Carvin, C.D., and Kladde, M.P. (2004). Effectors of lysine 4 methylation of histone H3 in Saccharomyces cerevisiae are negative regulators of PHO5 and GAL1-10. J. Biol. Chem. *279*, 33057–33062.

Churchman, L.S., and Weissman, J.S. (2011). Nascent transcript sequencing visualizes transcription at nucleotide resolution. Nature *469*, 368–373.

CellPress

Fingerman, I.M., Wu, C.L., Wilson, B.D., and Briggs, S.D. (2005). Global loss of Set1-mediated H3 Lys4 trimethylation is associated with silencing defects in Saccharomyces cerevisiae. J. Biol. Chem. *280*, 28761–28765.

Fischer, G., Bang, H., Berger, E., and Schellenberger, A. (1984). Conformational specificity of chymotrypsin toward proline-containing substrates. Biochim. Biophys. Acta *791*, 87–97.

Gasch, A.P., Spellman, P.T., Kao, C.M., Carmel-Harel, O., Eisen, M.B., Storz, G., Botstein, D., and Brown, P.O. (2000). Genomic expression programs in the response of yeast cells to environmental changes. Mol. Biol. Cell *11*, 4241–4257.

Govind, C.K., Zhang, F., Qiu, H., Hofmeyer, K., and Hinnebusch, A.G. (2007). Gcn5 promotes acetylation, eviction, and methylation of nucleosomes in transcribed coding regions. Mol. Cell *25*, 31–42.

Guillemette, B., Drogaris, P., Lin, H.H., Armstrong, H., Hiragami-Hamada, K., Imhof, A., Bonneil, E., Thibault, P., Verreault, A., and Festenstein, R.J. (2011). H3 lysine 4 is acetylated at active gene promoters and is regulated by H3 lysine 4 methylation. PLoS Genet. *7*, e1001354.

Hassan, A.H., Awad, S., Al-Natour, Z., Othman, S., Mustafa, F., and Rizvi, T.A. (2007). Selective recognition of acetylated histones by bromodomains in transcriptional co-activators. Biochem. J. *402*, 125–133.

Huang, W., Sherman, B.T., and Lempicki, R.A. (2009a). Bioinformatics enrichment tools: paths toward the comprehensive functional analysis of large gene lists. Nucleic Acids Res. *37*, 1–13.

Huang, W., Sherman, B.T., and Lempicki, R.A. (2009b). Systematic and integrative analysis of large gene lists using DAVID bioinformatics resources. Nat. Protoc. *4*, 44–57.

Ingvarsdottir, K., Edwards, C., Lee, M.G., Lee, J.S., Schultz, D.C., Shilatifard, A., Shiekhattar, R., and Berger, S.L. (2007). Histone H3 K4 demethylation during activation and attenuation of GAL1 transcription in Saccharomyces cerevisiae. Mol. Cell. Biol. *27*, 7856–7864.

Jiang, L., Smith, J.N., Anderson, S.L., Ma, P., Mizzen, C.A., and Kelleher, N.L. (2007). Global assessment of combinatorial post-translational modification of core histones in yeast using contemporary mass spectrometry. LYS4 trimethylation correlates with degree of acetylation on the same H3 tail. J. Biol. Chem. *282*, 27923–27934.

Kan, J., Zou, L., Zhang, J., Wu, R., Wang, Z., and Liang, C. (2008). Origin recognition complex (ORC) mediates histone 3 lysine 4 methylation through cooperation with Spp1 in Saccharomyces cerevisiae. J. Biol. Chem. *283*, 33803–33807.

Kirmizis, A., Santos-Rosa, H., Penkett, C.J., Singer, M.A., Vermeulen, M., Mann, M., Bähler, J., Green, R.D., and Kouzarides, T. (2007). Arginine methylation at histone H3R2 controls deposition of H3K4 trimethylation. Nature *449*, 928–932.

Lenstra, T.L., Benschop, J.J., Kim, T., Schulze, J.M., Brabers, N.A., Margaritis, T., van de Pasch, L.A., van Heesch, S.A., Brok, M.O., Groot Koerkamp, M.J., et al. (2011). The specificity and topology of chromatin interaction pathways in yeast. Mol. Cell *42*, 536–549.

Liang, G., Klose, R.J., Gardner, K.E., and Zhang, Y. (2007). Yeast Jhd2p is a histone H3 Lys4 trimethyl demethylase. Nat. Struct. Mol. Biol. *14*, 243–245.

Liu, C.L., Kaplan, T., Kim, M., Buratowski, S., Schreiber, S.L., Friedman, N., and Rando, O.J. (2005). Single-nucleosome mapping of histone modifications in S. cerevisiae. PLoS Biol. *3*, e328.

Lu, K.P., Finn, G., Lee, T.H., and Nicholson, L.K. (2007). Prolylcis-trans isomerization as a molecular timer. Nat. Chem. Biol. *3*, 619–629.

Lu, C., Brauer, M.J., and Botstein, D. (2009). Slow growth induces heat-shock resistance in normal and respiratory-deficient yeast. Mol. Biol. Cell *20*, 891–903.

Maltby, V.E., Martin, B.J., Brind'Amour, J., Chruscicki, A.T., McBurney, K.L., Schulze, J.M., Johnson, I.J., Hills, M., Hentrich, T., Kobor, M.S., et al. (2012). Histone H3K4 demethylation is negatively regulated by histone H3 acetylation in Saccharomyces cerevisiae. Proc. Natl. Acad. Sci. USA *109*, 18505–18510.

Margaritis, T., Oreal, V., Brabers, N., Maestroni, L., Vitaliano-Prunier, A., Benschop, J.J., van Hooff, S., van Leenen, D., Dargemont, C., Géli, V., and Holstege, F.C. (2012). Two distinct repressive mechanisms for histone 3 lysine 4 methylation through promoting 3′-end antisense transcription. PLoS Genet. *8*, e1002952.

Mayer, A., Heidemann, M., Lidschreiber, M., Schreieck, A., Sun, M., Hintermair, C., Kremmer, E., Eick, D., and Cramer, P. (2012). CTD tyrosine phosphorylation impairs termination factor recruitment to RNA polymerase II. Science *336*, 1723–1725.

Monneau, Y.R., Soufari, H., Nelson, C.J., and Mackereth, C.D. (2013). Structure and activity of the peptidyl-prolyl isomerase domain from the histone chaperone Fpr4 toward histone H3 proline isomerization. J. Biol. Chem. *288*, 25826–25837.

Morillon, A., Karabetsou, N., Nair, A., and Mellor, J. (2005). Dynamic lysine methylation on histone H3 defines the regulatory phase of gene transcription. Mol. Cell *18*, 723–734.

Muramoto, T., Müller, I., Thomas, G., Melvin, A., and Chubb, J.R. (2010). Methylation of H3K4 Is required for inheritance of active transcriptional states. Curr. Biol. *20*, 397–406.

Murray, S.C., Serra Barros, A., Brown, D.A., Dudek, P., Ayling, J., and Mellor, J. (2012). A pre-initiation complex at the 3′-end of genes drives antisense transcription independent of divergent sense transcription. Nucleic Acids Res. *40*, 2432–2444.

Murton, B.L., Chin, W.L., Ponting, C.P., and Itzhaki, L.S. (2010). Characterising the binding specificities of the subunits associated with the KMT2/Set1 histone lysine methyltransferase. J. Mol. Biol. *398*, 481–488.

Nagy, P.L., Griesenbeck, J., Kornberg, R.D., and Cleary, M.L. (2002). A trithorax-group complex purified from Saccharomyces cerevisiae is required for methylation of histone H3. Proc. Natl. Acad. Sci. USA *99*, 90–94.

Nakanishi, S., Sanderson, B.W., Delventhal, K.M., Bradford, W.D., Staehling-Hampton, K., and Shilatifard, A. (2008). A comprehensive library of histone mutants identifies nucleosomal residues required for H3K4 methylation. Nat. Struct. Mol. Biol. *15*, 881–888.

Nelson, C.J., Santos-Rosa, H., and Kouzarides, T. (2006). Proline isomerization of histone H3 regulates lysine methylation and gene expression. Cell *126*, 905–916.

O'Duibhir, E., Lijnzaad, P., Benschop, J.J., Lenstra, T.L., van Leenen, D., Groot Koerkamp, M.J., Margaritis, T., Brok, M.O., Kemmeren, P., and Holstege, F.C. (2014). Cell cycle population effects in perturbation studies. Mol. Syst. Biol. *10*, 732.

Pokholok, D.K., Harbison, C.T., Levine, S., Cole, M., Hannett, N.M., Lee, T.I., Bell, G.W., Walker, K., Rolfe, P.A., Herbolsheimer, E., et al. (2005). Genome-wide map of nucleosome acetylation and methylation in yeast. Cell *122*, 517–527.

Radman-Livaja, M., Liu, C.L., Friedman, N., Schreiber, S.L., and Rando, O.J. (2010). Replication and active demethylation represent partially overlapping mechanisms for erasure of H3K4me3 in budding yeast. PLoS Genet. *6*, e1000837.

Rando, O.J., and Winston, F. (2012). Chromatin and transcription in yeast. Genetics *190*, 351–387.

Reimer, U., Scherer, G., Drewello, M., Kruber, S., Schutkowski, M., and Fischer, G. (1998). Side-chain effects on peptidyl-prolylcis/trans isomerisation. J. Mol. Biol. *279*, 449–460.

Roguev, A., Schaft, D., Shevchenko, A., Aasland, R., Shevchenko, A., and Stewart, A.F. (2003). High conservation of the Set1/Rad6 axis of histone 3 lysine 4 methylation in budding and fission yeasts. J. Biol. Chem. *278*, 8487–8493.

Santos-Rosa, H., Schneider, R., Bannister, A.J., Sherriff, J., Bernstein, B.E., Emre, N.C., Schreiber, S.L., Mellor, J., and Kouzarides, T. (2002). Active genes are tri-methylated at K4 of histone H3. Nature *419*, 407–411.

Schlichter, A., and Cairns, B.R. (2005). Histone trimethylation by Set1 is coordinated by the RRM, autoinhibitory, and catalytic domains. EMBO J. *24*, 1222–1231.

Schulz, D., Schwalb, B., Kiesel, A., Baejen, C., Torkler, P., Gagneur, J., Soeding, J., and Cramer, P. (2013). Transcriptome surveillance by selective termination of noncoding RNA synthesis. Cell *155*, 1075–1087.

Seward, D.J., Cubberley, G., Kim, S., Schonewald, M., Zhang, L., Tripet, B., and Bentley, D.L. (2007). Demethylation of trimethylated histone H3 Lys4 in vivo by JARID1 JmjC proteins. Nat. Struct. Mol. Biol. *14*, 240–242.

Shan, X., Xue, Z., and Mélèse, T. (1994). Yeast NPI46 encodes a novel prolylcis-trans isomerase that is located in the nucleolus. J. Cell Biol. *126*, 853–862.

Shi, L., and Tu, B.P. (2013). Acetyl-CoA induces transcription of the key G1 cyclin CLN3 to promote entry into the cell division cycle in Saccharomyces cerevisiae. Proc. Natl. Acad. Sci. USA *110*, 7318–7323.

Shi, X., Kachirskaia, I., Walter, K.L., Kuo, J.-H.A., Lake, A., Davrazou, F., Chan, S.M., Martin, D.G.E., Fingerman, I.M., Briggs, S.D., et al. (2007). Proteome-wide analysis in Saccharomyces cerevisiae identifies several PHD fingers as novel direct and selective binding modules of histone H3 methylated at either lysine 4 or lysine 36. J. Biol. Chem. *282*, 2450–2455.

Slavov, N., Airoldi, E.M., van Oudenaarden, A., and Botstein, D. (2012). A conserved cell growth cycle can account for the environmental stress responses of divergent eukaryotes. Mol. Biol. Cell *23*, 1986–1997.

Sommermeyer, V., Béneut, C., Chaplais, E., Serrentino, M.E., and Borde, V. (2013). Spp1, a member of the Set1 Complex, promotes meiotic DSB formation in promoters by tethering histone H3K4 methylation sites to chromosome axes. Mol. Cell *49*, 43–54.

Takahashi, Y.H., Lee, J.S., Swanson, S.K., Saraf, A., Florens, L., Washburn, M.P., Trievel, R.C., and Shilatifard, A. (2009). Regulation of H3K4 trimethylation via Cps40 (Spp1) of COMPASS is monoubiquitination independent: implication for a Phe/Tyr switch by the catalytic domain of Set1. Mol. Cell. Biol. *29*, 3478–3486.

Taverna, S.D., Li, H., Ruthenburg, A.J., Allis, C.D., and Patel, D.J. (2007). How chromatin-binding modules interpret histone modifications: lessons from professional pocket pickers. Nat. Struct. Mol. Biol. *14*, 1025–1040.

Taylor, C.M., Hardré, R., and Edwards, P.J. (2005). The impact of pyrrolidine hydroxylation on the conformation of proline-containing peptides. J. Org. Chem. *70*, 1306–1315.

Terzi, N., Churchman, L.S., Vasiljeva, L., Weissman, J., and Buratowski, S. (2011). H3K4 trimethylation by Set1 promotes efficient termination by the Nrd1-Nab3-Sen1 pathway. Mol. Cell. Biol. *31*, 3569–3583.

Trésaugues, L., Dehé, P.M., Guérois, R., Rodriguez-Gil, A., Varlet, I., Salah, P., Pamblanco, M., Luciano, P., Quevillon-Cheruel, S., Sollier, J., et al. (2006). Structural characterization of Set1 RNA recognition motifs and their role in histone H3 lysine 4 methylation. J. Mol. Biol. *359*, 1170–1181.

Tu, B.P., Kudlicki, A., Rowicka, M., and McKnight, S.L. (2005). Logic of the yeast metabolic cycle: temporal compartmentalization of cellular processes. Science *310*, 1152–1158.

Tu, S., Bulloch, E.M., Yang, L., Ren, C., Huang, W.C., Hsu, P.H., Chen, C.H., Liao, C.L., Yu, H.M., Lo, W.S., et al. (2007). Identification of histone demethylases in Saccharomyces cerevisiae. J. Biol. Chem. *282*, 14262–14271.

Vermeulen, M., and Timmers, H.T. (2010). Grasping trimethylation of histone H3 at lysine 4. Epigenomics *2*, 395–406.

Wang, X., and Hayes, J.J. (2008). Acetylation mimics within individual core histone tail domains indicate distinct roles in regulating the stability of higher-order chromatin structure. Mol. Cell. Biol. *28*, 227–236.

Wang, S.S., Zhou, B.O., and Zhou, J.Q. (2011). Histone H3 lysine 4 hypermethylation prevents aberrant nucleosome remodeling at the PHO5 promoter. Mol. Cell. Biol. *31*, 3171–3181.

Weiner, A., Chen, H.V., Liu, C.L., Rahat, A., Klien, A., Soares, L., Gudipati, M., Pfeffner, J., Regev, A., Buratowski, S., et al. (2012). Systematic dissection of roles for chromatin regulators in a yeast stress response. PLoS Biol. *10*, e1001369.

Xu, M., Soloveychik, M., Ranger, M., Schertzberg, M., Shah, Z., Raisner, R., Venkatasubrahmanyan, S., Tsui, K., Gebbia, M., Hughes, T., et al. (2012). Timing of transcriptional quiescence during gametogenesis is controlled by global histone H3K4 demethylation. Dev. Cell *23*, 1059–1071.

Youdell, M.L., Kizer, K.O., Kisseleva-Romanova, E., Fuchs, S.M., Duro, E., Strahl, B.D., and Mellor, J. (2008). Roles for Ctk1 and Spt6 in regulating the different methylation states of histone H3 lysine 36. Mol. Cell. Biol. *28*, 4915–4926.

Zhang, W., Bone, J.R., Edmondson, D.G., Turner, B.M., and Roth, S.Y. (1998). Essential and redundant functions of histone acetylation revealed by mutation of target lysines and loss of the Gcn5p acetyltransferase. EMBO J. *17*, 3155–3167.

Molecular Cell, Volume *55*

Supplemental Information

Lysine Acetylation Controls Local Protein Conformation by Influencing Proline Isomerization

Françoise S. Howe, Ivan Boubriak, Matthew J. Sale, Anitha Nair, David Clynes, Anne Grijzenhout, Struan C. Murray, Ronja Woloszczuk, and Jane Mellor

Supplemental Figures

Figure S1

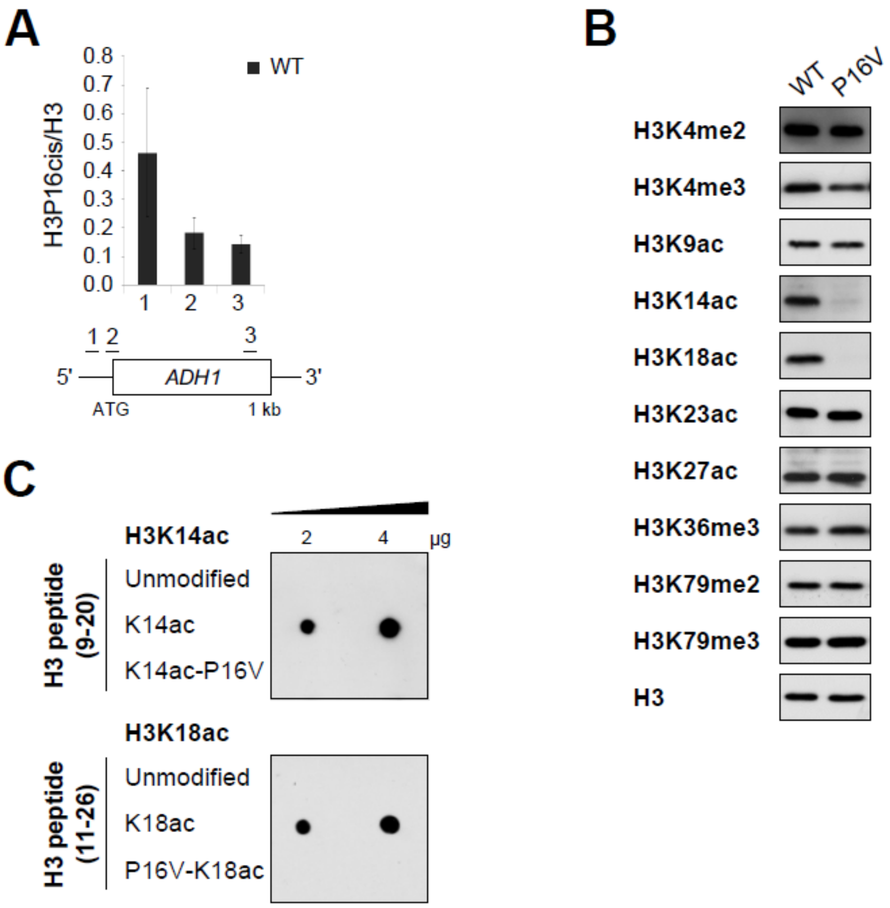

D

	H3 residue number	Observed mass	Mr (experimental)	Mr (calculated)	ppm	Sequence	Modification	Ion score	Peptide intensity
WT	9-17	315.1813	942.5222	942.5247	-2.5	R.KSTGGKAPR.K	ac (K14)	33	7774515
WT	9-17	472.2693	942.5241	942.5247	-0.6	R.KSTGGKAPR.K	ac (K14)	32	31570026
WT	9-17	493.2741	984.5337	984.5352	-1.5	K.KSTGGKAPR.K	2 ac (K9, K14)	51	1040235739
WT	10-17	408.2211	814.4277	814.4297	-2.0	K.STGGKAPR.K	ac (K14)	27	28907756
WT	18-23	358.7187	715.4228	715.4228	0.0	R.KQLASK.A	ac (K18)	37	15473324
WT	18-26	507.8058	1013.597	1013.5981	-1.0	R.KQLASKAAR.K	ac (K23)	52	1045888087
WT	18-26	338.8733	1013.598	1013.5981	-0.1	R.KQLASKAAR.K	ac (K23)	29	918621887
WT	18-26	528.8113	1055.608	1055.6087	-0.7	R.KQLASKAAR.K	2 ac (K18, K23)	56	1268670862
P16V	9-17	473.2771	944.5396	944.5403	-0.7	R.KSTGGKAVR.K	ac (K14)	14	3913804
P16V	9-17	494.2820	986.5494	986.5509	-1.5	R.KSTGGKAVR.K	2 ac (K9, K14)	73	323924046
P16V	10-17	409.2296	816.4446	816.4454	-0.8	K.STGGKAVR.K	ac (K14)	47	1333637
P16V	18-26	507.8060	1013.5974	1013.5981	-0.7	R.KOLASKAAR.K	ac (K23)	9	547378575
P16V	18-26	338.8736	1013.5991	1013.5981	0.9	R.KQLASKAAR.K	ac (K23)	30	471372872
P16V	18-26	528.8119	1055.6093	1055.6087	0.6	R.KQLASKAAR.K	2 ac (K18, K23)	69	24225076

Figure S2

A

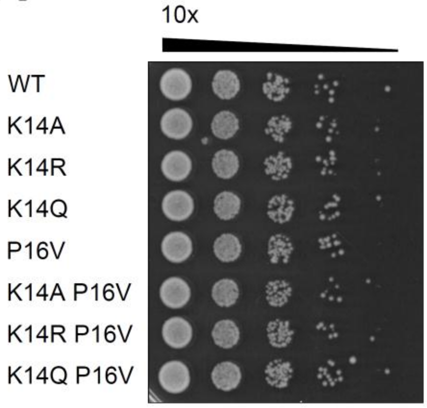

B

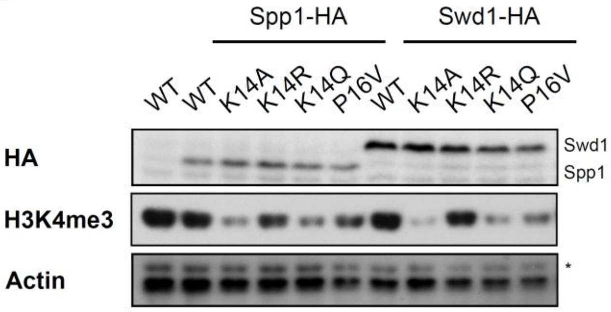

C

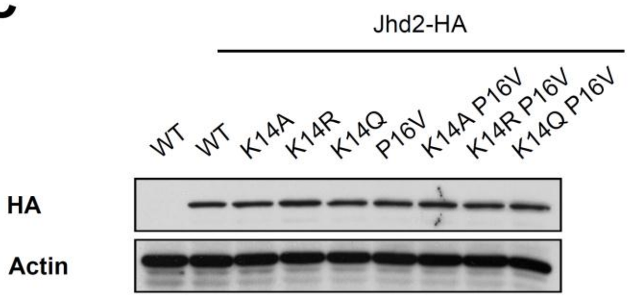

Figure S3

A

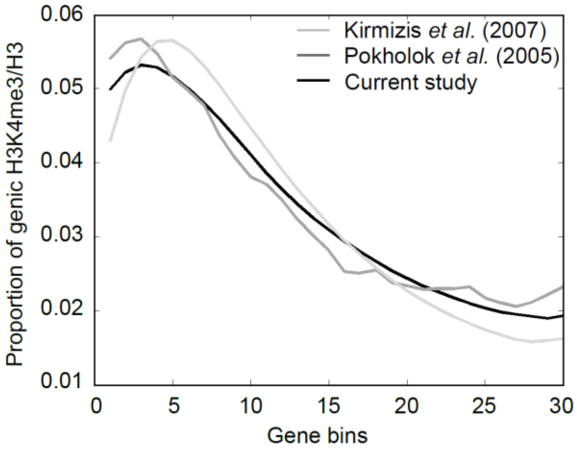

B

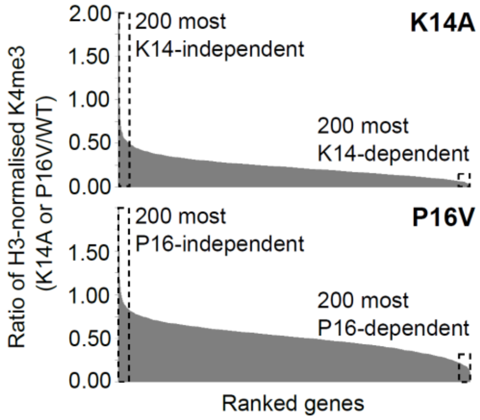

C

% overlap	All genes in study	K14-independent	P16-independent	K14-dependent	P16-dependent
Residual K4me3/H3	-	highest	highest	lowest	lowest
YMC: OX	15.5	**28.5** $p = 1 \times 10^{-5}$	**24.0** $p = 3.7 \times 10^{-4}$	13.5 $p = 0.192$	8.0 $p = 3.5 \times 10^{-4}$
YMC: R/B	14.8	8.0 $p = 1.2 \times 10^{-3}$	12.0 $p = 0.103$	12.0 $p = 0.102$	11.0 $p = 4.6 \times 10^{-2}$
YMC: R/C	23.0	10.0 $p < 1.0 \times 10^{-5}$	8.0 $p < 1 \times 10^{-5}$	**29.5** $p = 1.1 \times 10^{-2}$	**30.0** $p = 7.3 \times 10^{-3}$
ESR: induced	4.5	0.5 $p = 1.5 \times 10^{-4}$	0.5 $p = 1.5 \times 10^{-4}$	**11.5** $p < 1.0 \times 10^{-5}$	**10.5** $p = 5.0 \times 10^{-5}$
ESR: repressed	9.8	**41.0** $p < 1.0 \times 10^{-5}$	**32.5** $p < 1.0 \times 10^{-5}$	0.0 $p < 1.0 \times 10^{-5}$	0.0 $p < 1.0 \times 10^{-5}$
RPG	2.1	**27.5** $p < 1.0 \times 10^{-5}$	**22.5** $p < 1.0 \times 10^{-5}$	0.0 $p < 1.0 \times 10^{-5}$	0.0 $p < 1.0 \times 10^{-5}$
Ribi	2.4	**7.5** $p = 3.0 \times 10^{-5}$	**5.5** $p = 3.3 \times 10^{-3}$	0.0 $p < 1.0 \times 10^{-5}$	0.0 $p < 1.0 \times 10^{-5}$
SAGA-dominant	9.3	1.5 $p < 1.0 \times 10^{-5}$	2.5 $p = 5.0 \times 10^{-5}$	**15.0** $p = 9.7 \times 10^{-4}$	**14.5** $p = 2.3 \times 10^{-3}$
TFIID-dominant	82.4	78.0 $p = 0.456$	79.5 $p = 0.275$	53 $p < 1.0 \times 10^{-5}$	48.5 $p < 1.0 \times 10^{-5}$
SAGA/TFIID-dominant	2.7	2.5 $p = 0.434$	2.0 $p = 0.254$	**10.0** $p < 1.0 \times 10^{-5}$	**14.0** $p < 1.0 \times 10^{-5}$

Figure S4

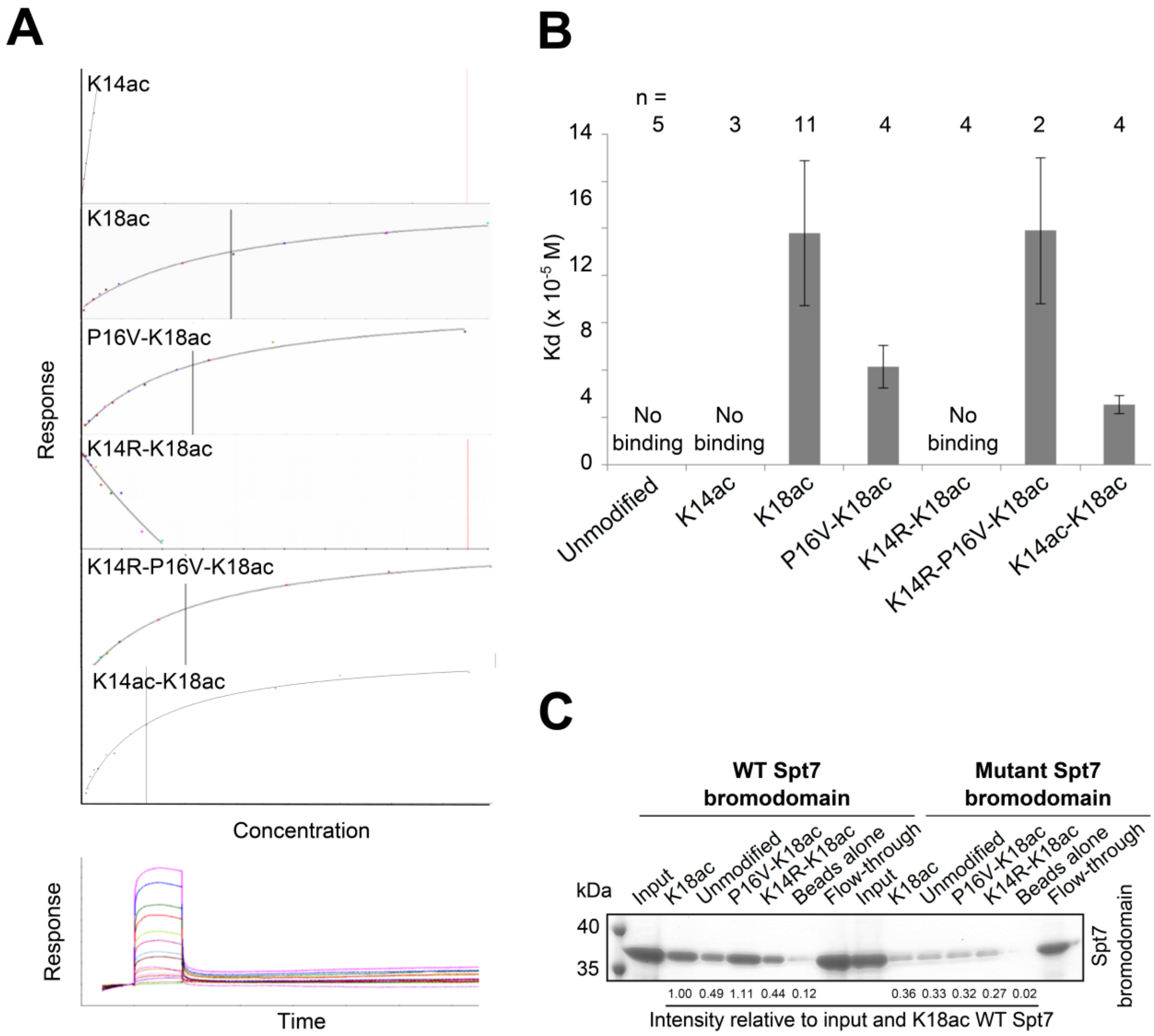

A
K14ac
K18ac
P16V-K18ac
K14R-K18ac
K14R-P16V-K18ac
K14ac-K18ac
Response
Concentration
Response
Time
B
n =
5
3
11
4
4
2
4
Kd (x 10^{-5} M)
14
16
12
8
4
0
No binding
No binding
No binding
Unmodified
K14ac
K18ac
P16V-K18ac
K14R-K18ac
K14R-P16V-K18ac
K14ac-K18ac
C
WT Spt7 bromodomain
Mutant Spt7 bromodomain
kDa
Input
K18ac
Unmodified
P16V-K18ac
K14R-K18ac
Beads alone
Flow-through
Input
K18ac
Unmodified
P16V-K18ac
K14R-K18ac
Beads alone
Flow-through
40
35
Spt7 bromodomain
1.00 0.49 1.11 0.44 0.12
0.36 0.33 0.32 0.27 0.02
Intensity relative to input and K18ac WT Spt7

Supplemental Figure Legends

Figure S1. (Related to Figure 1)

(A) ChIP-qPCR for H3-normalised P16*cis* at *ADH1.* **(B)** Western blots showing the levels of a range of histone H3 modifications in the WT and P16V strains. Histone H3 levels are shown as a loading control. **(C)** Dot blots of 2 and 4 µg of P16-substituted acetylated H3 peptides encompassing residues 9-20 or 11-26 incubated with antibodies raised to K14ac and K18ac. **(D)** Mass spectrometry data showing the presence of K14ac and K18ac in the P16V strain. Each row displays a separate H3 tryptic peptide. The corresponding H3 residue number, observed mass, experimental molecular weight (Mr), calculated Mr, parts per million (ppm, difference between experimental and calculated mass), peptide sequence, modification, ion score and peak intensity are shown for each peptide.

Figure S2. (Related to Figures 3 and 4)

(A) Drop plate growth assay (2 days at 30°C) showing 10-fold serial dilutions on YPD-agar. **(B-C)** Western blots demonstrating the equal global levels of **(B)** Spp1-HA, Swd1-HA and **(C)** Jhd2-HA in the WT and K14/P16 substitution strains. Actin levels are shown as a loading control (* indicates non-specific band). K4me3 levels are included to control for any changes that might be caused by the HA tagging of Spp1.

Figure S3. (Related to Figure 5)

(A) The average WT distribution profile of K4me3 normalised to histone H3 levels across all genes in *S. cerevisiae* from the present (black), (Pokholok et al., 2005) (dark grey) and (Kirmizis et al., 2007) (light grey) datasets. Genes were divided into 30 bins from the transcription start site to the transcription termination site and the level of K4me3 in each

bin expressed as a proportion of the total K4me3 across the gene. **(B)** All non-dubious genes (n=5771) with the ratios of H3-normalised K4me3 in the K14A and P16V strains relative to the WT strain. Genes with ratios > 2 (n = 7 and 4 for K14A/WT and P16V/WT respectively) have been removed for clarity. Highlighted are the genes with the 200 highest and lowest ratios selected for further analysis (K14/P16-independent and K14/P16-dependent gene classes respectively). **(C)** Overlap (%) between the four selected gene classes from Figure S3B and the genes temporally expressed during the yeast metabolic cycle (YMC: OX, oxidative; R/B, reductive building; R/C, reductive charging phase), induced/repressed by the environmental stress response (ESR), classed as ribosomal protein genes (RPGs) or ribosome biogenesis genes (Ribis), or regulated predominantly by SAGA or TFIID. Bold text indicates significant ($p < 0.05$) enrichment of genes in that class for a particular feature compared to the rest of the genome. P values were calculated from a simulated probability distribution curve. YMC and ESR expression data were taken from (Tu et al., 2005) and (Gasch et al., 2000) respectively. SAGA/TFIID-regulated genes were obtained from (Huisinga and Pugh, 2004).

Figure S4. (Related to Figure 6)

(A) Surface plasmon resonance (SPR) affinity fits for the indicated H3 peptides encompassing residues 11-26 binding to the Spt7 bromodomain (residues 363-619). Also shown is a typical sensorgram of H3 peptide binding to the Spt7 bromodomain demonstrating the low background shift and plateauing upon binding. **(B)** Raw average Kd values ± SEM for Spt7 binding to variously-modified H3 peptides. **(C)** A peptide pull-down experiment with a range of H3 peptides and the WT or substituted Spt7 bromodomain

(Y500A/Y520A/N521A) confirming the requirement for an intact bromodomain for enriched binding to the K18ac over the unmodified peptides.

Table S2. Gene ontology analysis of the 200 most K14/P16-independent and K14/P16-dependent genes (related to Figure 4)

**GO terms for the 200 most K14-independent genes**			
**Biological process**	**Count**	**%**	**P value**
translation	66	33.8	9.20E-16
ribosome biogenesis	42	21.5	1.30E-11
ribonucleoprotein complex biogenesis	44	22.6	2.70E-11
regulation of cellular protein metabolic process	24	12.3	8.40E-06
regulation of translation	22	11.3	2.00E-05
rRNA processing	25	12.8	3.40E-05
post-transcriptional regulation of gene expression	22	11.3	4.20E-05
rRNA metabolic process	25	12.8	5.10E-05
maturation of SSU-rRNA from tricistronic rRNA transcript (SSU-rRNA, 5.8S rRNA, LSU-rRNA)	14	7.2	1.10E-04
maturation of SSU-rRNA	14	7.2	1.30E-04
ribosome assembly	12	6.2	5.00E-04
ncRNA processing	27	13.8	7.30E-04
rRNA export from nucleus	9	4.6	2.00E-03
rRNA transport	9	4.6	2.00E-03
ribonucleoprotein complex assembly	14	7.2	3.20E-03
ncRNA metabolic process	28	14.4	3.50E-03
RNA processing	33	16.9	4.60E-03
RNA export from nucleus	11	5.6	2.40E-02
ribosomal small subunit biogenesis	8	4.1	4.50E-02
translational termination	6	3.1	4.60E-02
ribosomal subunit assembly	8	4.1	4.50E-02
**GO terms for the 200 most P16-independent genes**			
**Biological process**	**Count**	**%**	**P value**
translation	54	27.3	7.70E-08
ribosome biogenesis	33	16.7	1.50E-05
ribonucleoprotein complex biogenesis	34	17.2	5.50E-05
ribosome assembly	11	5.6	1.10E-02
ribonucleoprotein complex assembly	13	6.6	4.70E-02
**GO terms for the 200 most K14-dependent genes**			
**Biological process**	**Count**	**%**	**P value**

thiamine biosynthetic process	7	3.5	5.40E-03
response to temperature stimulus	19	9.6	3.70E-03
thiamine and derivative biosynthetic process	7	3.5	2.50E-03
sulfur compound biosynthetic process	11	5.6	1.90E-03
thiamine metabolic process	7	3.5	2.00E-03
thiamine and derivative metabolic process	7	3.5	2.20E-03
water-soluble vitamin metabolic process	10	5.1	4.40E-03
sulfur metabolic process	12	6.1	4.20E-03
cellular response to heat	16	8.1	4.80E-03
vitamin metabolic process	10	5.1	4.90E-03
vitamin biosynthetic process	9	4.5	8.70E-03
water-soluble vitamin biosynthetic process	9	4.5	8.70E-03
response to heat	16	8.1	1.30E-02
oxidation reduction	22	11.1	1.50E-02
aromatic compound biosynthetic process	7	3.5	2.50E-02
response to abiotic stimulus	21	10.6	2.40E-02
response to toxin	7	3.5	4.60E-02
**GO terms for the 200 most P16-dependent genes**			
**Biological process**	**Count**	**%**	**P value**
sexual sporulation	16	8.2	4.10E-05
sexual sporulation resulting in formation of a cellular spore	16	8.2	4.10E-05
ascospore formation	16	8.2	4.10E-05
thiamine biosynthetic process	8	4.1	7.60E-05
thiamine and derivative biosynthetic process	8	4.1	7.50E-05
thiamine metabolic process	8	4.1	8.10E-05
reproductive developmental process	17	8.8	7.70E-05
thiamine and derivative metabolic process	8	4.1	7.60E-05
reproductive process in single-celled organism	17	8.8	3.90E-04
response to toxin	9	4.6	8.90E-04
reproductive cellular process	19	9.8	1.30E-03
sporulation resulting in formation of a cellular spore	18	9.3	1.20E-03
sporulation	18	9.3	1.20E-03
water-soluble vitamin metabolic process	10	5.2	1.40E-03
sexual reproduction	20	10.3	1.70E-03
aromatic compound biosynthetic process	8	4.1	1.90E-03
vitamin metabolic process	10	5.2	1.80E-03
vitamin biosynthetic process	9	4.6	3.50E-03
water-soluble vitamin biosynthetic process	9	4.6	3.50E-03
reproduction of a single-celled organism	17	8.8	3.30E-03
sulfur metabolic process	11	5.7	4.90E-03
sulfur compound biosynthetic process	9	4.6	8.70E-03
spore wall biogenesis	7	3.6	1.90E-02
spore wall assembly	7	3.6	1.90E-02
ascospore wall assembly	7	3.6	1.90E-02

ascospore wall biogenesis	7	3.6	1.90E-02
cell wall assembly	7	3.6	2.00E-02
response to temperature stimulus	15	7.7	2.10E-02
cellular response to heat	13	6.7	3.80E-02
pyridoxine metabolic process	4	2.1	3.70E-02
vitamin B6 metabolic process	4	2.1	3.70E-02
pyridoxine biosynthetic process	4	2.1	3.70E-02
vitamin B6 biosynthetic process	4	2.1	3.70E-02
fungal-type cell wall biogenesis	7	3.6	4.00E-02

Shown are the significantly enriched ($p < 0.05$) biological processes, corresponding gene counts and P values (Benjamini-Hochberg correction) for the 200 most K14/P16-independent and K14/P16-dependent genes after ranking according to the ratio of H3-normalised K4me3 (K14A or P16V relative to WT). GO analysis was performed using the DAVID functional annotation tool v. 6.7 (Huang da et al., 2009a, b).

Supplementary Experimental Procedures

Strains

S. cerevisiae strains and their genotypes used during this study are shown below.

Strain	Parent	Genotype	Origin
WTH3 (referred to as WT throughout)	-	*MAT**a** his3Δ200 leu2Δ0 lys2Δ0 trp1Δ63 ura3Δ0 met15Δ0 can1::MFA1pr-HIS3 hht1-hhf1::NatMX4 hht2-hhf2::[HHTS-HHFS]-URA3*	Open Biosystems
K14A	WTH3	*hht2-hhf2::[HHTS K14A-HHFS]-URA3*	Open Biosystems
K14R	WTH3	*hht2-hhf2::[HHTS K14R-HHFS]-URA3*	Open Biosystems
K14Q	WTH3	*hht2-hhf2::[HHTS K14Q-HHFS]-URA3*	Open Biosystems
RMY200	-	*MATa ade2-101 his3Δ200 lys2-801 trp1-Δ901 ura3-52 hht1 hhf1::LEU2 hht2 hhf2::HIS3 plus pRM200 [CEN4 ARS1 TRP1 HHT2-HHF2]*	(Mann and Grunstein, 1992)
RMY214 (K14R)	RMY200	*plus pRM214 [CEN4 ARS1 TRP1 hht2 K14R-HHF2]*	(Mann and Grunstein, 1992)
WZY63(a)	-	*MATa, ura3–52, lys2–801, ade2–101, trp163, his3200, leu21 hht1-hhf1::pWZ405-F2F9-LEU2,hht2-hhf2::pWZ403-F4F10-HIS3, plus pWZ414-F13 [HHT2-HHF2]*	(Zhang et al., 1998)
WZY67(a) (K14R)	WZY67(a)	*plus pWZ414-F43 [hht2 K14R-HHF2]*	(Zhang et al., 1998)
WT H3/H4	-	*MATa, leu2Δ1, his3Δ200, ura3-52, trp1Δ63, lys2-128δ, (hht1-hhf1)ΔLEU2 (hht2-hhf2)Δ::HIS3 Ty912Δ35-lacZ::his4, [pDM9-HHT1-HHF1-URA3] plus pWZ414-F12 [HHT2-HHF2]*	(Nakanishi et al., 2008)
P16A	WT H3/H4	*plus pWZ414 [hht2 P16A-HHF2]*	(Nakanishi et al., 2008)
P16V	WTH3	*hhts-hhfs::HHTS P16V-HHFS-KanMX6*	This study
K14A P16V	WTH3	*hhts-hhfs::HHTS K14A P16V-HHFS-KanMX6*	This study
K14R P16V	WTH3	*hhts-hhfs::HHTS K14R P16V-HHFS-KanMX6*	This study
K14Q P16V	WTH3	*hhts-hhfs::HHTS K14Q P16V-HHFS-KanMX6*	This study
WTH3 *jhd2Δ*	WTH3	*jhd2::KanMX6*	This study
K14A *jhd2Δ*	K14A	*jhd2::KanMX6*	This study
K14R *jhd2Δ*	K14R	*jhd2::KanMX6*	This study
K14Q *jhd2Δ*	K14Q	*jhd2::KanMX6*	This study
P16V *jhd2Δ*	P16V	*jhd2::LEU2*	This study
WTH3 Jhd2-HA	WTH3	*JHD2-3HA-KanMX6*	This study

K14A Jhd2-HA	K14A	*JHD2-3HA-KanMX6*	This study
K14R Jhd2-HA	K14R	*JHD2-3HA-KanMX6*	This study
K14Q Jhd2-HA	K14Q	*JHD2-3HA-KanMX6*	This study
P16V Jhd2-HA	P16V	*JHD2-3HA-KanMX6*	This study
K14A P16V Jhd2-HA	K14A P16V	*JHD2-3HA-KanMX6*	This study
K14R P16V Jhd2-HA	K14R P16V	*JHD2-3HA-KanMX6*	This study
K14Q P16V Jhd2-HA	K14Q P16V	*JHD2-3HA-KanMX6*	This study
WTH3 *spp1Δ*	WTH3	*spp1::TRP1*	This study
K14A *spp1Δ*	K14A	*spp1::TRP1*	This study
K14R *spp1Δ*	K14R	*spp1::TRP1*	This study
K14Q *spp1Δ*	K14Q	*spp1::TRP1*	This study
P16V *spp1Δ*	P16V	*spp1::TRP1*	This study
WTH3 *spp1Δ jhd2Δ*	WTH3 *jhd2Δ*	*spp1::TRP1 jhd2::KanMX6*	This study
K14A *spp1Δ jhd2Δ*	K14A *jhd2Δ*	*spp1::TRP1 jhd2::KanMX6*	This study
K14R *spp1Δ jhd2Δ*	K14R *jhd2Δ*	*spp1::TRP1 jhd2::KanMX6*	This study
K14Q *spp1Δ jhd2Δ*	K14Q *jhd2Δ*	*spp1::TRP1 jhd2::KanMX6*	This study
P16V *spp1Δ jhd2Δ*	P16V *jhd2Δ*	*spp1::TRP1 jhd2::KanMX6*	This study
WTH3 Spp1-HA	WTH3	*SPP1-3HA-KanMX6*	This study
K14A Spp1-HA	K14A	*SPP1-3HA-KanMX6*	This study
K14R Spp1-HA	K14R	*SPP1-3HA-KanMX6*	This study
K14Q Spp1-HA	K14Q	*SPP1-3HA-KanMX6*	This study
P16V Spp1-HA	P16V	*SPP1-3HA-TRP1*	This study
WTH3 Swd1-HA	WTH3	*SWD1-3HA-KanMX6*	This study
K14A Swd1-HA	K14A	*SWD1-3HA-KanMX6*	This study
K14R Swd1-HA	K14R	*SWD1-3HA-KanMX6*	This study
K14Q Swd1-HA	K14Q	*SWD1-3HA-KanMX6*	This study
P16V Swd1-HA	P16V	*SWD1-3HA-TRP1*	This study

Creation of the K14/P16 double substitution strains

The *HHTS-HHFS* locus was amplified from the Open Biosystems histone substitution strain (Dai et al., 2008). These fragments were then digested with *Eco*RI and *Spe*I at the endogenous restriction sites flanking the locus. The digested fragments were ligated into *Eco*RI/*Spe*I-digested pFA6 (KanMX6) (Longtine et al., 1998). Histone H3 double substitution strains were created using the singly-substituted H3 as templates for Quikchange site-directed mutagenesis (Stratagene). The resulting plasmids were confirmed by sequencing before use as PCR templates to replace the genomic *HHTS-HHFS* with the newly mutated locus. Transformed strains were sequenced once more before use in experiments.

Yeast growth conditions

Yeast cells were grown at 30°C, shaking at 200 rpm to exponential phase in YPD medium (1 % yeast extract (Difco), 1 % bactopeptone supplemented with 2 % glucose). Cells were grown overnight in 5 ml cultures before dilution to 0.2 OD_{600} (0.5 x 10^7 cells/ml) in the appropriate volume of fresh media. Cells were harvested during exponential growth at 0.5 OD_{600} (1.25 x 10^7 cells/ml) by centrifugation (3000 rpm, 5 min). The assessment of yeast growth by drop plate assays was performed by first normalising the cell number in overnight yeast cultures before pipetting 1.5 µl of five serial ten-fold dilutions onto YPD-agar plates. Plates were incubated for 2 days at 30°C.

Preparation of whole cell extracts

Cells were grown at 30°C in 25 ml of the appropriate media to 1.25 x 10^7 cells/ml. Whole cell extracts were prepared by vortexing the cells with glass beads in 300 µl 8 M urea, 240 µl loading buffer (100 mM Tris-Cl pH 6.8, 20 % glycerol, 4 % SDS, 0.1 % bromophenol blue) and 60 µl 1 M DTT for 3 min followed by boiling for 5 min.

Western blotting and dot blots

For Western blotting, proteins were separated on 10-15 % SDS-polyacrylamide gels and transferred to nitrocellulose membranes using a semi-dry transfer method. For dot blots, appropriate amounts of peptide were pipetted onto nitrocellulose membrane and allowed to dry. Membranes were blocked in 5 % bovine serum albumin (BSA) in TBST (20 mM Tris-Cl pH 7.5, 150 mM NaCl, 0.1 % TWEEN-20). Antibodies were added in 2.5 % BSA/TBST. The primary antibodies used are detailed in the table below. The HRP-conjugated rabbit, mouse

or rat secondary antibodies (Sigma) were used at 1:4000 dilutions. Interactions were visualised using chemiluminescence (Pierce) and exposure to X-ray film. Quantitation was performed using ImageJ software and signals were normalised to histone H3 levels and expressed relative to WT.

**Antibody**	**Company/ Catalogue number**	**Species reactivity**	**Dilution for Western blot**	**Dilution for ChIP**
H3	Millipore/07-690	Rabbit	1:2000	1:40
H3K4me2	Millipore/07-030	Rabbit	1:2000	1:40
H3K4me3	Millipore/05-745R	Rabbit	1:2000	1:40
H3K9ac	Millipore/07-352	Rabbit	1:2000	-
H3S10ph	Millipore/05-817	Rabbit	1:2000	-
H3K14ac	Millipore/07-353	Rabbit	1:2000	-
H3P16*cis*	-	Rabbit	1:7500	1:10
H3P16*trans*	-	Rabbit	1:5000	1:6.67
H3K18ac	Millipore/07-354	Rabbit	1:4000	1:40
H3K23ac	Millipore/07-355	Rabbit	1:2000	-
H3K27ac	Millipore/07-360	Rabbit	1:2000	-
H3K36me2	Millipore/07-369	Rabbit	1:2000	-
H3K36me3	Abcam/ab9050	Rabbit	1:2500	1:40
H3K79me2	Abcam/ab3594	Rabbit	1:2000	-
H3K79me3	Abcam/ab2621	Rabbit	1:2000	-
HA (for Western)	Roche/3F10	Rat	1:500	-
HA (for ChIP)	ab9110	Rabbit	-	1:40
Actin	Millipore/MAB1501	Mouse	1:1000	-

Raising polyclonal antibodies against P16$_{OH}$*cis* and P16$_{OH}$*trans* peptides.

Peptide synthesis and antibody production were performed by Pacific Immunology. Peptides were synthesised and the proline hydroxylated to allow the peptides to be enriched with the peptidyl-prolyl bond in either a *cis* or a *trans* conformation: CGKA-(*cis*-hydroxyproline)-RKQLA and CGKA-(*trans*-hydroxyproline)-RKQLA. Each peptide was conjugated to Keyhole Limpet Hemocyanin (KLH) carrier protein via the N-terminal cysteine and used to immunize rabbits. Final bleeds were taken after 105 days and the specificity was increased by purifying the sera against unmodified peptide. Antibodies required extensive

further purification. For the P16*cis* antibody, batches of antibody were purified by incubating 266.66 µl of the Pacific Immunology affinity-purified antibody in 1733.33 µl FA-150 buffer (0.1% SDS, 1% Triton-X-100, 10 mM HEPES, 0.1% sodium deoxycholate, 150 mM NaCl, 1 mM AEBSF, EDTA-free protease inhibitor cocktail (Roche)) with 90 µg $P16_{OH}$*trans* and 90 µg unmodified H3 peptides dotted onto nitrocellulose. Purifications were carried out for 48 h at 4°C, with fresh peptide/nitrocellulose added after 24 h. For P16*trans*, 400 µl antibody (in 1600 µl FA buffer) was incubated with 90 µg $P16_{OH}$*cis* and 90 µg unmodified H3, and purification performed exactly as for the P16*cis* antibody.

P16 isomerase assay

The proline isomerase assay was performed as described (Fischer et al., 1984). 10 µl of 7.8 mM paranitroaniline (pNA) peptide solution dissolved in water were used per 1 ml assay reaction. The unmodified, acetylated and substituted peptides, corresponding to residues 12-16 of histone H3 (GGKAP) followed by phenylalanine and the pNA group, were synthesised by Proteogenix, France and NeoBiolab, USA. The progress of the assay was monitored in real time every 0.6 s for 20 s in a spectrophotometer at 395 nm. The average initial rate of release of the pNA chromogenic substrate from the peptide after addition of chymotrypsin was calculated.

Chromatin immunoprecipitation

ChIP was performed as described in (Morillon et al., 2005). Briefly, cells grown to OD_{600} 0.5 in 50 ml of appropriate media were fixed with 1 % formaldehyde in 45 ml PBS for 30 min at

22°C followed by addition of glycine to 125 mM for 5 min. Cell pellets were collected by centrifugation (3000 rpm, 5 min) before washing twice with 10 ml cold PBS. Cells were resuspended in 500 µl cold FA-150 buffer (10 mM HEPES pH 7.9, 150 mM NaCl, 0.1 % SDS, 0.1 % sodium deoxycholate, 1 % Triton X-100) and broken using 1 ml glass beads on a MagnaLyser (Roche) at 4°C. Sample volume was increased to 2 ml with FA-150 buffer before shearing of the fixed chromatin by sonication using a biorupter (Diagenode, 30 min, 1 min on, 20s off, medium setting). Chromatin was cleared by centrifugation (10 000 rpm, 15 min, 4°C) and incubated with antibody (amounts stated in the Table above) in 1.5 ml siliconised Eppendorf tubes for 15-20 h rotating at 4°C. Bound chromatin was immunoprecipitated for 90 min at 22°C with 50 µl protein A-Sepharose pre-blocked with sonicated salmon sperm DNA. Beads and attached chromatin were pelleted by centrifugation (2600 rpm, 1 min) and washed with TSE-150 buffer (20 mM Tris-Cl pH 8.0, 150 mM NaCl, 2 mM EDTA, 0.1 % SDS, 1 % Triton X-100) for 3 min, TSE-500 buffer (20 mM Tris-Cl pH 8.0, 500 mM NaCl, 2 mM EDTA, 0.1 % SDS, 1 % Triton X-100) for 3 min, LiCl buffer (0.25 M LiCl, 10 mM Tris-Cl pH 8.0, 1 mM EDTA, 1 % dioxycholate, 1 % NP-40) for 15 min and twice with TE, all at 22°C. After washing, chromatin was eluted from the beads for 30 min at 65°C with elution buffer (0.1 M NaHCO3, 1 % SDS). Addition of 350 mM NaCl and incubation for 3 h at 65°C reversed the cross-links before treatment of samples with RNase A for 1 h at 37°C and proteinase K overnight at 65°C. DNA was purified using a PCR-purification kit (Qiagen) and eluted in 400 µl 1 mM Tris-Cl pH 8.0. Input DNA was diluted accordingly. Real-time quantitative PCR (qPCR) was carried out using a Corbett Rotorgene and Sybr green mix (Bioline). Data ([IP - no antibody control]/input) were expressed as a percentage of the input (relative to WT) and normalised to levels of H3 where appropriate. The primers used are listed in the table below (unless otherwise stated, the *FMP27* 5' primer used was *FMP27* primer 2).

Primer	Forward sequence	Reverse sequence
ADH1 1	CGGTATACGGCCTTCCTTCC	GGGAACGAGAACAATGACGA
ADH1 2	GCTATACCAAGCATACAATC	GGCTTTGGAACTGGAATATC
ADH1 3	GCCACTGACGGTGGTGCTCA	GGCACCAGCTGGCATACCGA
FMP27 1	TCTTGGTGGTGTTGCAGTTG	GCTGTTTGCTTTTACTGTCC
FMP27 2	CCGTCAGGCTAAAATCCGTT	CCTCTCTTGGTAATTCTTTA
FMP27 3	GGGTAACAATCTTATGGAAG	CCTTAATATTGTATGCTCGT
FMP27 4	GCCAAATAATCGCACTCCCA	GCGTTGGAGATTCTACCATC
FMP27 5	GGGATTTTATGATAACAGAG	GGGCACGAACAACGAGTAAT
FMP27 6	CGGAGCCTGAAGAACTTCGT	GGTCTGAAACATGGTAACA
PDC1	GTTTGCCAGGTGACTTCAAC	ACCGAAGGTGGTGATGATAC
PGK1	GCGTGTCTTCATCAGAGTTG	AGTGAGAAGCCAAGACAACG
RPL10	GAGCTGTTCCAGACTCCAAG	GTTGGCACAGATACGAGCAG
RPS15	ACGACCGATCATGTCTCAAG	TCTTCTAACTCTAGCTGGGG
SEN1	CCCAATAGCGATGTTCAGCT	CCCCTAATAATTTTGCCTCT

ChIP-sequencing and data analysis

Chromatin was immunoprecipitated essentially as described above except, where necessary, multiple immunoprecipitation reactions were performed and then pooled to obtain sufficient material for sequencing (10 ng). Immunoprecipitated DNA was quantified using Qubit Fluorometric Quantitation (Invitrogen). Sequencing was performed by the Wellcome Trust Centre for Human Genetics, Oxford. DNA was multiplexed during library preparation and subjected to 50 nt paired end single lane sequencing. Reads were aligned to the *S. cerevisiae* genome using Bowtie, allowing for up to two mismatches per read. Further analysis was performed using MATLAB (MathWorks). K4me3 was normalised to levels of histone H3 in each strain. To account for global changes in K4me3, the total read counts for K4me3/H3 in each strain were then normalised to the ratio of K4me3/H3 in the H3 substitution strain relative to the WT strain, as determined by immunoblotting (Zhang et al. 2011). Genome-wide H3-normalised K4me3 WT data was compared to other published datasets (Pokholok et al. (2005), ArrayExpress # E-WMIT-3, Kirmizis et al. (2007), GEO #

GSE8626) by dividing genes into 30 bins from the transcription start site (TSS) to the transcription termination site (TTS) (gene coordinates obtained from (Nagalakshmi et al., 2008), n= 6575) and calculating the average proportion of K4me3 as a fraction of the total K4me3 over the gene body. The most K14/P16-independent and K14/P16-dependent genes (n=200 for each of the four classes) were obtained by computing the average ratio of H3-normalised K4me3 in the H3 substitution strains over WT strain across the body of each non-dubious gene (TSS-TTS, n=5771). Subsequently, genes were ranked by the resultant ratio, whereby genes with the largest ratios were ranked at the top (rank =1). The K14/P16-independent genes are those with the highest 200 ratios of K4me3 (H3 substitution/WT) whereas the K14/P16-dependent genes are those with the lowest 200 ratios of K4me3. To produce the TSS-centred distribution plots, the ratios of H3-normalised K4me3 and log2 levels of Nrd1 (ChIP-seq data from (Mayer et al., 2012), ArrayExpress # E-MTAB-1060) were calculated for each gene and strain from 1500 bp upstream to 1500 bp downstream of the TSS and median ratios of K4me3/H3 determined for all genes and the 200 most K14/P16-independent and most K14/P16-dependent genes in both the K14A and P16V strains. Levels of nascent sense and antisense transcription (NET-seq data from (Churchman and Weissman, 2011), GEO # GSE25107) per base pair were calculated for each gene in a 300 bp window downstream of the TSS and plotted as a boxplot for all genes and the four described gene classes. Gene ontology analysis was performed using the DAVID functional annotation tool v 6.7 (Huang da et al., 2009a,b). P-values for the overlap analysis between the 200 most K14/P16-independent and K14/P16-dependent genes and genes regulated during the yeast metabolic cycle, environmental stress response or by SAGA/TFIID, or classed as RPG/Ribi were calculated from a simulated probability distribution curve.

Purification of histones from *S. cerevisiae* for mass spectrometry

Histones were prepared essentially as (Edmondson et al., 1996). Yeast cells were grown in 300 ml YPD to OD_{600} 0.5-0.8. Cells were harvested (5000 rpm JA-10 rotor, 5 min, 4°C) and washed in sterile water. Cells were resuspended in 0.1 mM Tris-Cl pH 9.4, 10 mM DTT and incubated for 15 min at 30°C with gentle shaking. Following a wash with and then resuspension in 1.2 M sorbitol, 20 mM HEPES pH 7.4, cells were spheroplasted with 2.75 g zymolyase/g yeast cells for 60 min at 30°C with gentle shaking. A series of washes was then performed in: ice-cold 1.2 M sorbitol, 20 mM PIPES pH 6.8, 1 mM $MgCl_2$; ice-cold nuclear isolation buffer (0.25 M sucrose, 60 mM KCl, 15 mM NaCl, 5 mM $MgCl_2$, $CaCl_2$, 15 mM MES pH 6.6, 0.8 % Triton X-100, protease inhibitors) twice with incubations on ice for 20 min; buffer A (10 mM Tris-Cl pH 8.0, 0.5 % NP-40, 75 mM NaCl, 30 mM sodium butyrate, protease inhibitors) twice with 15 min incubations on ice; and buffer B (10 mM Tris-Cl pH 8.0, 0.4 M NaCl, 30 mM sodium butyrate, protease inhibitors) twice with 10 min incubation on ice for the first wash. The resulting nuclei were resuspended in cold 0.4 N H_2SO_4 and incubated on ice for 30 min. After centrifugation (13 000 rpm, 10 min, 4°C), the supernatant (containing histones) was added to 5 volumes of acetone. Proteins were precipitated overnight at -20°C. Precipitated protein was collected by centrifugation (13 000 rpm, 10 min, 4°C) and air-dried before resuspension in 10 mM Tris-Cl pH 8.0. Samples were separated using 18 % SDS-polyacrylamide gel electrophoresis before excision of an appropriate molecular weight gel band and submission for mass spectrometry (Central Proteomics Facility, Dunn School of Pathology, Oxford).

Identification of histone H3 post-translational modifications by MS/MS

Histones were prepared for mass spectrometry essentially as described (Edmondson et al., 1996) and excised after 18 % SDS-polyacrylamide gel electrophoresis. The excised gel band was digested with trypsin overnight at 37°C. The resulting peptides were then analysed on an Ultimate 3000 nano HPLC system (Dionex, Camberley, UK) run in direct injection mode coupled to a LTQ XL Orbitrap mass spectrometer (Thermo Electron, Hemel Hempstead, UK; Central Proteomics Facility, Dunn School of Pathology, Oxford. Samples were resolved on a 15 cm x 75 µm inner diameter PicoTip analytical column (New Objective, Woburn, MA, USA), which was packed in-house with Reprosil-Pur C18-AQ phase, 3 µm bead (Dr. Maisch, Germany). A 120 min gradient was used to separate the peptides. The mass spectrometer was operated in a "Top 5" data-dependent acquisition mode. Precursor scans were performed in the Orbitrap at a resolving power of 60,000, from which five precursor ions were selected and fragmented in the linear ion trap. Charge state +1 ions were rejected. Data analysis was performed using Mascot v. 2.2 against a custom database containing the NCBI *S. cerevisiae* protein database and the substituted (P16V) histone H3 protein sequence. The search was performed with fixed carbamidomethyl modification and variable oxidation, acetyl(K), methyl(K), dimethyl(K) and trimethyl(K) modifications. The peptide mass tolerance (± 20 ppm), the fragment mass tolerance (± 0.5 Da) and the maximum number of missed cleavages (2) were defined in the Mascot search parameters. Peak intensities for the peptides containing K14ac in the WT and P16V samples were then calculated and compared. Peak intensities for the peptides containing K9ac and K23ac, which remain unchanged between the WT and P16V strains (as assessed by Western blotting), were used to control for loading and differences in ionisation caused by the P16V substitution.

Peptides for bromodomain interaction studies

Peptides were synthesized at GL Biochem (Shanghai) LTD, China and Proteogenix, France. Purity was determined with MALDI-TOF mass spectrometry and HPLC and the peptides used in these experiments had a minimum purity of 84 %. The peptide sequences are listed in the table below.

**Peptide**	**Sequence**	**MW (Da)**
Unmodified	TGGKAPRKQLASKAARC	1743.07
K18ac	TGGKAPR(Kac)QLASKAARC	1785.11
P16V-K18ac	TGGKAVR(Kac)QLASKAARC	1787.12
K14R-K18ac	TGGRAPR(Kac)QLASKAARC	1812.12
K14A-K18ac	TGGAAPR(Kac)QLASKAARC	1728.01
K14R-P16V-18ac	TGGRAVR(Kac)QLASKAARC	1815.14
K14ac	TGG(Kac)APRKQLASKAARC	1785.11
K14ac-K18ac	TGG(Kac)APR(Kac)QLASKAARC	1827.14

Spt7 bromodomain expression and purification

Spt7 bromodomain (amino acids 363-619) was cloned by PCR from genomic yeast DNA and then sub-cloned into expression vector pIVEX2.3d. The predicted bromodomain binding site residues Y500, Y520 and N521 were mutated to alanine by site-directed mutagenesis (QuickChange II) according to manufacturer's instructions (Agilent Technologies, UK). Proteins were expressed in *E. coli* BL21 T7 I^q LysY cells (NEB, UK) and purified on 1 ml HisTrap columns (GE Healthcare, UK) using a step-wise gradient of imidazole (40-500 mM) according to conditions described previously (Boubriak et al., 2009). Proteins were further purified by gel filtration on Superdex 75 column in 10 mM Hepes, 150 mM NaCl, 2 mM EDTA and 1 mM TCEP buffer and appropriate fractions concentrated on Vivaspin 6 devices.

Identity and purity of the protein was confirmed by N-terminal sequencing (AltaBiosciences, UK) and TOF MS ES+ mass spectrometry, and the molecular weight for the intact bromodomain protein was 31.1 kDa, as predicted.

Peptide pulldowns

SulfoLink Coupling Resin from Thermo Scientific, UK (Cat 20401) was used in all experiments. Peptides were coupled to the resin according to the manufacturer's instructions with some modification. Briefly, 2 ml of resin slurry per sample was prepared in 15 ml Falcon tubes. 2 ml of 0.5 mg/ml peptide in coupling buffer (50 mM Tris-Cl, 5 mM EDTA, pH 8.5) with 25 mM TCEP was immobilised to resin during 15 min rotation, followed by 30 min stand. After washing twice with coupling buffer, uncoupled sites where blocked by addition of 2 ml of freshly prepared 50 mM L-Cysteine-HCL solution. Resin pellet was further re-suspended and washed in washing buffer (1 M NaCl) and stored in PBS. Before use coupled resin was resuspended in binding buffer (40 mM HEPES, 175 mM NaCl, 0.1 % Tween 20, 10 % glycerol) and blocked with BSA (5 mg/ml). In each experiment 200 µl of resin was incubated with 350 µl of bromodomain (diluted with binding buffer to final concentration of 30 µg/ml) for 2 h at 4°C. Unbound protein was collected from the beads and after multiple washes with binding buffer, high salt (350 mM NaCl) and TE buffer, bound material was eluted by heating at 80°C with 120 µl LDS (Invitrogen)

SPR interaction studies

SPR data for peptides binding to immobilized Spt7 bromodomain was generated on Biacore T100 and T200 instruments at 25°C. In all cases S NTA chips were used to immobilise C-terminal His-tagged bromodomain via Ni^{2+} capture of the affinity tag. Typically bromodomain in 10 mM HEPES, 150 mM NaCl buffer at pH 7.4 was injected at 60 µg/ml resulting in 1.0 K RU – 4K RU of protein immobilised. After priming of the flow cells of the NTA chip, the binding of peptides dissolved in HBS-P+ running buffer (GE Healthcare, UK) in the concentration range from 1-200 µM was measured. For regeneration of the chip surface in addition to the standard 350 µM EDTA solution, 1 M imidazole solution was used. Sensorgrams, binding curves and K_d values were analysed with BIA T100 and T200 evaluation software (GE Healthcare, UK) using a 1:1 binding model.

Supplemental references

Boubriak, I., Mason, P.A., Clancy, D.J., Dockray, J., Saunders, R.D., and Cox, L.S. (2009). DmWRNexo is a 3'-5' exonuclease: phenotypic and biochemical characterization of mutants of the Drosophila orthologue of human WRN exonuclease. Biogerontology *10*, 267-277.

Churchman, L.S., and Weissman, J.S. (2011). Nascent transcript sequencing visualizes transcription at nucleotide resolution. Nature *469*, 368-373.

Dai, J., Hyland, E.M., Yuan, D.S., Huang, H., Bader, J.S., and Boeke, J.D. (2008). Probing nucleosome function: a highly versatile library of synthetic histone H3 and H4 mutants. Cell *134*, 1066-1078.

Edmondson, D.G., Smith, M.M., and Roth, S.Y. (1996). Repression domain of the yeast global repressor Tup1 interacts directly with histones H3 and H4. Genes Dev *10*, 1247-1259.

Gasch, A.P., Spellman, P.T., Kao, C.M., Carmel-Harel, O., Eisen, M.B., Storz, G., and Botstein, D. (2000). Genomic expression programs in the response of yeast cells to environmental changes. Mol Biol Cell *11*, 4241-4257.

Huang da, W., Sherman, B.T., and Lempicki, R.A. (2009a). Bioinformatics enrichment tools: paths toward the comprehensive functional analysis of large gene lists. Nucleic Acids Res *37*, 1-13.

Huang da, W., Sherman, B.T., and Lempicki, R.A. (2009b). Systematic and integrative analysis of large gene lists using DAVID bioinformatics resources. Nat Protoc *4*, 44-57.

Huisinga, K.L., and Pugh, B.F. (2004). A genome-wide housekeeping role for TFIID and a highly regulated stress-related role for SAGA in Saccharomyces cerevisiae. Mol Cell *13*, 573-585.

Kirmizis, A., Santos-Rosa, H., Penkett, C.J., Singer, M.A., Vermeulen, M., Mann, M., Bahler, J., Green, R.D., and Kouzarides, T. (2007). Arginine methylation at histone H3R2 controls deposition of H3K4 trimethylation. Nature *449*, 928-932.
Longtine, M.S., McKenzie, A., Demarini, D.J., Shah, N.G., Wach, A., Brachat, A., Philippsen, P., and Pringle, J.R. (1998). Additional modules for versatile and economical PCR-based gene deletion and modification in *Saccharomyces cerevisiae*. Yeast *14*, 953-961.
Mann, R.K., and Grunstein, M. (1992). Histone H3 N-terminal mutations allow hyperactivation of the yeast GAL1 gene in vivo. Embo J *11*, 3297-3306.
Mayer, A., Heidemann, M., Lidschreiber, M., Schreieck, A., Sun, M., Hintermair, C., Kremmer, E., Eick, D., and Cramer, P. (2012). CTD tyrosine phosphorylation impairs termination factor recruitment to RNA polymerase II. Science *336*, 1723-1725.
Morillon, A., Karabetsou, N., Nair, A., and Mellor, J. (2005). Dynamic lysine methylation on histone H3 defines the regulatory phase of gene transcription. Mol Cell *18*, 723-734.
Nagalakshmi, U., Wang, Z., Waern, K., Shou, C., Raha, D., Gerstein, M., and Snyder, M. (2008). The transcriptional landscape of the yeast genome defined by RNA sequencing. Science *320*, 1344-1349.
Nakanishi, S., Sanderson, B.W., Delventhal, K.M., Bradford, W.D., Staehling-Hampton, K., and Shilatifard, A. (2008). A comprehensive library of histone mutants identifies nucleosomal residues required for H3K4 methylation. Nat Struct Mol Biol *15*, 881-888.
Pokholok, D.K., Harbison, C.T., Levine, S., Cole, M., Hannett, N.M., Lee, T.I., Bell, G.W., Walker, K., Rolfe, P.A., Herbolsheimer, E.*, et al.* (2005). Genome-wide map of nucleosome acetylation and methylation in yeast. Cell *122*, 517-527.
Tu, B.P., Kudlicki, A., Rowicka, M., and McKnight, S.L. (2005). Logic of the yeast metabolic cycle: temporal compartmentalization of cellular processes. Science *310*, 1152-1158.
Zhang, L., Ma, H., and Pugh, B.F. (2011). Stable and dynamic nucleosome states during a meiotic developmental process. Genome Res *21*, 875-884.
Zhang, W., Bone, J.R., Edmondson, D.G., Turner, B.M., and Roth, S.Y. (1998). Essential and redundant functions of histone acetylation revealed by mutation of target lysines and loss of the Gcn5p acetyltransferase. Embo J *17*, 3155-3167.
